# Identifying contexts and mechanisms in multiple behavior change interventions affecting smoking cessation success: a rapid realist review

**DOI:** 10.1186/s12889-020-08973-2

**Published:** 2020-06-12

**Authors:** Nadia Minian, Tricia Corrin, Mathangee Lingam, Wayne K. deRuiter, Terri Rodak, Valerie H. Taylor, Heather Manson, Rosa Dragonetti, Laurie Zawertailo, Osnat C. Melamed, Margaret Hahn, Peter Selby

**Affiliations:** 1 Centre for Addiction and Mental Health, 175 College St, Toronto, Ontario M5T 1P7 Canada; 2 Department of Family and Community Medicine, University of Toronto, 500 University Avenue, Toronto, Ontario M5G 1V7 Canada; 3 Department of Psychiatry, University of Calgary, 1403 - 29 Street NW, Calgary, Alberta T2N 2T9 Canada; 4 Public Health Ontario, 480 University Avenue, Toronto, Ontario M5G 1V2 Canada; 5 Department of Pharmacology and Toxicology, University of Toronto, 1 King’s College Circle, Toronto, Ontario M5S 1A8 Canada; 6 Department of Psychiatry, University of Toronto, 250 College Street, Toronto, Ontario M5T 1R8 Canada; 7 Dalla Lana School of Public Health, University of Toronto, 155 College Street, Toronto, Ontario M5T 3M7 Canada

**Keywords:** Realist review, Multiple health behaviour interventions, Smoking cessation, COM-B

## Abstract

**Background:**

Smoking continues to be a leading cause of preventable chronic disease-related morbidity and mortality, excess healthcare expenditure, and lost work productivity. Tobacco users are disproportionately more likely to be engaging in other modifiable risk behaviours such as excess alcohol consumption, physical inactivity, and poor diet. While hundreds of interventions addressing the clustering of smoking and other modifiable risk behaviours have been conducted worldwide, there is insufficient information available about the context and mechanisms in these interventions that promote successful smoking cessation**.** The aim of this rapid realist review was to identify possible contexts and mechanisms used in multiple health behaviour change interventions (targeting tobacco and two or more additional risk behaviours) that are associated with improving smoking cessation outcome.

**Methods:**

This realist review method incorporated the following steps: (1) clarifying the scope, (2) searching for relevant evidence, (3) relevance confirmation, data extraction, and quality assessment, (4) data analysis and synthesis.

**Results:**

Of the 20,423 articles screened, 138 articles were included in this realist review. Following Michie et al.’s behavior change model (the COM-B model), capability, opportunity, and motivation were used to identify the mechanisms of behaviour change. Universally, increasing opportunities (i.e. factors that lie outside the individual that prompt the behaviour or make it possible) for participants to engage in healthy behaviours was associated with smoking cessation success. However, increasing participant’s capability or motivation to make a behaviour change was only successful within certain contexts.

**Conclusion:**

In order to address multiple health behaviours and assist individuals in quitting smoking, public health promotion interventions need to shift away from ‘individualistic epidemiology’ and invest resources into modifying factors that are external from the individual (i.e. creating a supportive environment).

**Trial registration:**

PROSPERO registration number: CRD42017064430

## Background

Smoking continues to be a leading cause of preventable chronic disease-related morbidity and mortality, excess healthcare expenditure, and lost work productivity [1–5]. While tobacco control efforts have made significant strides in reducing the overall prevalence of smoking in North America, millions of individuals report smoking in 2018 [6–8]. Furthermore, disparities in smoking remain prevalent across population groups [6].

Tobacco users are disproportionately more likely to be engaging in other modifiable risk behaviours such as excess alcohol consumption, physical inactivity, and poor diet [9]. A recent review identified strong associations between tobacco use and other modifiable risk behaviours [9], supporting an earlier report that only 12% of smokers had no other modifiable risk behaviours [10]. Tobacco users tend to consume more alcohol, eat less fruits and vegetables, and engage in fewer leisure time physical activity compared to non-tobacco users [11]. The clustering of these modifiable risk behaviours among tobacco users translates to a heightened risk for cardiovascular disease [12] as well as other chronic diseases and may also negatively influence the likelihood of successful smoking cessation [13–17].

Methods to improve cessation rates are of utmost importance as the percentage of tobacco users who are able to quit and maintain abstinence unaided is low, estimated at 3–5% annually [18]. Identifying and implementing smoking cessation interventions that are holistic and address other modifiable risk behaviours may improve quit outcomes and enhance overall quality of life. A Cochrane review of interventions that targeted multiple modifiable risk behaviours (including smoking) estimated a net reduction in smoking prevalence of 24% [19]. However, older guidelines for smoking cessation traditionally recommended only focusing on smoking cessation [20–23].

While hundreds of interventions addressing the clustering of smoking and other modifiable risk behaviours have been conducted worldwide [12, 13, 24–27], there is insufficient information available about mechanisms incorporated in these interventions that help to promote successful smoking cessation [19]. A rapid realist review, which emphasizes the contexts and mechanisms within the intervention that contribute to the outcomes, can provide a more in-depth understanding of how and why interventions are successful or unsuccessful [28, 29].

As a result, a rapid realist review was undertaken to analyze and characterize the various contexts and mechanisms within multiple health behaviour change interventions that contribute to successful smoking cessation. The mechanisms within interventions were characterized using the Capability, Opportunity, and Motivation Model of Behaviour (COM-B model), which states that an individual’s behaviour (B) is part of an interacting system involving 3 conditions: capability (C), opportunity (O), and motivation (M) [30]. COM-B is located at the centre of the Behaviour Change Wheel; which provides recommendations for efficient designs of effective behaviour change interventions [30]. COM-B can also be mapped to the Behaviour Change Technique (BCT) Taxonomy; which provides a systematic approach for designing interventions [30, 31]. The COM-B model, therefore, provides a foundation and starting point for intervention development.

The information gathered from this rapid realist review is intended to guide the curriculum and program development for Picking up the PACE (Promoting and Accelerating Change through Empowerment), a project funded by the Public Health Agency of Canada and the Medical Psychiatry Alliance. Picking Up the PACE aims to increase the capacity of practitioners to address other modifiable risk behaviours (e.g. physical inactivity, excessive alcohol use and poor diet) as a part of smoking cessation treatment. Picking Up the PACE aims to improve practitioner’s capacity to address multiple modifiable risk behaviours through two essential approaches: 1) developing an online training curriculum for healthcare practitioner outlining strategies and techniques for addressing multiple modifiable risk behaviours as a part of smoking cessation treatment and 2) designing a just-in-time clinical decision support system that will guide practitioners to address the engagement of multiple risk behaviours by their patients. The findings from this rapid realist review will also provide transferrable learnings for practitioners and decision-makers who are trying to develop multiple health behaviour change interventions.

In this paper, we report the findings of a rapid realist review of the current literature to produce a nuanced and critical understanding of how interventions for multiple modifiable risk behaviours increase smoking cessation outcomes. Specifically, the aim of this rapid realist review was to identify possible contexts and mechanisms used in multiple health behaviour change interventions (targeting tobacco and two or more additional risk behaviours) that are associated with improving smoking cessation outcome.

## Methods

### Rationale for a rapid realist review

Traditional approaches to literature reviews (systematic reviews and meta-analyses) assume outcomes are generated by linear causation [32]. While these approaches work well for studies conducted with highly controlled settings and exposures (e.g. randomized control trials); they severely limit our understanding of complex and pragmatic interventions [33]. Complex and pragmatic interventions require methods that offer a more comprehensive explanation of the ‘process’ that was undertaken [34]. Therefore, a realist synthesis is well-suited to meet these needs as it is uses a theory-driven approach to synthesize complex evidence from diverse sources and provide an understanding of why and how complex interventions works [28, 29].

Specifically, a realist synthesis aims to understand how, for whom, where, and why the intervention is effective or ineffective [28, 29]. This is accomplished by examining the “mechanisms”, exploring the “contexts” where the intervention occurred, and then linking these contexts and mechanisms to the “outcome” of the intervention [28]. As per the realist definition, mechanisms are the “underlying entities, processes, or [social] structures which operate in particular contexts to generate the outcomes of interest” [35]. This combination of the context (C), mechanisms (M), and outcome (O) in an intervention is called a C-M-O configuration. Recurrent patterns of C-M-O configurations are known as demi-regularities, or semi-predictable pattern/pathway of how a program functions. In other words, demi-regularities are a broad rule for how and when certain outcomes usually occur [28].

While full realist reviews can require a considerable dedication of time to the exploration of literature and subsequent analysis, rapid realist reviews (RRRs) have been used to enable a quicker transition from research to policy and/or practice [36]. Given the need for a timely synthesis and its application for the Picking Up the PACE programme, we undertook a rapid realist review; which allows us to maintain the core elements of the realist methodology and produce timely data.

Prior to this rapid realist review, a pre-specified protocol was registered (PROSPERO registration number: CRD42017064430) and published [37] which included the research question, search strategy, synthesis methodology, preliminary program theory, definitions, inclusion criteria for relevance screening, data extraction form, quality assessment tool, and plans for dissemination. An overview of the methods and any modifications to the original protocol are described below. Utilizing the RAMESES (Realist and Meta-narrative Evidence Syntheses: Evolving Standards) [38], and adapting it to follow a rapid realist review [34], the following steps were applied:

### Clarifying the scope

#### Identifying the research question

This rapid realist review supports a larger program, Picking Up the PACE, that aims to increase the ability of healthcare providers to offer evidence-based interventions to tobacco users which encompass changing modifiable risk behaviours (excess alcohol consumption, physical inactivity, poor diet, stress, and poor sleep) to ultimately achieve long-term smoking abstinence. As a result, this review focuses on smoking cessation in the context of multiple health behaviour change interventions that also address these other risk behaviours.

In order to clarify the scope of the rapid realist review, a multidisciplinary team with expertise in knowledge synthesis, public health, and multiple health behaviour change met in-person on nine occasions for 1 h over the course of 6 months. Our initial research question was: “What factors are associated with effective multiple health behaviour change (three or more behaviours including smoking)?”

#### Changes in the rapid realist review process

After a preliminary review of the data, further specificity of the study question was required to meet the desired outcome. The contexts and mechanisms involved in changing multiple health behaviours might be different than those involved in smoking cessation. Thus we modified our research question to: “What contexts and mechanisms are associated with improving smoking cessation outcome in interventions that target two or more additional unhealthy behaviours.”

#### Initial theory

We identified our initial theory of how, when, and why multiple health behaviour change interventions work by reviewing seven large-scale multi-factorial cardiovascular disease and cancer risk interventions [39–45]. These studies included the Multiple Risk Factor Intervention Trial (MRFIT) [39], the North Karelia Project [40], the Stanford Five City Project [41], Project PREVENT [43], the Minnesota Heart Health Program [45], the Mediterranean Lifestyle Trial [44], and the BETTER Trial [42], all of which are well-known studies that promoted multiple health behaviour change in large community samples [46]. As specified in our protocol manuscript [37], our preliminary review of these seven interventions involved having two independent reviewers extract the following information from the studies:

1. The specific activities within each intervention. Activities are physical/tangible tasks that were undertaken by the intervention (e.g. counselling, sharing of educational flyers, workshop, courses, prize draw). Please note, we coded all activities undertaken by intervention for any behaviour, not only those related to smoking.
2. The setting in which the intervention took place, including physical environment, social setting, and political climate (if provided).
3. The outcomes of each intervention, including any behavioural and/or clinical outcomes.

Through this preliminary review, we found that successful interventions usually had: pre-existing infrastructure that facilitates the delivery of the intervention, and targeting regions (e.g. geographic, population groups) where the need for the intervention is well-characterized. Furthermore, activities undertaken by these interventions often targeted the surrounding community and/or organizational structure. This multi-level approach appears to be in an effort to change the physical and social opportunities that can help facilitate multiple health behaviour change in individuals. Individual-level activities frequently focused on increasing patient’s awareness and knowledge, improving feelings of support, empowerment, and incorporating incentives for completing activities.

Upon closer review, we realized that these activities mapped onto the COM-B model; which stipulates that behaviour change requires change in one or more of the following component: capability, opportunity, and motivation [30]. All seven studies used in developing our initial program theory sought to change at least one component of this behavioural system. We used the taxonomy of behaviour change techniques [31] to code each activity specified in the studies and we cross-referenced these codes with the COM-B model. We used Table 2 in Michie and colleagues’ article to help us create the links between the components of the COM-B model and the BCT taxonomy [30]. For example an intervention that helped participants set a quit date was categorized as BCT 1.3“Goal setting” and consequently coded under “Capability” within the COM-B model. A visual depiction of this theory can be found in the published protocol [37].

The coded data was reviewed by our expert panel, which had a total of 11 members and was comprised of representatives from the Medical Psychiatry Alliance, Public Health Ontario, and the Centre for Addiction and Mental Health. Over the course of 9 in-person meetings, the expert panel assisted the research team with the review and development of the initial program theory.

### Searching for relevant evidence: search strategy and eligibility criteria

To test our program theory, a search strategy was developed and implemented to retrieve relevant primary data from both academic and grey literature. The search strategy was informed by the research team and developed by a medical librarian who executed the search across multiple bibliographic databases [37]. After our protocol was published, minor changes were made to the search strategy (see Additional File 1). The initial search aimed to identify as many multiple health behaviour interventions as possible, allowing the team to accurately identify trends across the literature.

To identify grey literature from Canada, Europe, and the USA, variations of the phrase “multiple health behaviours” were used to hand search the websites and online repositories of international, national, and provincial health organizations, health behaviour/condition-specific associations, clinical trial registries, and grey literature repositories. Reference lists of three systematic reviews and meta-analyses [27, 106, 107] were also hand searched to identify any relevant resources not captured by the systematic searches. No additional articles were included from the grey literature or reference list searches. After the search was complete, we chose to exclude books and reviews. It should be noted that 22 interventions were identified as having more than one publication reporting similar results. In these cases, the lead scientist and two additional members of the team selected one article per intervention to represent the contexts, mechanisms, and outcomes of the intervention, and excluded other articles associated with that intervention.

### Relevance confirmation, data extraction, and quality assessment

Two independent reviewers assessed each study to determine its relevance to our research question, extract pertinent information, and appraise its quality using pre-designed and pre-tested relevance screening and data extraction forms [37]. The systematic review software DistillerSR [108] was used for this process. As described in our protocol [37], to be included in this review the study had to:

- Describe interventions that targeted tobacco use as well as two or additional modifiable risk behaviours (excess alcohol consumption, physical inactivity, poor diet, stress, and poor sleep).
- Report on long-term (i.e. follow-up at 5 months or longer) smoking cessation outcomes

Since we had multiple study designs included in this rapid realist review, a combination of the Mixed Methods Appraisal Tool (MMAT) [109] and the Critical Appraisal Skills Programme (CASP) [110] was used to evaluate the methodological quality of qualitative, quantitative, and mixed method studies. Each type of study was assessed by two reviewers using a pre-designed quality assessment form [37]. As per MMAT and CASP appraisal methods, the quality criteria differed based on the study design; quantitative – randomized controlled trial (eight criteria), quantitative – non-randomized (10 criteria), qualitative (10 criteria), and mixed-method (six criteria). These criteria were scored using a nominal scale (Yes/No/Can’t Tell).

Based on the scoring metrics of the MMAT [109] and adjusting for the additional CASP criteria [110], an overall quality score was calculated for each study using the descriptors *, **, ***, and ****. For all types of studies, the score was derived by taking the number of criteria met and dividing it by the number of criteria. Scores were assigned the following descriptors: 0–25% (*), 26–50% (**), 51–75% (***), and 76% + (****). To score the mixed methods studies, the overall quality could not exceed the quality of the weakest section of the study. For example, in a mixed method study, if the qualitative score is (**), and the quantitative and mixed method scores are both (***), the study is assigned an overall score of the lowest section (**).The questions used to score each study can be found in Additional File 2.

Prior to data extraction and coding of the context, mechanisms, and outcomes within the studies, reviewers were trained on the COM-B model, the Behaviour Change Wheel and the BCT taxonomy [30, 31]. They were also trained on how to characterize the various techniques that are used within interventions and map these techniques onto the COM-B model. Once trained, the following process was also undertaken by the two independent reviewers:

- Review article to identify and record the activities that took place in the intervention.
- Code the modifiable risk behaviours the intervention was targeting.
- Code which techniques were applied to each activity, as defined by the BCT taxonomy. Please note, we coded all activities and corresponding techniques in the intervention, not only those that are specific to smoking cessation.
- Determine how each technique is associated with the COM-B model.
- Code the target population (e.g. gender, ethnicity, general public vs patients)
- Code the smoking outcome(s), including whether there was a statistically significant change and the follow-up period in which the outcome was assessed (e.g. end of treatment, 6 months, 12 months, or 24 months)
- Code the context where the intervention took place (e.g. region, clinical setting, clinical, community-based settings, and/or school-based settings)
- At each step, discrepancies between two reviewers were resolved by consensus or, when necessary, by a third reviewer.

### Data analysis and synthesis process

The data from DistillerSR [108] was exported to Microsoft Excel for descriptive analysis and analyzed using NVivo 11 [111]. To determine whether the intervention fit the initial program theory and to identify if there were any emerging patterns in the types and combination of C-M-O’s configurations used, the reviewers examined the studies to see whether the interventions had also focused on changed physical and social opportunities and/or other behaviour change techniques such as raising awareness, increasing knowledge, and encouraging empowerment. The behaviour change techniques did not have to be specific to smoking and could be targeting any modifiable risk behaviours. For example, if a multiple health behaviour change intervention offered membership to a gym to help improve physical activity, this was coded as Opportunity.

Smoking cessation outcomes were measured in a variety of ways across articles, including different time points (e.g. at end of treatment, 3 months, 12 months), duration of abstinence (e.g. 7-day point prevalence abstinence vs last 30 days), and presentation of data (e.g. descriptive vs statistical analyses). These outcomes were verified (e.g. biochemically) or were self-reported. The literature shows that both types of outcomes are valid [112–114] and therefore we did not differentiate between self-reported measures and biochemically verified measures. However, studies that used non-validated measures/screeners for self-report questions would be penalized in the quality assessment score. Please see Additional File 2 questions used to score each study.

As a result, we organized our findings by whether statistically significant smoking cessation outcomes were observed and whether the outcome was measured long-term (i.e. ≥5 months). Within these outcome types, the interventions were organized by the three categories that were then used to identify the mechanisms (capability, opportunity, and motivation) and the context in which the intervention occurred. Many of the reviewed articles did not describe the context in which the intervention was implemented in sufficient detail. Thus we decided to be as broad as possible and divided context into three categories: 1) the continent in which the intervention took place, 2) the type of setting (e.g. clinical, workplace) and 3) whether it was a multidisciplinary intervention. We established the following criteria to report demi-regularities:

- There were a minimum of three interventions using the specific C-M-O configuration.
- Among interventions with a specific C-M-O configuration, either ≥60% OR ≤ 40% of these interventions reported statistically significant increase in smoking cessation.

To present an example of how this process works, if we discover a C-M-O configuration (e.g. Clinical Setting – Capability – Smoking Cessation Outcome) within an intervention, there must be at least two other interventions with this C-M-O configuration to allow for further analysis. In this hypothetical example, if we have a total of ten interventions that have ‘Clinical Setting-Capability-Smoking Cessation Outcome’ configuration, we then have to determine what percentage of these studies reported a statistically significant increase in smoking cessation. In order for this C-M-O configuration to be categorized as a demi-regularity, at least 60% of these interventions must report a statistically significant increase in long-term smoking cessation outcome (i.e. ≥5 months). The demi-regularity in this case would be that interventions in clinical settings that target capability are more likely to lead to improvement in smoking cessation outcome. Alternatively, if ≤40% of the interventions reported a statistically significant increase in smoking cessation outcome, the demi-regularity would suggest that interventions in clinical settings that target capability are less likely to lead to improvements in smoking cessation outcomes.

In this paper, we analyzed demi-regularities in interventions that were rated four stars in our quality rating, used statistical analyses, and reported long-term smoking cessation outcomes (i.e. ≥5 months). We chose to only include those interventions with a four star rating as they have the least amount of bias. Once a demi-regularity was discovered, studies that had lower quality assessment scores (less than four stars), and/or did not perform statistical analyses were included in our pool for analysis to confirm if the previously observed demi-regularity persisted.

## Results

**Fig. 1:**
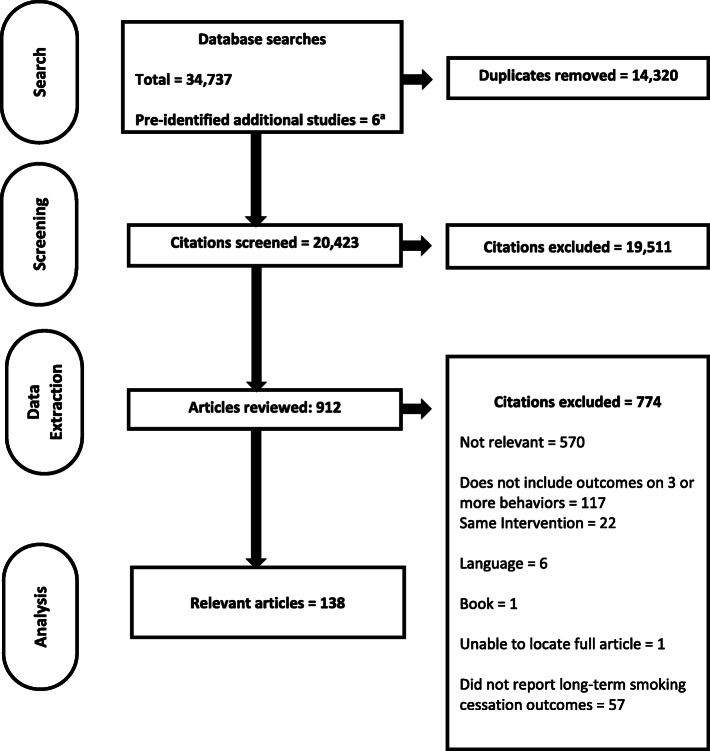
PRISMA flow diagram of articles through the rapid realist review process. ^a^There were a total of seven studies that were pre-identified for theory development; however one of the seven studies was published in 2013; which is within the timeframe for the literature search (2005–2017) and thus counted in this flow diagram as a part of the total number of studies from the literature search

The flow of information through the rapid realist review process is shown in Fig. 1.

A descriptive overview of all the interventions is provided in Table 1. Table 2 outlines the contexts, activities, and mechanisms used by interventions with statistically significant long-term (i.e. ≥5 months) smoking cessation outcomes.

**Table 1:** General Characteristics of 138 Included Articles

Category	Count (percentage)
*Date of Publication*	
2011–2017	85 (62%)
2005–2010	48 (35)
< 2005	5 (4)
*Continent*^*a,b*^	
Africa	2 (1)
Asia	18 (13)
Australasia^c^	9 (7)
Central/South America ^d^	1 (1)
North America^e^	49 (36)
Europe^f^	60 (43)
*Quality Assessment*^*b*^	
4 stars	66 (48)
3 stars	59 (43)
2 stars	13 (9)
*Study Design*^*a,b*^	
Cohort	44 (32)
Cross-sectional	5 (4)
Mixed method	4 (3)
Qualitative	4 (3)
Quasi-experimental	23 (17)
Randomized control trial	63 (46)
Other^g^	3 (2)
*Theories of Behaviour Change Used*^*a*^	
The Behaviour Change Approach	2 (1)
The Community Organization Approach	2 (1)
Goal-Systems Theory	1 (1)
Health Belief Model	1 (1)
Innovation-Diffusion Theory	3 (2)
Integrated Theory of Behaviour Change	1 (1)
Self-Regulation Theory	2 (1)
Social Cognitive Theory	8 (6)
Social-Ecological Theory	5 (4)
Social Learning Theory	6 (4)
Theory of Planned Behaviour	5 (4)
Transtheoretical Model	16 (12)

^a^ Total count sums to > 138 as studies can fall into more than one category

^b^ Due to rounding, total percentages do not equal 100

^c^ China, India, Iran, Korea, Turkey, United Arab Emirates, Indonesia, Sri Lanka, Vietnam, Japan, Malaysia, Taiwan, Pakistan, Singapore, and Uzbekistan

^d^ Brazil,Paraguay

^e^ Canada, Mexico, United States

^f^ Denmark, Finland, Italy, Netherlands, Spain, Sweden, United Kingdom, France, Poland, Sweden, Croatia, Lithuania, Belgium, Greece, Portugal, Norway, Malta, Switzerland

^g^ Case-control, case-study

**Table 2:** Characteristics of Interventions with Statistically Significant Smoking Cessation Outcomes^a^

Article	Location	Context - Setting	Target population	Type of prevention	Activities used in intervention	Mechanisms used in intervention
**3 Behaviour Changes Targeted – ALCOHOL, PHYSICAL ACTIVITY, SMOKING**						
Wang et al., 2015 [47]	Asia	Community Setting Rural Region	Adults with metabolic syndrome	Secondary	Education Group Interactions Tailored Feedback	Capability Motivation Opportunity
Mastrangelo et al., 2015 [48]	Europe	Workplace Intervention	Adult/elderly male employees	Primary and Secondary	Counselling Education	Capability Motivation
Jelinek et al., 2014 [49]	Australasia	Low Socio-Economic Status	Adult/elderly cardiovascular patients	Secondary	Counselling Tailored Feedback	Capability Opportunity
Saltychev et al., 2012 [50]	Europe	Clinical Setting Workplace Intervention	Adult employees	Primary	Group Interactions	Capability Motivation
Kim et al., 2016 [51]	North America	N/A	Adult/elderly cardiovascular patients	Secondary	Education Social Support	Capability Opportunity
**3 Behaviour Changes Targeted – ALCOHOL, DIET, SMOKING**						
Lopez et al., 2007 [52]	Europe	Clinical Setting	Adult patients attending primary care centers who have a first or second-degree relative affected by cancer.	Primary	Counselling Education Tailored Feedback	Capability Motivation
**3 Behaviour Changes Targeted – ALCOHOL, SMOKING, STRESS**						
Laaksonen et al., 2013 [53]	Europe	Clinical Setting	Adults	Secondary	Education Incentives Tailored Feedback Pharmacolo-gical Support	Capability Motivation Opportunity
**3 Behaviours Changes Targeted – DIET, PHYSICAL ACTIVITY, SMOKING**						
Gomez-Pardo et al., 2016 [54]	Europe	N/A	Adults with at least 1 cardiovascular risk factor	Primary	Community Organization Education Group Interactions Social Support	Capability Opportunity
Ofori et al., 2015 [55]	Europe	Clinical Setting	Adult/elderly cardiovascular patients	Primary and Secondary	Counselling Education Group Interactions Motivational Interviewing Pharmacolo-gical Support Social support Tailored Feedback	Capability Motivation Opportunity
Saraf et al., 2015 [56]	Asia	Rural Region School Intervention	Adolescent students	Primary	Community Organization Education Incentives Social Support	Capability Motivation Opportunity
Eckman et al., 2012 [57]	North America	Clinical Setting	Adult/elderly cardiovascular patients	Secondary	Education	Capability
Ruffin et al., 2011 [58]	North America	Clinical Setting	Adults in the general public	Primary	Tailored Feedback	Motivation
O’Brien et al., 2010 [59]	North America	Community Setting School Intervention	Adolescent students	Primary	Community Organization Counselling Education	Capability Opportunity
Wendel-Vos et al., 2009 [60]	Europe	Community Setting Low Socio-Economic Status	Adults in the general public	Primary	Education Group Interactions Mass Media Campaigns	Capability Opportunity
Plescia et al., 2008 [61]	North America	Clinical Setting Community Setting	Adult/elderly urban, African American community.	Primary	Community Organization Education Group Interaction Mass Media Campaigns	Capability Opportunity
Schumacher et al., 2006 [62]	Europe	Clinical Setting	Adult/elderly cardiovascular patients	Secondary	Counselling Education Group Interactions	Capability Opportunity
Becker et al., 2005 [63]	North America	Community Setting	African American adult siblings of individuals hospitalized for coronary heart disease	Primary	Counseling Incentives Pharmacolo-gical Support	Capability Motivation Opportunity
Friesen et al., 2010 [64]	North America	Community Setting School Intervention Workplace Intervention Rural Region	Adults/elderly from general public	Primary	Community Organization Education Group Interactions Mass Media Campaigns	Capability Opportunity
Shalaeva et al., 2017 [65]	Asia	Clinical Setting	Adult/elderly type 2 diabetes patients having undergone trans-femoral amputation (TFA)	Secondary	Education Incentives Tailored Feedback	Capability Opportunity
Monteagudo et al., 2013 [66]	Europe	Clinical Setting	Adult/elderly patients with COPD	Secondary	Counselling Education Motivational Interviewing	Capability Motivation Opportunity
Arikan et al., 2011 [67]	Asia	Rural Region	Adults from general public	Primary	Education Tailored Feedback	Capability
Richardson et al., 2008 [68]	Europe	Community Setting	Adults from general public	Primary	Education Tailored Feedback	Capability Opportunity
Naser et al., 2008 [69]	Asia	Clinical Setting	Adult cardiovascular patients	Secondary	Counselling Education Tailored Feedback	Capability Motivation
Smith et al., 2007 [70]	Europe	Clinical Setting	Adults with severe mental illness	Secondary	Education Group Interactions	Capability Opportunity
Dendana et al., 2017 [71]	Africa	School Intervention	Adolescent students	Primary	Education Group Interaction Opinion Leaders	Capability Opportunity
Gamble et al., 2012 [72]	North America	Clinical Setting Community Setting	Adult/elderly high-risk type 2 diabetic patients with hypertension and/or albuminuria	Tertiary	Tailored Feedback	Capability
Sadeghi et al., 2011 [73]	Asia	Community Setting	Adult/elderly females from general public	Primary	Education Mass Media Campaign	Capability Opportunity
Lando et al., 1995 [74]	North America	Community Setting Workplace Intervention	Adult/elderly from general public	Primary	Community Organization Education Mass Media Campaign Social Support Tailored Feedback	Capability Motivation Opportunity
Farquhar et al., 1990 [41]	North America	Community Setting School Intervention	Adults from general public	Primary	Education Incentives	Capability Motivation
**3 Behaviours Changes Targeted – PHYSICAL ACTIVITY, SMOKING, STRESS**						
Baker et al., 2015 [12]	Europe	Clinical Setting	Adult/elderly cardiovascular patients	Secondary Prevention	Counselling	Capability
**4 Behaviours Changes Targeted – ALCOHOL, DIET, PHYSICAL ACTIVITY, SMOKING**						
Xavier et al., 2016 [75]	Asia	Clinical Setting	Adult/elderly cardiovascular patients	Secondary	Counselling Education	Capability Opportunity
Shin et al., 2015 [76]	Asia	N/A	Patients with diabetes	Secondary	Education	Capability
Van Den Wijngaart et al., 2015 [77]	Europe	Clinical Setting	Adult/elderly cardiovascular patients	Secondary	Motivational Interviewing Tailored Feedback	Motivation
Schulz et al., 2014 [78]	Europe	N/A	Adults from general public	Primary	Education Tailored feedback	Capability Motivation
Busch et al., 2013 [79]	Europe	School Intervention	Adolescent students	Primary	Education Group Interactions	Capability Motivation Opportunity
Fernald et al., 2012 [80]	North America	Clinical Settings	Adults from general public	Primary	Community Organization Counselling Education	Capability Opportunity
Tobari et al., 2010 [81]	Asia	Clinical Settings Community Settings	Adults/elderly cardiovascular patients	Secondary	Counselling Education Tailored Feedback	Capability
Holtrop et al., 2008 [82]	North America	Clinical Setting	Adults from general public	Primary	Counselling Education Motivational Interviewing Tailored Feedback	Capability Motivation Opportunity
Frank et al., 2007 [83]	North America	School Intervention	Adult students	Primary	Counselling Education	Capability Opportunity
Cox et al., 2005 [84]	North America	Clinical Setting	Adult cancer patients	Tertiary	Counselling Education	Capability Motivation
Wills et al., 2017 [85]	Europe	School Intervention	Adult students	Primary	Education	Capability
Dale et al., 2016 [86]	Europe	School Intervention	Disadvantaged adults/adoles-cents	Primary	Counselling Group Interactions Motivational Interviewing Social Support Tailored Feedback	Capability Motivation
Zhou et al., 2010 [87]	Asia	Rural Region	Rural Chinese, Asian/Pacific Islander adult/elderly	Primary and Secondary	Counselling Motivational Interviewing Social Support Tailored Feedback	Capability Motivation Opportunity
Kadda et al., 2016 [88]	Europe	Clinical Setting	Adult/elderly cardiovascular patients	Tertiary	Counselling Education Social Support	Capability Motivation Opportunity
Chander et al., 2013 [89]	Asia	Clinical Setting Low Socio-Economic Status	Adult/elderly cardiovascular patients with diabetes	Secondary	Counselling Education Group Interactions	Capability
Schilling et al., 2005 [90]	Europe	Workplace Intervention	Adult employees	Primary	Education Tailored Feedback	Capability
**4 Behaviours Changes Targeted – DIET, PHYSICAL ACTIVITY, SMOKING, STRESS**						
Park et al., 2015 [91]	Asia	N/A	Asian/Pacific Islanders adult/elderly cardiovascular patients	Secondary	Counselling Education Incentives Social Support	Capability Motivation Opportunity
Gibson et al., 2014 [92]	Europe	Community Setting	Adults with diabetes and 2 other risk factors (smoking, hypertension or dyslipidaemia)	Secondary	Education Group Interaction Motivational Interviewing Social Support Tailored Feedback Pharmacolo-gical Support	Capability Motivation Opportunity
Siddiqui et al., 2012 [93]	Europe	Workplace Intervention	Adult employees	Primary	Counselling Incentives Motivational Interviewing	Capability Opportunity
Clouse et al., 2012 [94]	North America	Community Setting Prison/Jail	Adult incarcerated males (residents of a correctional substance abuse program)	Primary	Community Organization Education Group Interactions Opinion Leaders Mass Media Campaigns	Capability Motivation Opportunity
Jolly et al., 2007 [95]	Europe	Clinical Setting Low Socio-Economic Status	Adult/elderly cardiovascular patients	Secondary	Counselling Education	Capability Motivation Opportunity
Long et al., 2010 [96]	North America	Workplace Intervention	Adult employees	Primary	Counselling Education Group Interactions Incentives Pharmacolo-gical Support Opinion Leaders Tailored Feedback	Capability Motivation Opportunity
Niederhauser et al., 2005 [97]	North America	Community Setting `	Active duty infantry soldiers and their spouses	Primary	Community Organization Counselling Education Incentives	Capability Motivation Opportunity
White et al., 2015 [98]	North America	Workplace Intervention	Adult employees	Primary	Counselling Education Tailored Feedback	Capability Motivation
Chaves et al., 2015 [99]	Central America/South America/Caribbean	Clinical Setting	Adult/elderly from general public	Primary and Secondary	Counselling Education Social Support	Capability
Kelishadi et al., 2011 [100]	Asia	Clinical setting Community Setting School Intervention	Adults from general public	Primary and Secondary	Community Organization Education Mass Media Campaigns Opinion Leaders	Capability Opportunity
Puska et al., 1985 [40]	Europe	Clinical Setting Community Setting School Intervention	Adults from general public	Primary	Community Organization Education Mass Media Campaigns Opinion Leaders Social Support	Capability Motivation Opportunity
**5 Behaviours Changes Targeted– ALCOHOL, DIET, PHYSICAL ACTIVITY, SMOKING, STRESS**						
Baker et al., 2009 [101]	Australasia	N/A	Adult smokers with non-acute psychiatric disorders	Primary	Counselling Education Incentives Motivational Interviewing Pharmacolo-gical Support Tailored Feedback	Capability Motivation Opportunity
Byrne et al., 2011 [102]	North America	Workplace Intervention	Adult/elderly employees	Primary	Community Organization Counselling Education Group Interactions Incentives Tailored Feedback	Capability Motivation Opportunity
Henke et al., 2011 [103]	North America	Workplace Intervention	Adult employees	Primary	Community Organization Incentives Tailored Feedback	Capability Motivation Opportunity
Goetzel et al., 2014 [104]	North America	Workplace Intervention	Adult employees	Primary	Community Organization Counselling Education Tailored Feedback	Capability Motivation
**6 Behaviours Changes Targeted – ALCOHOL, DIET, PHYSICAL ACTIVITY, SLEEP, SMOKING, STRESS**						
Kuehl et al., 2016 [105]	North America	Workplace Intervention	Adult employees	Primary	Education Group Interactions Social Support	Capability Motivation Opportunity

^a^N/A stands for not available. There was no information available from the article for the specific category

Exploration of the differences and commonalities among the interventions reveals several trends. For example, all interventions that took place in Africa (*n* = 2) addressed only three behaviours and these behaviours did not include alcohol, stress, or sleep. Furthermore, none of the interventions in Africa use motivation as a mechanism. There was only one multiple health behaviour change intervention that took place in Central/South America (*n* = 1). This intervention was conducted in a clinical setting and was designed to address four behaviours simultaneously. On the other hand, Europe (*n* = 60) and North America (*n* = 49) had larger variations in the number and types of behaviours addressed by any given intervention. Europe and North America were the only continents in which sleep was also targeted within behavioural change interventions. North America was also the only region in which there were interventions that targeted all six modifiable risk behaviours simultaneously.

Overall, the majority of interventions employed at least two mechanisms. Specifically 31(22%) interventions only used one mechanism, 66 (48%) interventions used two mechanisms, and 41 (30%) targeted all three mechanisms. As shown in Tables 1, 66 studies (48%) were scored as 4 stars, 59 (43%) were three stars, and 13 (9%) were scored as two stars. Common reasons why studies scored less than four stars included: lack of clarity around whether bias was sufficiently addressed, use of non-validated measures, insufficient description of randomization process (if applicable), and high withdrawal/drop-out.

### Demi-regularity – opportunity

For the purposes of this rapid realist review, “opportunity” was defined as “all the factors that lie outside the individual that make the behaviour possible or prompt it” [30]. When interventions focused on increasing the “opportunity” to access services and change the social environment, tobacco users who engaged in other unhealthy behaviours were more likely to achieve long-term smoking cessation. In particular, interventions that: 1) provided “access” to healthy living tools (e.g. free medications such as nicotine replacement therapy, gym memberships, walking groups, free/accessible fruits and vegetables, etc.) and/or 2) encouraged “social support” (e.g. incorporating family members into care, interventions held social events).

#### Supporting evidence

There were 32 interventions [40, 49, 51, 54–56, 61, 63, 65, 71, 75, 82, 83, 92, 95, 96, 101, 103, 105, 115–127] that used opportunity as one of the mechanisms for behaviour change with the majority of these interventions (59%) [40, 49, 51, 54–56, 61, 63, 65, 71, 75, 82, 83, 92, 95, 96, 101, 103, 105] reporting successful long-term cessation. There were 12 interventions that aimed to increase access to resources as a part of the intervention [49, 56, 61, 63, 82, 83, 92, 95, 96, 103, 119, 121]. Of these, 10 (83%) interventions reported successful long-term smoking cessation [49, 56, 61, 63, 82, 83, 92, 95, 96, 103]. The majority of interventions that made changes to the physical and/or social environment (8/11; 73%) [40, 56, 61, 71, 83, 96, 103, 105] or interventions that improved patient’s social support system (10/15; 67%) [51, 54, 55, 65, 75, 82, 96, 101, 103, 105] also reported successful long-term cessation.

In various settings (e.g. clinical settings, community settings, workplace, etc.) and across several continents, programs that aimed to increase the opportunity to change behaviours were successful in achieving long-term smoking abstinence among their participants (Table 3). These trends remain fairly consistent when examining all interventions; including those interventions that were given a rating from one to three stars in our quality assessment and reported statistical significance (see Additional File 3).

**Table 3:** Interventions with a Four Star Quality Rating and Statistical Analyses That Reported Using “Opportunity”

Mechanism ^**a**^	Total number of interventions using the C-M-O	Number of interventions using the C-M-O that report improvement in smoking cessation outcome
**CONTEXT**		
**NORTH AMERICA**		
**Opportunity**	**11** [51, 61, 63, 82, 83, 96, 103, 105, 119, 120, 126]	**8 (73%)** [51, 61, 63, 82, 83, 96, 103, 105]
**Opportunity – Access**	**7** [61, 63, 82, 83, 96, 103, 119]	**6 (86%)** [61, 63, 82, 83, 96, 103]
**Opportunity – Changing Physical and/or Social Environment**	**6** [61, 83, 96, 103, 105, 120]	**5 (83%)** [61, 83, 96, 103, 105]
**Opportunity – Social Support**	**6** [51, 82, 96, 103, 105, 119]	**5 (83%)** [51, 82, 96, 103, 105]
**ASIA**		
**Opportunity – Social Support**	**3** [65, 75, 125]	**2 (67%)** [65, 75]
**CLINICAL SETTING**		
**Opportunity – Access**	**3** [61, 82, 95]	**3 (100%)** [61, 82, 95]
**COMMUNITY BASED CARE**		
**Opportunity**	**5** [40, 61, 63, 92, 119]	**4 (80%)** [40, 61, 63, 92]
**Opportunity – Access**	**4** [61, 63, 92, 119]	**3 (75%)** [61, 63, 92]
**WORKPLACE**		
**Opportunity – Access**	**3** [96, 103, 121]	**2 (67%)** [96, 103]
**Opportunity – Social Support**	**3** [96, 103, 105]	**3 (100%)** [96, 103, 105]
**SCHOOLS**		
**Opportunity**	**4** [40, 56, 71, 83]	**4 (100%)** [40, 56, 71, 83]
**Opportunity – Changing Physical and/or Social Environment**	**4** [40, 56, 71, 83]	**4 (100%)** [40, 56, 71, 83]
**TARGET POPULATION**		
**PRIMARY PREVENTION**		
**Opportunity**	**20** [40, 54–56, 61, 63, 71, 82, 83, 96, 101, 103, 105, 115, 117, 118, 120, 121, 124, 127]	**13 (65%)** [40, 54–56, 61, 63, 71, 82, 83, 96, 101, 103, 105]
**Opportunity – Access**	**8** [56, 61, 63, 82, 83, 96, 103, 121]	**7 (88%)** [56, 61, 63, 82, 83, 96, 103]
**Opportunity - Changing Physical and/or Social Environment**	**11** [40, 56, 61, 71, 83, 96, 103, 105, 118, 120, 121]	**8 (73%)** [40, 56, 61, 71, 83, 96, 103, 105]
**Opportunity – Social Support**	**9** [54, 55, 82, 96, 101, 103, 105, 124, 127]	**7 (78%)** [54, 55, 82, 96, 101, 103, 105]
**SECONDARY PREVENTION**		
**Opportunity – Access**	**4** [49, 92, 95, 119]	**3 (75%)** [49, 92, 95]
**TYPE OF MODIFIABLE RISK BEHAVIOUR INTERVENTION ADDRESSED**^**b**^		
**ALCOHOL**		
**Opportunity**	**13** [49, 51, 75, 82, 83, 101, 103, 105, 115, 117–119, 122]	**8 (62%)** [49, 51, 75, 82, 83, 101, 103, 105]
**Opportunity – Access**	**5** [49, 82, 83, 103, 119]	**4 (80%)** [49, 82, 83, 103]
**Opportunity – Changing Physical and/or Social Environment**	**4** [83, 103, 105, 118]	**3 (75%)** [83, 103, 105]
**Opportunity – Social Support**	**7** [51, 75, 82, 101, 103, 105, 119]	**6 (86%)** [51, 75, 82, 101, 103, 105]
**DIET**		
**Opportunity – Access**	**11** [56, 61, 63, 82, 83, 92, 95, 96, 103, 119, 121]	**9 (82%)** [56, 61, 63, 82, 83, 92, 95, 96, 103]
**Opportunity – Changing Physical and/or Social Environment**	**11** [40, 56, 61, 71, 83, 96, 103, 105, 118, 120, 121]	**8 (73%)** [40, 56, 61, 71, 83, 96, 103, 105]
**Opportunity – Social Support**	**14** [54, 55, 65, 75, 82, 96, 101, 103, 105, 119, 123–125, 127]	**9 (64%)** [54, 55, 65, 75, 82, 96, 101, 103, 105]
**PHYSICAL ACTIVITY**		
**Opportunity – Access**	**12** [49, 56, 61, 63, 82, 83, 92, 95, 96, 103, 119, 121]	**10 (83%)** [49, 56, 61, 63, 82, 83, 92, 95, 96, 103]
**Opportunity – Changing Physical and/or Social Environment**	**11** [40, 56, 61, 71, 83, 96, 103, 105, 118, 120, 121]	**8 (73%)** [40, 56, 61, 71, 83, 96, 103, 105]
**Opportunity – Social Support**	**15** [51, 54, 55, 65, 75, 82, 96, 101, 103, 105, 119, 123–125, 127]	**10 (67%)** [51, 54, 55, 65, 75, 82, 96, 101, 103, 105]
**STRESS**		
**Opportunity**	**10** [40, 92, 95, 96, 101, 103, 105, 118, 119, 126]	**7 (70%)** [40, 92, 95, 96, 101, 103, 105]
**Opportunity – Access**	**5** [92, 95, 96, 103, 119]	**4 (80%)** [92, 95, 96, 103]
**Opportunity – Changing Physical and/or Social Environment**	**5** [40, 96, 103, 105, 118]	**4 (80%)** [40, 96, 103, 105]
**Opportunity – Social Support**	**5** [96, 101, 103, 105, 119]	**4 (80%)** [96, 101, 103, 105]
**NUMBER OF MODIFIABLE RISK BEHAVIOURS**		
**3 BEHAVIOURS**		
**Opportunity – Access**	**5** [49, 56, 61, 63, 121]	**4 (80%)** [49, 56, 61, 63]
**Opportunity – Changing Physical and/or Social Environment**	**5** [56, 61, 71, 120, 121]	**3 (60%)** [56, 61, 71]
**4 BEHAVIOURS**		
**Opportunity**	**11** [40, 75, 82, 83, 92, 95, 96, 115, 117, 122, 126]	**7 (64%)** [40, 75, 82, 83, 92, 95, 96]
**Opportunity – Access**	**5** [82, 83, 92, 95, 96]	**5 (100%)** [82, 83, 92, 95, 96]
**Opportunity – Changing Physical and/or Social Environment**	**3** [40, 83, 96]	**3 (100%)** [40, 83, 96]
**Opportunity – Social Support**	**3** [75, 82, 96]	**3 (100%)** [75, 82, 96]
**5 BEHAVIOURS**		
**Opportunity – Social Support**	**3** [101, 103, 119]	**2 (67%)** [101, 103]

^a^ The mechanisms within these interventions may not be exclusively for addressing smoking behaviour. It could be part of the overall intervention or for other risk behaviours within that intervention

^b^ This category examines interventions that include the specific risk behaviour (i.e. alcohol) as one of the targeted behaviours

### Demi-regularity – capability

For this review, capability was defined as the “individual’s psychological and physical capacity to engage in healthy behaviours” [30]. The success of interventions that included capability as a mechanism appears to be dependent on various factors, including: the specific context in which these interventions were implemented, the populations that were targeted, and the types of behaviours targeted in the intervention. When examining specific techniques for increasing capability, including “capacity to plan”, “enhancing knowledge” and “empowerment”, the effectiveness of these techniques was dependent on the context in which it was implemented.

#### Supporting evidence

Of the 53 interventions in our sample that were based on this mechanism [12, 40, 43, 49, 51, 52, 54–56, 61, 63, 65, 69, 71, 75, 81–83, 92, 95, 96, 98, 101, 103, 105, 115–142], only 23 (43%) interventions resulted in long-term smoking cessation [40, 49, 51, 52, 54–56, 61, 63, 65, 69, 71, 75, 81–83, 92, 95, 96, 98, 101, 103, 105]. Unlike the trends observed with opportunity, the majority of interventions that used capability as one of the mechanisms were not successful. These trends persisted when we looked at specific techniques for increasing capability. Only one [75] of the four interventions [75, 123, 130, 131] that sought to change to one’s beliefs about the intervention (a technique used to increase capability) reported successful long-term smoking cessation.

However, there were certain contexts in which interventions based on this mechanism observed more success. Specifically, four [56, 65, 69, 75, 81] out of eight interventions [56, 65, 69, 75, 81, 118, 122, 125] in Asia reported that participants were more likely to quit smoking. Interventions that utilized capability in community based settings or in schools also had positive results with 71% (5/7) [40, 61, 63, 81, 92] and 80% (4/5) [40, 56, 71, 83], respectively reporting long-term smoking cessation.

There was only one observable trend among interventions that used capability to target primary prevention or secondary prevention**.** Interventions that targeted secondary prevention and aimed to empower participants did not appear to be effective. Only two (40%) [65, 75] of the five interventions [65, 75, 133, 135, 140] reported successful long-term smoking cessation.

Conversely, there were certain contexts in which using capability as a mechanism appeared to negatively impact the success of the intervention. In Europe (6/19; 32%) [40, 52, 54, 55, 92, 95] and in Australasia (2/5; 40%) [49, 101], only a minority of the interventions using capability reported participants were more likely to quit smoking. Even when we examined specific techniques for increasing capability that were used in Europe, very few interventions reported successful long-term smoking cessation. Similar trends were observed with interventions that took place in clinical settings; only 37% (10/27) [40, 52, 55, 61, 65, 69, 75, 81, 82, 95] of the interventions reported participants were more likely to quit smoking.

The type and number of risk behaviours targeted by interventions that aimed to increase patient’s capability were also examined (Table 4). Unlike what was observed for opportunity, using capability as a mechanism in an intervention only appeared to be successful for certain behaviours and when specific techniques for increasing capability were used. For example, among interventions that targeted stress, the majority of interventions (6/10; 60%) [40, 95, 96, 101, 103, 105] that used “capacity to plan” demonstrated success in achieving long-term smoking cessation. Furthermore, 60% (3/5) [75, 101, 103] of interventions that targeted alcohol and used ‘empowerment’ as a technique for increasing capability reported long-term smoking cessation. However, only 33% (1/3) of interventions [75] that targeted alcohol and used the technique of changing ‘beliefs about the intervention’ reported long-term smoking cessation.

**Table 4:** Interventions with a Four Star Quality Rating and Statistical Analyses That Reported Using “Capability”

Mechanism ^**a**^	Total number of interventions using the C-M-O	Number of interventions using the C-M-O that report improvement in smoking cessation outcome
**CONTEXT**		
**NORTH AMERICA**		
**Capability - Empowerment**	**3** [103, 138, 140]	**1 (33%)** [103]
**EUROPE**		
**Capability**	**19** [40, 52, 54, 55, 92, 95, 115–117, 123, 124, 127, 129–133, 135, 136]	**6 (32%)** [40, 52, 54, 55, 92, 95]
**Capability - Beliefs about Interventions**	**3** [123, 130, 131]	**0 (0%)**
**Capability- Capacity to Plan**	**14** [40, 52, 54, 55, 95, 115, 116, 123, 124, 127, 129, 131, 133, 136]	**5 (36%)** [40, 52, 54, 55, 95]
**Capability- Empowerment**	**6** [54, 124, 131, 133, 135, 136]	**1 (17%)** [54]
**Capability - Enhance Knowledge and Skills of Individual**	**18** [40, 52, 54, 55, 92, 95, 115–117, 124, 127, 129–133, 135, 136]	**6 (33%)** [40, 52, 54, 55, 92, 95]
**ASIA**		
**Capability**	**8** [56, 65, 69, 75, 81, 118, 122, 125]	**5 (63%)** [56, 65, 69, 75, 81]
**Capability - Capacity to Plan**	**5** [69, 75, 81, 122, 125]	**3 (60%)** [69, 75, 81]
**Capability - Enhance Knowledge and Skills of Individual**	**8** [56, 65, 69, 75, 81, 118, 122, 125]	**5 (63%)** [56, 65, 69, 75, 81]
**AUSTRALASIA**		
**Capability**	**5** [12, 49, 101, 128, 139]	**2 (40%)** [49, 101]
**Capability - Capacity to Plan**	**4** [12, 101, 128, 139]	**1 (25%)** [101]
**COMMUNITY BASED CLINICAL SETTING**		
**Capability**	**27** [12, 40, 43, 52, 55, 61, 65, 69, 75, 81, 82, 95, 116, 117, 123–127, 130, 132–136, 140, 141]	**10 (37%)** [40, 52, 55, 61, 65, 69, 75, 81, 82, 95]
**Capability – Beliefs about Intervention**	**3** [75, 123, 130]	**1 (33%)** [75]
**Capability – Capacity to Plan**	**20** [12, 40, 43, 52, 55, 69, 75, 81, 82, 95, 116, 123–125, 127, 133, 134, 136, 140, 141]	**8 (40%)** [40, 52, 55, 69, 75, 81, 82, 95]
**Capability - Empowerment**	**7** [65, 75, 124, 133, 135, 136, 140]	**2 (29%)** [65, 75]
**COMMUNITY BASED CARE**		
**Capability**	**7** [40, 61, 63, 81, 92, 119, 141]	**5 (71%)** [40, 61, 63, 81, 92]
**Capability - Capacity to Plan**	**5** [40, 63, 81, 119, 141]	**3 (60%)** [40, 63, 81]
**Capability - Enhance Knowledge and Skills of individual**	**6** [40, 61, 63, 81, 92, 119]	**5 (83%)** [40, 61, 63, 81, 92]
**WORKPLACE**		
**Capability - Capacity to Plan**	**4** [96, 103, 105, 138]	**3 (75%)** [96, 103, 105]
**SCHOOLS**		
**Capability**	**5** [40, 56, 71, 83, 131]	**4 (80%)** [40, 56, 71, 83]
**Capability – Capacity to Plan**	**3** [40, 83, 131]	**2 (67%)** [40, 83]
**Capability – Enhance Knowledge and Skills of Individual**	**5** [40, 56, 71, 83, 131]	**4 (80%)** [40, 56, 71, 83]
**TARGET POPULATION**		
**SECONDARY PREVENTION**		
**Capability – Capacity to Plan**	**15** [51, 55, 69, 75, 81, 95, 119, 122, 125, 127–129, 133, 134, 140]	**6 (40%)** [51, 55, 69, 75, 81, 95]
**Capability – Empowerment**	**5** [65, 75, 133, 135, 140]	**2 (40%)** [65, 75]
**PROFESSION**		
**MULTI-DISCIPLINARY TEAM**		
**Capability – Capacity to Plan**	**10** [40, 51, 55, 63, 69, 81, 82, 116, 124, 140]	**7 (70%)** [40, 51, 55, 63, 69, 81, 82]
**Capability – Enhance Knowledge and Skills of Individual**	**13** [40, 51, 55, 63, 69, 81, 82, 92, 116, 120, 121, 124, 130]	**8 (62%)** [40, 51, 55, 63, 69, 81, 82, 92]
**TYPE OF MODIFIABLE RISK BEHAVIOURS INTERVENTION ADDRESSED**^**b**^		
**ALCOHOL**		
**Capability - Beliefs about Interventions**	**3** [75, 130, 131]	**1 (33%)** [75]
**Capability - Empowerment**	**5** [75, 101, 103, 131, 135]	**3 (60%)** [75, 101, 103]
**DIET**		
**Capability - Beliefs about Interventions**	**4** [75, 123, 130, 131]	**1 (25%)** [75]
**PHYSICAL ACTIVITY**		
**Capability - Beliefs about Interventions**	**4** [75, 123, 130, 131]	**1 (25%)** [75]
**STRESS**		
**Capability - Capacity to Plan**	**10** [40, 95, 96, 101, 103, 105, 119, 128, 137, 138]	**6 (60%)** [40, 95, 96, 101, 103, 105]
**Capability - Empowerment**	**3** [101, 103, 138]	**2 (67%)** [101, 103]
**NUMBER OF MODIFIABLE RISK BEHAVIOURS**		
**3 BEHAVIOURS**		
**Capability - Capacity to Plan**	**19** [12, 51, 52, 54, 55, 63, 69, 116, 123–125, 127, 129, 133, 134, 136, 137, 140, 141]	**6 (32%)** [51, 52, 54, 55, 63, 69]
**Capability - Empowerment**	**7** [54, 65, 124, 133, 135, 136, 140]	**2 (29%)** [54, 65]
**4 BEHAVIOURS**		
**Capability – Beliefs about Intervention**	**3** [75, 130, 131]	**1 (33%)** [75]
**5 BEHAVIOURS**		
**Capability**	**7** [101, 103, 118, 119, 128, 132, 138]	**2 (29%)** [101, 103]
**Capability – Capacity to Plan**	**5** [101, 103, 119, 128, 138]	**2 (40%)** [101, 103]
**Capability – Empowerment**	**3** [101, 103, 138]	**2 (67%)** [101, 103]
**Capability - Enhance Knowledge and Skills of Individual**	**6** [101, 118, 119, 128, 132, 138]	**1 (17%)** [101]

^a^ The mechanisms within these interventions may not be exclusively for addressing smoking behaviour. It could be part of the overall intervention or for other risk behaviours within that intervention

^b^ This category examines interventions that include the specific risk behaviour (i.e. alcohol) as one of the targeted behaviours

### Demi-regularity – motivation

Motivation, defined as “all those brain processes that energize and direct behaviour, not just goals and conscious decision-making” [30], appears to be effective in certain contexts for improving smoking cessation outcomes.

#### Supporting evidence

Thirty seven interventions [12, 40, 43, 52, 55, 56, 58, 63, 69, 77, 82, 92, 95, 96, 98, 101, 103, 105, 115, 117, 118, 120, 123–125, 127, 130, 131, 133, 134, 136–138, 140–143] in our sample utilized motivation as a mechanism. However, based on our criteria, there were very few techniques involving motivation that had any reportable trends. As a result, we have chosen to provide a descriptive overview of these trends.

Interventions that aimed to increase participant’s motivation had mixed results, as 43% (16/37) [40, 52, 55, 56, 58, 63, 69, 77, 82, 92, 95, 96, 98, 101, 103, 105] of studies reported an association between increasing participant’s motivation and long-term smoking cessation. Similar to capability, implementing strategies that increased motivation within interventions appeared to be beneficial in certain contexts. For example, the majority of interventions that utilized motivation in community-based settings (3/4; 75%) [40, 63, 92] and schools (2/3; 67%) [40, 56] reported that participants were more likely to quit smoking long-term. In contrast, applying motivation as a mechanism was unsuccessful in clinical settings; eight (36%) [40, 52, 55, 58, 69, 77, 82, 95] of the 22 [12, 40, 43, 52, 55, 58, 69, 77, 82, 95, 117, 123–125, 127, 130, 133, 134, 136, 140, 141, 143] interventions in these settings reported participants were more likely to quit smoking.

In terms of the number of behaviours that were targeted, interventions targeting three behaviours demonstrated limited success; only six (32%) [52, 55, 56, 58, 63, 69] of the 19 interventions [12, 52, 55, 56, 58, 63, 69, 120, 123–125, 127, 133, 134, 136, 137, 140, 141, 143] reported long-term smoking cessation. The types of behaviours targeted by these interventions were also examined. The majority of interventions that focused on increasing patient’s motivation and targeted stress (8/12; 67%) [40, 92, 95, 96, 98, 101, 103, 105] reported a greater likelihood of long-term smoking cessation.

## Discussion

Health behaviour change programs that address multiple behaviours have been the subject of much discussion because there are a multitude of ways in which these programs can be developed, including: the types and numbers of modifiable risk behaviours to target [144], the types of activities to use [30], and the types of professions to involve [145–147]. In the current literature, there is a clear gap in the understanding of why some interventions have worked and others have not [46]. The results of this rapid realist review represent the first step in addressing this gap. Specifically, the goal of this paper was to review published interventions targeting multiple modifiable risk behaviours to uncover demi-regularities that contribute to the success of the program in helping people quit smoking. In other words, what contexts and mechanisms (i.e. structures, activities or processes within interventions that contribute to the outcomes of interest) lead to long-term smoking cessation.

The results of this rapid realist review emphasize the importance of incorporating mechanisms that modify external factors in multiple health behaviour interventions that attempt to achieve long-term smoking cessation. Specifically, interventions that made resources (e.g. pharmacotherapy, exercise, healthy foods) more accessible, changed the physical environment (e.g. introduced smoke free polices), or increased one’s social support network, were more likely to help individuals quit smoking. Evidence to support these findings were noticeable across different regions, settings, and behaviours. These findings challenge individualistic epidemiology that many health promotion interventions are based on, namely that health behaviours are a matter of individual choice [148]. While individuals do have ‘free choice’, research shows that the environment can significantly influence the decisions they make [149–151].

The findings from this rapid realist review also show that the success of interventions targeting motivation and capability appears to be dependent on the context. For example, interventions in Asia that tried to increase capability were usually successful in helping people quit smoking, while European interventions were not. The literature shows that while smoking is no longer socially acceptable in North America and Europe [152, 153], for many countries in Asia (e.g. China), smoking continues to be an accepted social activity [154, 155]. Thus, it could be hypothesized that interventions that increase person’s psychological capabilities (such as enhancing knowledge and skills) are more successful in contexts where the social climate was favourable to smoking. This finding is similar to previous research, which shows that the impact of knowledge on promoting successful behaviour change is dependent on the context in which it is provided [156, 157].

With regards to the trends we have observed with interventions that target motivation, the majority of these interventions did not appear to be successful in clinical settings but were successful in schools and community-based settings. A possible explanation for this is interventions delivered in non-clinic settings are reaching populations that are not motivated as they are not currently seeking out health care. Populations that are already in health care settings or seeking health care are likely to be intrinsically motivated to make changes to their behaviours [24, 158–163]. Thus, it is likely only in settings where motivation is low that having programs focusing on increasing motivation made a difference.

Significant strides have been made in the field of tobacco control to reduce the prevalence of smoking globally [164–166]. Tobacco control efforts have focused on changing the environment and the opportunities available to individuals (e.g. legislation introducing smoke-free places, warning-labels on tobacco products) [164–166], which coincides with the demi-regularities we have found in our review. However, for many other modifiable risk behaviours including alcohol and physical inactivity, public health promotion has traditionally relied on education, information, and psychosocial interventions to persuade individuals to adopt healthy lifestyles [167, 168]. Moreover, many widely used theories for behaviour change, including the Theory of Reasoned Action, Theory of Planned Behaviour, and the Trans-theoretical Model of Behaviour Change have focused primarily on the individual [169–171]. These theories do not account for the influence of environmental factors and thus may further contribute to the individualistic approach in addressing these modifiable risk behaviours. While some studies (*n* = 52 [38%]) in this review report using an established theory (e.g. Trans-theoretical Model of Behaviour Change), the majority did not report whether they used a theoretical framework (*n* = 86 [62%]). Thus we were unable to examine how the application of these theoretical frameworks within the interventions may have influenced the results of this study. In the current population of smokers, who engage in additional modifiable risk behaviours, this review shows that it is important for interventions to focus on changing an individual’s opportunity to adopt additional healthy behaviours.

### Applying the COM-B model

Using the COM-B model as our framework to categorize the mechanisms provided a systematic and standardized method to code the diverse interventions [172, 173]. This method is aligned with other reviews examining effective behaviour change interventions [174, 175]. It also allowed us to use clearly-defined techniques that maps on to the COM-B model, and a behaviour change taxonomy; thereby ensuring transparency and enabling replication [30, 31]. Focusing our analysis of mechanisms using the COM-B model also allowed for documentation of internal and external drivers of behaviour change, something many other theories do not allow [169–171]. In addition, we were able to develop a high level of understanding of the general trends that influence whether a program that targets multiple health behaviors is effective to help people quit smoking. Therefore, using the COM-B model provided the first step in understanding the determinants of successful behaviour change. While the COM-B model has been criticized for ignoring contextual characteristics [176], this argument cannot be made for this rapid realist review as we also analyzed context as part of the C-M-O configuration. The COM-B model was used only to code the mechanisms and we subsequently examined the contexts which these mechanisms were operationalized.

There were some limitations to using the COM-B model as the framework for the mechanisms in the C-M-O configuration. As mentioned by other researchers [176], this model in its current form does not provide specific and tangible actions to incorporate into the design of an intervention. However, the COM-B model can be mapped onto the Behaviour Change Wheel [30], which provide specific recommendations for intervention designs that can be used to address each component of the COM-B. Thus, findings from this study are not intended to be final; rather they offer guidance on the next steps in generating hypotheses for future intervention studies. The COM-B model provides directions on the mechanisms and consequently, the areas of the Behaviour Change Wheel that should be explored further.

Also important to note is that Michie’s group is currently developing and mapping Mechanisms of Action (MoA) to behaviour change techniques [177]. MoAs are defined as “the process through which behaviour change occurs” [177]. In the future, it might become useful to apply the findings of this review to the MoA. Carey et al.’s study [178], which was published after we had started coding our work, has hypothesised some possible links between BCTs and MoA. The MoAs expressed in Carey et al. are more granular than the ones we used in this study. However, since it is based on the Behaviour Change Technique Taxonomy we should be able to draw comparisons. For example, some of the common MoAs identified by Carey et al. include: knowledge, skills, behavioural regulations, attitudes towards the behaviour, beliefs about capability, and beliefs about consequences. These are all characteristics we identified and coded in each study and then grouped as “Capability” using the COM-B model’s definition.

### Limitations

A major limitation of this review is that many of the articles reviewed did not describe the context or the behavioural change techniques used in detail. For example, we were unable to determine whether the setting in which an intervention took place already had other resources and supports that contributed to the success of the intervention (e.g. highly experienced staff). As a result, the contexts we were able to examine were broad, including: the continent in which the intervention took place and the type of setting (e.g. clinical, workplace). Given the wide variety in the types of populations that interventions targeted, we were not able to examine trends by target population.

Moreover, in some instances, the studies did not provide sufficient details on the types of activities undertaken as a part of the intervention. This under-reporting of active ingredients was recently discussed in a systematic review and meta-analysis of smoking cessation interventions [172, 173, 179]. Bruin et al. (2019) described the challenge of reporting all the BCTs in a complex intervention as they can include 20 or more BCTs [179]. It has been shown that even a routine visit between patients and health care professionals can include 15 or more BCTs [180–182].

It is important to also acknowledge that there were several challenges associated with isolating the mechanisms (i.e. capability, opportunity, motivation) within the interventions. Specifically, it was often difficult to determine which mechanisms were targeted in studies that provided insufficient information on study design, and/or implementation. To get around this challenge, we reviewed articles with four star quality ratings first to determine the demi-regularities. However, these limitations also meant that, in some situations, we were unable to identify whether a specific mechanism was used. This may further contribute to the under-reporting of the mechanisms targeted within the interventions.

Lastly, the majority of interventions in this review targeted at least two mechanisms simultaneously (*n* = 97, 70%). It is unclear the degree of overlap between mechanisms and how these mechanisms interact to produce the observed outcome in these interventions. As a result, there may have been additional mechanisms and techniques that were employed by these interventions that we were unable to capture in our analyses. Future areas of research should also include examining the order in which behaviours should be addressed, (simultaneously vs sequential), the combination of behaviours to target, and the combination of mechanisms to target.

## Conclusion

Our study is the first to apply a realist review methodology to explore how, why and under what circumstances programs that target multiple risk behaviours are associated with long-term smoking cessation. It offers an explanatory view of the existing and extensive body of literature on interventions targeting multiple health behaviours. The evidence we reviewed came from a variety of countries, continents, settings, and targeted different behaviours, and yet we were able to observe clear and consistent themes regarding the importance of increasing an individual’s opportunity in multiple health behaviour change interventions. With regards to Picking Up the PACE, the results of this rapid realist review informed the development of courses for practitioners on how to support individuals who have multiple risk behaviours and are trying to quit smoking. Moving forward, programs that aim to address multiple modifiable risk behaviours as a part of a smoking cessation intervention should strongly consider incorporating activities that target external factors (i.e. social support, and access to treatment). The findings of the review highlight the value and importance of the COM-B model for effective behaviour change. We recommend that decision-makers, policy makers and implementers who are aiming to design multiple health behaviour change interventions should consistently apply the COM-B model, take context into consideration, and wherever feasible, include activities that improve an individual’s opportunities to make successful behaviour change.

## Supplementary information

**Additional file 1.** Search Strategy for Medline.

**Additional file 2.** Quality Assessment and Data Extraction Form.

**Additional file 3. Table 5:** All Interventions That Reported Using Opportunity as one of the Mechanisms. **Table 6:** All Interventions That Reported Using Capability as one of the Mechanisms. **Table 7:** All Interventions That Reported Using Motivation as One of the Mechanisms.

## Data Availability

The datasets used and/or analyzed during the current study are available from the corresponding author on reasonable request.

## Abbreviations

C-M-O: Context, Mechanism, Outcome
COM-B: Capability (C), opportunity (O), motivation (M) - behaviour (B)
PACE: Promoting and Accelerating Change through Empowerment
