# Supplementary material for: Identifying contexts and mechanisms in multiple behavior change interventions affecting smoking cessation success: a rapid realist review
Source: BMC Public Health. 2020 Jun 12;20:918. doi: 10.1186/s12889-020-08973-2 (PMC7291527; doi:10.1186/s12889-020-08973-2)
Supplement: Supplementary file 1 — Additional file 1. Search Strategy for Medline. [file 12889_2020_8973_MOESM1_ESM.pdf]

Search Strategy for Medline

NB: Search strategies for the other databases were similar in structure, and used equivalent search terms and the same synonyms. Contact the corresponding author at terri.rodak@camh.ca for the full search strategy for each database.

1 "Tobacco Use Disorder"/
2 "Tobacco Use"/
3 exp Smoking/
4 ((Cigar* or tobacco or nicotine) adj5 (smoke* or smoking or user* or addict* or depend*)).ti,ab,kf,hw.
5 smoking.ti,ab,kf,hw.
6 smoking cessation/
7 ((Cigar* or tobacco or nicotine) adj5 (cessation or quit* or abstain* or abstin* or reduc*)).ti,ab,kf,hw.
8 or/1-7 [smoking terms]

9 (multipl* or several or "more than two" or additional or integrat* or combin* or concurrent or cooccur* or co-occur* or multifactorial or multi-factorial).ti,ab,kf,hw.
10 Health Behavior/
11 risk reduction behavior/
12 exp health promotion/
13 life style/
14 (health behavio?r* or health risk behavio?r*).ti,ab,kf,hw.
15 or/10-14 [health behaviour terms]
16 9 and 15 ["multiple" terms + health behaviour terms]
17 ((multipl* or several or "more than two" or additional or integrat* or combin* or concurrent or cooccur* or co-occur* or multifactorial or multi-factorial) adj3 ((health* or risk* or lifestyle* or "life style*") adj3 (behavio?r* or factor*))).ti,ab,kf,hw.
18 ((multipl* or several or "more than two" or addition* or integrat* or combin* or concurrent or cooccur* or co-occur* or multifactorial or multi-factorial) adj3 ((behavio?r* or lifestyle* or "life style*") adj3 (change* or modif*))).ti,ab,kf,hw.
19 or/16-18 [all multiple health behaviour terms]

20 exp alcohol drinking/
21 exp alcohol-related disorders/
22 exp alcoholic intoxication/
23 exp alcoholic beverages/
24 exp drinking behavior/
25 Alcohol*.ti,ab,kf,hw.
26 (Alcohol* adj3 (drink* or consum* or abus* or intake)).ti,ab,kf,hw.
27 "alcohol use".ti,ab,kf,hw.
28 ((Heavy or hazardous or binge or excess*) adj3 (drink* or alcohol*)).ti,ab,kf,hw.
29 (Alcohol* adj3 (abstain* or abstinen* or detox*)).ti,ab,kf,hw.
30 (beer or wine or cider or spirits or liquor).ti,ab,kf,hw.
31 or/20-30 [all alcohol terms]

32 exp Exercise/
33 exercise therapy/
34 physical fitness/
35 (Physical* adj3 (activ* or inactiv* or fit or fitness or endur*)).ti,ab,kf,hw. (119001)
36 Exerci*.ti,ab,kf,hw.
37 (sport* or walk* or run* or jog* or bike* or biking or bicycl* or swim* or aerobic*).ti,ab,kf,hw.
38 Sedentar*.ti,ab,kf,hw.
39 or/32-38 [all physical activity terms]

40 exp Diet/
41 Food habits/
42 feeding behavior/
43 food preferences/
44 nutrition therapy/
45 diet therapy/
46 Nutritio*.ti,ab,kf,hw.
47 (Health* adj3 (food* or diet* or eat*)).ti,ab,kf,hw.
48 (Unhealth* adj3 (food* or diet* or eat*)).ti,ab,kf,hw.
49 (Fruit* or Vegetable*).ti,ab,kf,hw.

50 ((food* or fat or fats or calori* or dietary) adj2 (intake or consum*)).ti,ab,kf,hw.
51 (portion* adj size*).ti,ab,kf,hw.
52 or/40-51 [all healthy eating terms]

53 Stress, psychological/
54 Relaxation therapy/
55 ((emotion* or life or psychologic*) adj3 (stress* or suffer*)).ti,ab,kf,hw.
56 (Stress* adj3 manag*).ti,ab,kf,hw.
57 Relax*.ti,ab,kf,hw.
58 Mindful*.ti,ab,kf,hw.
59 Meditat*.ti,ab,kf,hw.
60 53 or 54 or 55 or 56 or 57 or 58 or 59
61 (((posttraumatic or post-traumatic) adj1 stress*) or ptsd).ti,ab,kf,hw.
62 60 not 61
63 stress test*.ti,ab,kf,hw.
64 62 not 63 [stress terms]

65 exp Sleep/
66 exp Sleep Wake Disorders/
67 sleep*.ti,ab,kf,hw.
68 insomnia*.ti,ab,kf,hw.
69 or/65-68 [sleep terms]

70 (program* or intervention* or treat* or modalit* or trial*).ti,ab,kf,hw.
71 dt.fs.
72 rh.fs.
73 th.fs.
74 pc.fs.
75 or/70-74 [intervention terms]

76 8 and 75 [smoking and intervention]
77 76 and 19 [smoking + MHB + intervention]
78 76 and 31 and 39 [smoking + alcohol + exercise + intervention]

79 76 and 31 and 52 [smoking + alcohol + nutrition + intervention]

80 76 and 31 and 64 [smoking + alcohol + stress + intervention]

81 76 and 31 and 69 [smoking + alcohol + sleep + intervention]

82 76 and 39 and 52 [smoking + exercise + nutrition + intervention]

83 76 and 39 and 64 [smoking + exercise + stress + intervention]

84 76 and 39 and 69 [smoking + exercise + sleep + intervention]

85 76 and 52 and 64 [smoking + nutrition + stress + intervention]

86 76 and 52 and 69 [smoking + nutrition + sleep + intervention]

87 76 and 64 and 69 [smoking + stress + sleep + intervention]

88 or/77-87 [all health behaviour intervention combinations]

89 (editorial or letter).pt.

90 88 not 89

91 limit 90 to yr="2005 -Current"

92 limit 91 to english language
