# Supplementary material for: Identifying contexts and mechanisms in multiple behavior change interventions affecting smoking cessation success: a rapid realist review
Source: BMC Public Health. 2020 Jun 12;20:918. doi: 10.1186/s12889-020-08973-2 (PMC7291527; doi:10.1186/s12889-020-08973-2)
Supplement: Supplementary file 2 — Additional file 2. Quality Assessment and Data Extraction Form. [file 12889_2020_8973_MOESM2_ESM.docx]

## Additional File 2: Quality Assessment and Data Extraction Form

## What factors are associated with effective multiple health behavior change (three or more, including smoking cessation)?

**Quality Assessment and Data Extraction Form for Full Articles:**

| **Question** | **Options** | **Definitions/Additional notes** |
| --- | --- | --- |
| **Relevance Verification** | | |
| What language is the article in? | - English - Other (exclude) |  |
| Does this citation describe **interventions** that change **tobacco use** as well as **two or more** additional unhealthy **behaviors** (multiple health behavior change)?  *behavior change outcomes must be reported | - Yes – relevant research - Yes – relevant research but does not include outcomes on 3 or more behaviour changes (excluded, submit form) - No - not relevant (excluded, submit form) - No – but may be useful for further reading (excluded, submit form) - No – but check that we have included the following article: [text] (excluded, submit form) | **Interventions:** a program or strategy designed to produce behavior changes or improve health among individuals or populations. This can include educational programs, policies, health promotion campaigns etc.  **Behaviors:** nutrition, alcohol consumption, physical activity, stress and mood, and sleep |
| Specify all **behaviors** that this intervention aims to change (**2 or more in addition to smoking cessation).** | - Smoking cessation - Alcohol consumption - Nutrition - Physical activity/physical inactivity - Sleep - Stress and mood | **Behaviors:** including nutrition, alcohol consumption, physical activity, stress and mood, and sleep  ***MUST SELECT 2 OR MORE IN ADDITION TO SMOKING CESSATION** |
| **If exclusion criteria were selected above, submit the form before proceeding** | | |
| **Data Extraction** | | |
| **General Information** | | |
| What year was the article published? | [text] | **Year** e.g. 1980 |
| What type of document is this article? | - Primary research - Thesis - Conference proceeding - Literature review - Systematic review/meta-analyses - Book - Grey literature | **Primary research:** original research/investigation/study carried out by the researcher (includes surveys, interviews, outbreak reports, observations etc.)  **Thesis:** a long paper/essay or dissertation involving personal research (usually written for a university degree)  **Conference proceeding abstract/short paper:** an individual or collection of published academic papers  **Literature review:** examination of published literature  **Systematic review/meta-analyses:** analysis and interpretation of primary research  **Grey literature:** research that is unpublished or published in a non-commercial form (e.g. newspaper or magazine articles) |
| On what continent did the intervention take place?  (If not specified, resort to authour affiliations)  Specify the country, state or province. | - North America - Europe - Australasia - Central America/South America/Caribbean - Asia - Africa - Other: [text]   [text] | **North America:** includes Canada, USA and Mexico  **Europe:** includes, Belarus, Latvia, Ukraine, Estonia, Cyprus & west (includes Iceland and Greenland)  **Australasia:** limited to Australia, New Guinea, New Zealand, New Caledonia, and neighbouring islands, including the Indonesian islands from Lombok and Sulawesi eastward  **Central America/South America/ Caribbean:** includes Caribbean, and all of south and central America.  **Asia:** Russia, Turkey, middle eastern countries and east |
| What is the study design? | - Observational study - Cohort - Case-control - Other, specify: [text] - Experimental study - Randomized control trial - Quasi-experimental study - Other, specify: [text] - Qualitative study | **Cohort:** follow a group of exposed and non-exposed individuals to evaluate whether they develop an outcome  **Case control:** identified cases are matched with controls and their risk factors are evaluated for an association with outcome  **Randomized Control Trial:** individuals are allocated at random to a control or intervention group  **Quasi-experiment:** used to estimate the causal impact of an intervention on its target population without random assignment  **Qualitative:** primarily exploratory research. It is used to gain an understanding of underlying reasons, opinions, and motivations (e.g. focus groups, interviews etc.) |
| What theories were used to design the intervention? *(Check all that apply)* | - Behavioural Therapy - The Behaviour Change Approach - The Community Organization Approach - Social Learning Theory - Social Cognitive Theory - Social-Ecological Theory - Goal Systems Theory - Transtheoretical Model - Other: specify [text] - Not stated | **Behavioural Therapy:** structured approach which focuses on measuring what a person is doing and helps them understand how changing their behaviour can lead to positive experiences  **The Behaviour Change Approach:** process for planning and implementing a strategic set of interventions and activities to change behaviours  **The Community Organization Approach:** method of intervention where a professional change agent helps a community action system composed of individuals, groups, or organizations to engage in planned collective action to deal with a community issue  **Social Learning Theory:** proposes that new behaviours can be learned by observing and imitating others  **Social Cognitive Theory:** proposes that an individual’s behaviour is guided through observing others within the context of social interactions, experiences, and outside media influences  **Social-Ecological Theory:** framework for understanding the multifaceted and interactive effects of personal and environmental factors that determine behaviours  **Goal Systems Theory:** offers insight into achievement of goal dynamics through goals (representations of desired end-states) and goal-means (behaviours that can help one accomplish a goal)  **Transtheoretical Model:** assesses an individual’s readiness to act on a new behaviour, and provides strategies, or processes of change to guide the individual through the stages of change |
| How were the behaviour changes implemented? | - Simultaneously - Sequentially - Not stated | **Simultaneously:** The intervention attempted to change all behaviours at the same time  **Sequentially:** The intervention attempted to change the behaviours one after the other |
| Indicate the type of intervention. | - Primary prevention - Secondary prevention - Tertiary prevention | **Primary:** aims to prevent disease before it ever occurs. This is done by preventing exposures to hazards that cause disease, altering unhealthy behaviours that can lead to disease, and increasing resistance to disease should exposure occur (e.g. legislation, policy, or education).  **Secondary:** aims to reduce the impact of a disease that has already occurred. This is done by detecting and treating disease as soon as possible to halt or slow its progress, encouraging personal strategies to prevent recurrence, and implementing programs to return people to their original health and function to prevent long-term problems (e.g. regular exams and screening tests, diet modification counselling, or exercise programs).  **Tertiary:** aims to soften the impact of an ongoing illness that has lasting effects. This is done by helping people manage long-term, often complex health problems in order to improve as much as possible their ability to function, their quality of life, and their life expectancy (e.g. cardiac or stroke rehabilitation programs, chronic disease programs, or support groups). |
| Briefly describe the intervention | [text] |  |
| **Sample Population** | | |
| What is the sample size of participants in the study? (Specify for both intervention and control) | - Total [text] - Intervention [text] - Control [text] |  |
| Indicate the number of men and women in the study. (Specify for both intervention and control) | - Men [text] - Women [text] - Not stated |  |
| What was the target population of this intervention? *(Check all that apply)* | - General public - Pregnant women - Students, specify: [text] - Minority groups, specify: [text] - Disadvantaged, specify: [text] - Cardio patients, specify: [text] - Cancer patients, specify: [text] - Other, specify: [text] | Specify any relevant details about the target population |
| What age range are the participants of the study? (Specify for both intervention and control) | - Adolescents (<16) [text] - Adults (16-64) [text] - Elderly (65+) [text] |  |
| What is the race of the participants in the study? *(Specify for both intervention and control)* | - White/Caucasian [text] - Black or African American [text] - Aboriginal or Indigenous [text] - Asian/Pacific Islander [text] - Hispanic or Latino [text] - Other, specify: [text] - Not stated |  |
| Was any additional demographic information about the study participants captured? *(Specify for both intervention and control)* | - Socioeconomic status [text] - Occupation [text] - Education [text] - Culture/ethnicity [text] - Other, specify: [text] - Not stated |  |
| **Context** | | |
| Were any contextual factors discussed? | - Infrastructure [text] - Characteristics of region [text] - Social environment (prevailing norms, membership groups, culture) - Physical environment (financial resources, material resources, time pressures, location) - Other, specify: [text] - Not stated | **Infrastructure:** E.g. having an existing multi-disciplinary infrastructure (access to nurses, dietitians etc.) that the intervention can leverage  **Characteristics of region:** established evidence-base for severity of health outcomes in the region of intervention |
| **Activities** | | |
| What types of activities did the intervention use? *(Check all that apply)* | - Social support [text] - Delivery type:   - Face to face   - Over the phone   - Web   - App   - SMS text   - Mass media   - Other, specify: [text] - Counselling [text] - Delivery type:   - Face to face   - Over the phone   - Web   - App   - SMS text   - Mass media   - Other, specify: [text] - Motivational interviewing [text] - Delivery type:   - Face to face   - Over the phone   - Web   - App   - SMS txt   - Mass media   - Other, specify: [text] - Education [text] - Information about health consequences - Information about social and environmental consequences, - Information about emotional consequences - Information on avoidance/reducing exposure to cues for the behaviour - Instruction on how to perform/change a behaviour - Other, specify: [text] - Delivery type:   - Face to face   - Over the phone   - Web   - App   - SMS text   - Mass media   - Other, specify: [text] - Community/organization involvement [text] - Incentives [text] - Material rewards - Social rewards - Self-rewards - Tailored feedback - Biological feedback [text] - Self-monitoring of behaviour [text] - Delivery type:   - Face to face   - Over the phone   - Web   - App   - SMS text   - Mass media   - Other, specify: [text] - Opinion leaders [text] - Delivery type:   - Face to face   - Over the phone   - Web   - App   - SMS text   - Mass media   - Other, specify: [text] - Group interactions [text] - Delivery type:   - Face to face   - Over the phone   - Web   - Social Media   - Other, specify: [text] - Mass media campaigns [text] - Delivery type:   - TV   - Billboards   - Radio   - Movies   - Magazine/Newspaper   - Social media   - Other, specify: [text] - Pharmacological support [text] - Smoking - Alcohol - Stress/Mood/Depression - Sleep - Negative reinforcements [text] - Delivery type:   - Face to face   - Over the phone   - Web   - App   - SMS text   - Mass media   - Other, specify: [text] - Other, specify: [text] - Not stated | **Social support:** has assistance available from other people (friends, family etc). and that one is part of supportive social network  **Counselling:** provided advice or recommendations from a professional  **Motivational interviewing:** a psychotherapeutic approach that attempts to move an individual away from a state of indecision or uncertainty and towards finding motivation to make positive decisions.  **Education:** the process of giving or receiving information or instruction  **Community/organization involvement:** examples such as events, group challenges that involve the community or specific organization etc.  **Incentives:** rewards that motivate or encourage behavior change  **Tailored feedback:** specific individualized feedback based on participants experiences/goals/strengths etc. *Biological feedback* – providing results of a test and providing feedback. *Self-monitoring* – establish a method for the person to monitor and record their behavior(s) as part of a behavior change strategy *Scheduled consequences/negative reinforcements* – tailored to individual based on their behaviors.  **Opinion Leaders**: the use of opinion leaders or celebrities to motivate or encourage behavior change  **Group interactions:** using a group format or activity to motivate or encourage behavior change (e.g. group fitness class, AAA meeting)  **Mass media campaigns:** emails, social media, brochures etc used to promote behavior change  **Pharmacological support:** Provide, or encourage the use of or adherence to drugs to facilitate behavior change |
| **Mechanisms of Change** | | |
| Were any mechanisms of change identified or inferred? *(Check all that apply)* | - Capability - Enhance knowledge and skills of individual [text] - Capacity to plan [text] - Beliefs about intervention/treatment[text] - Empowerment [text] - Other, specify: [text] - Motivation - Increase motivation [text] - Inducing fear [text] - Incentivization [text] - Self-efficacy [text] - Other, specify: [text] - Opportunity - Changing physical and social environment to change social climate [text] - Improve feelings of support [text] - Cost [text] - Access [text] - Health care professional-participant relationship [text] - Other, specify: [text] - Not stated | Mechanisms can be described as “agents of change” and include the beliefs, values, desires, and cognitive or emotional reasoning of participants and stakeholders who receive or deliver interventions. They describe how the resources and activities embedded in a program influence the reasoning and ultimately the behaviour of program subjects  **Capability:** doing something that enables an individual to make a behaviour change. Physical and psychological capacity to engage in change  **Motivation:** doing something that encourages or persuades an individual to make a behaviour change  **Opportunity:** doing something that provides the freedom and context for an individual to make a behaviour change  Healthcare professional-participant relationship – the relationship between the patient and the person administering the intervention |
| **Outcomes** | | |
| Describe the impact of the intervention. | - Smoking cessation [text] - Alcohol consumption [text] - Nutrition [text] - Physical activity/inactivity [text] - Sleep [test] - Stress and mood [text] - Other biological impacts [text] | **Remember we are looking for behaviour changes  *Place all other biological impacts (i.e. cholesterol, triglycerides under biological impacts) |
| How were the outcomes measured? | [text] |  |
| Describe any facilitators or barriers to the success of the intervention/program | [text] | E.g. engagement of participants |
| **Quality Assessment** | | |
| What type of study was this? | - Qualitative (move to qualitative questions) - Quantitative (move to quantitative questions) - Mixed-Method (move to mixed-method questions) | **Qualitative:** research that gathers information that is not numerical (e.g. interviews, focus groups etc.)  **Quantitative:** research that gathers numerical data that can be put into categories, or in rank order, or measured in units of measurement  **Mixed-method:** research that involves the mixing of quantitative and qualitative methods (e.g. exploratory design) |
| **Qualitative Quality Assessment** | | |
| Was there a clear statement of the research purpose/aims? | - Yes [text] - No [text] - Can’t Tell [text] | Consider the following to make a judgement: clarity of focus, explicit purpose given, supported by prior research |
| Does the collected data address the research question (or objectives)? | - Yes [text] - No [text] - Can’t Tell [text] | Consider whether the research and data collection strategy clearly described and was appropriate to address the research or objectives |
| Are the sources of qualitative data relevant to address the research question (objective)? | - Yes [text] - No [text] - Can’t Tell [text] | *Was the recruitment strategy appropriate to the aims of the research?* Consider whether (a) the selection of the participants is clear and appropriate to collect relevant and rich data; and (b) reasons why certain potential participants were chosen, or chose not to participate |
| Is the process for analyzing qualitative data relevant to address the research question (objective)? | - Yes [text] - No [text] - Can’t Tell [text] | *Was the data collected in a way that addressed the research issue?* Consider whether (a) the method of data collection is clear (e.g. focus group, interview); (b) the form of the data is clear (e.g. tape recordings, video material); (c) changes are explained when methods are altered during the study; (d) the qualitative data analysis addresses the question; and (e) the researcher has discussed saturation of data |
| Is appropriate consideration given to how findings relate to the context, e.g., the setting in which the data were collected? | - Yes [text] - No [text] - Can’t Tell [text] | Consider whether the study context and how findings relate to the context and characteristics of the context are explained (how findings are influenced by or influence the context) |
| Is appropriate consideration given to how findings relate to researchers’ influence, e.g., through their interactions with participants? | - Yes [text] - No [text] - Can’t Tell [text] | *Has the relationship between researcher and participants been adequately considered?* Consider whether (a) researchers critically explain how findings relate to their perspective, role, and interactions with participants (how the research process is influenced); (b) researcher’s role is influential at all stages; and (c) researchers explain their reaction to critical events that occurred during the study |
| Was the data analysis sufficiently rigorous? | - Yes [text] - No [text] - Can’t Tell [text] | Consider whether (a) data provided sufficient depth, detail and richness? (e.g. illustrative quotes); (b) an in-depth description of the analysis process (c) context described and taken into account in interpretation/results; (d) approaches taken to ensure robustness (e.g. multiple analysts, triangulation, member checking/participant validation of results) |
| Have ethical issues been taken into consideration? | - Yes [text] - No [text] - Can’t Tell [text] | Consider whether (a) study was approved by ethics committee; (b) sufficient details provided on how the research was explained to participants and whether ethical standards were maintained; (c) documentation of how autonomy, consent, confidentiality, anonymity were managed; and (d) documentation of any ethical dilemmas and how they were resolved |
| Is there a clear statement of findings? | - Yes [text] - No [text] - Can’t Tell [text] | Consider whether (a) the findings are explicit; (b) adequate discussion of the evidence both for and against the researchers arguments; (c) researcher has discussed the credibility of their findings (e.g. triangulations, respondent validations); and (d) findings are discussed in relation to research question |
| Was there evidence of study relevance and transferability? | - Yes [text] - No [text] - Can’t Tell [text] | Consider whether (a) there is a discussion of contribution of study to existing/prior knowledge, practice, and/or policy; (b) areas for future research identified; (c) limitations/weaknesses of study are clearly outlined; and (d) there is a discussion of whether or how the findings can be transferred to other populations or consideration of other ways the research may be used |
| **Quantitative Quality Assessment** | | |
| Was the study a randomized control trial? | - Yes (moves to randomized control questions) - No (moves to non-randomized study questions) | **Randomized Control Trial:** individuals are allocated at random to a control or intervention group |
| *Randomized Control Trials* | | |
| Was there a clear statement of the research purpose/aims? | - Yes [text] - No [text] - Can’t Tell [text] | Consider the following to make a judgement: clarity of focus, explicit purpose given, supported by prior research |
| Does the collected data address the research question (or objectives)? | - Yes [text] - No [text] - Can’t Tell [text] | Consider whether the research and data collection strategy clearly described and was appropriate to address the research or objectives |
| Is there a clear description of the randomization (or appropriate sequence generation)? | - Yes [text] - No [text] - Can’t Tell [text] | Consider whether researchers describe how the randomization or allocation is generated. A simple statement such as “we randomly allocated” or “using a randomized design” does not suffice. |
| Is there a clear description of the allocation concealment (or blinding when applicable)? | - Yes [text] - No [text] - Can’t Tell [text] - Not applicable | Consider whether (a) researchers and participants were unaware of the assignment sequence up to the point of allocation; or (b) researchers and participants are unaware of the group a participant is allocated to during the course of the study |
| Were the groups similar at the start of the trial? | - Yes [text] - No [text] - Can’t Tell [text] | Consider other factors that might affect the outcome such as age, sex, social class etc. |
| Are there complete outcome data (80% or above)? | - Yes [text] - No [text] - Can’t Tell [text] | Almost all of the participants contributed to almost all measures |
| Is there low withdrawal/drop-out (below 20%)? | - Yes [text] - No [text] - Can’t Tell [text] | Almost all of the participants completed the study |
| Was there evidence of study relevance and transferability? | - Yes [text] - No [text] - Can’t Tell [text] | Consider whether (a) there is a discussion of contribution of study to existing/prior knowledge, practice, and/or policy; (b) areas for future research identified; (c) limitations/weaknesses of study are clearly outlined; and (d) there is a discussion of whether or how the findings can be transferred to other populations or consideration of other ways the research may be used |
| *Non-Randomized Studies* | | |
| Was there a clear statement of the research purpose/aims? | - Yes [text] - No [text] - Can’t Tell [text] | Consider the following to make a judgement: clarity of focus, explicit purpose given, supported by prior research |
| Does the collected data address the research question (or objectives)? | - Yes [text] - No [text] - Can’t Tell [text] | Consider whether the research and data collection strategy clearly described and was appropriate to address the research or objectives |
| Are participants (organizations) recruited in a way that minimizes selection bias? | - Yes [text] - No [text] - Can’t Tell [text] | **Cohort:** consider whether the exposed (or with intervention) and non-exposed (or without intervention) groups are recruited from the same population  **Case-control:** consider whether (a) same inclusion and exclusion criteria were applied to cases and controls; (b) whether recruitment was done independently of the intervention or exposure status; and (c) was there a sufficient number of cases/controls selected?  **Cross-sectional:** consider whether the sample is representative of the population |
| Are measurements appropriate (clear origin, or validity known, or standard instrument; and absence of contamination between groups when appropriate) regarding the exposure/intervention and outcomes? | - Yes [text] - No [text] - Can’t Tell [text] | Consider whether (a) the variable are clearly defined and accurately measured; (b) the measurements are justified and appropriate for answering the research question, and (c) the measurements reflect what they are supposed to measure |
| In the groups being compared, are the participants comparable, or do researchers take into account (control for) the differences between the groups? | - Yes [text] - No [text] - Can’t Tell [text] | Have the authours taken account of the potential confounding factors in the design and/or analysis? Consider whether (a) the most important factors are taken into account in the analysis; (b) a table lists key demographic information comparing both groups, and there are no obvious dissimilarities between groups that may account for any differences in outcomes, or dissimilarities are taken into account in the analysis |
| Are there complete outcome data (80% or above), and, when applicable, an acceptable response rate (60% or above), or an acceptable follow-up rate for cohort studies (depending on the duration of follow-up)? | - Yes [text] - No [text] - Can’t Tell [text] |  |
| Did the authors report all outcomes? | - Yes [text] - No [text] - Can’t Tell [text] | Consider (a) if there is no evidence that outcomes were selectively reported (e.g. all relevant outcomes in the methods section are reported in the results section); (b) what are the bottom line results?; and (c) are the results adjusted for confounding, and might confounding still explain the association? |
| If a questionnaire was used to measure outcomes, was it appropriately validated and reliably tested? | - Yes [text] - No [text] - Can’t Tell [text] - Not applicable [text] | Consider if the questionnaire/focus group was appropriately validated and tested |
| Was there evidence of study relevance and transferability? | - Yes [text] - No [text] - Can’t Tell [text] | Consider whether (a) there is a discussion of contribution of study to existing/prior knowledge, practice, and/or policy; (b) areas for future research identified; (c) limitations/weaknesses of study are clearly outlined; and (d) there is a discussion of whether or how the findings can be transferred to other populations or consideration of other ways the research may be used |
| Was the study free of other problems that could put it at a high risk of bias? | - Yes [text] - No [text] - Can’t Tell [text] | Consider if you have any additional concerns about the design and/or conduct and reporting of this study |
| **Mixed-Method Quality Assessment** | | |
| Was there a clear statement of the research purpose/aims? | - Yes [text] - No [text] - Can’t Tell [text] | Consider the following to make a judgement: clarity of focus, explicit purpose given, supported by prior research |
| Does the collected data address the research question (or objectives)? | - Yes [text] - No [text] - Can’t Tell [text] | Consider whether the research and data collection strategy clearly described and was appropriate to address the research or objectives |
| Is the mixed methods research design relevant to address the qualitative and quantitative research questions (or objectives)? | - Yes [text] - No [text] - Can’t Tell [text] | Consider if the rationale for integrating qualitative and quantitative methods to answer the research question is explained. |
| Is the integration of qualitative and quantitative data (or results) relevant to address the research question (objective)? | - Yes [text] - No [text] - Can’t Tell [text] | There is evidence that data gathered by both research methods was brought together to form a complete picture, and answer the research question; authours explain when integration occurred; they explain how integration occurred and who participated in this integration |
| Is appropriate consideration given to the limitations associated with this integration, e.g., the divergence of qualitative and quantitative data (or results)? | - Yes [text] - No [text] - Can’t Tell [text] |  |
| Was there evidence of study relevance and transferability? | - Yes [text] - No [text] - Can’t Tell [text] | Consider whether (a) there is a discussion of contribution of study to existing/prior knowledge, practice, and/or policy; (b) areas for future research identified; (c) limitations/weaknesses of study are clearly outlined; and (d) there is a discussion of whether or how the findings can be transferred to other populations or consideration of other ways the research may be used |
| **Final Section** | | |
| Describe any important details that you believe were not extracted. | [text] |  |

2 reviewers independently extract information from each citation.
