# Supplementary material for: Identifying contexts and mechanisms in multiple behavior change interventions affecting smoking cessation success: a rapid realist review
Source: BMC Public Health. 2020 Jun 12;20:918. doi: 10.1186/s12889-020-08973-2 (PMC7291527; doi:10.1186/s12889-020-08973-2)
Supplement: Supplementary file 3 — Additional file 3. Table 5: All Interventions That Reported Using Opportunity as one of the Mechanisms. Table 6: All Interventions That Reported Using Capability as one of the Mechanisms. Table 7: All Interventions That Reported Using Motivation as One of the Mechanisms. [file 12889_2020_8973_MOESM3_ESM.docx]

**Additional File 3**

**Table 5:** *All Interventions That Reported Using Opportunity as one of the Mechanisms.*

| **Mechanism^a^** | **Total number of interventions using this C-M-O** | **Number of interventions using this C-M-O that report improvement in smoking cessation outcome.** |
| --- | --- | --- |
| **Opportunity** | **77**  [[1-77](#_ENREF_1)] | **43 (56%)**  [[23-54](#_ENREF_23), [63-71](#_ENREF_63), [73](#_ENREF_73), [75](#_ENREF_75)] |
| **Opportunity - Access** | **34**  [[2](#_ENREF_2), [5](#_ENREF_5), [8](#_ENREF_8), [11](#_ENREF_11), [13-15](#_ENREF_13), [19](#_ENREF_19), [20](#_ENREF_20), [23-26](#_ENREF_23), [30](#_ENREF_30), [31](#_ENREF_31), [36](#_ENREF_36), [37](#_ENREF_37), [42](#_ENREF_42), [48-52](#_ENREF_48), [57-59](#_ENREF_57), [61](#_ENREF_61), [63-67](#_ENREF_63), [69](#_ENREF_69), [70](#_ENREF_70)] | **21 (62%)**  [[23-26](#_ENREF_23), [30](#_ENREF_30), [31](#_ENREF_31), [36](#_ENREF_36), [37](#_ENREF_37), [42](#_ENREF_42), [48-52](#_ENREF_48), [63-67](#_ENREF_63), [69](#_ENREF_69), [70](#_ENREF_70)] |
| **Opportunity –**  **Changing physical and/or social environment** | **32**  [[2](#_ENREF_2), [6](#_ENREF_6), [8-10](#_ENREF_8), [15](#_ENREF_15), [17](#_ENREF_17), [19](#_ENREF_19), [20](#_ENREF_20), [23](#_ENREF_23), [24](#_ENREF_24), [26](#_ENREF_26), [28](#_ENREF_28), [32](#_ENREF_32), [35](#_ENREF_35), [40-44](#_ENREF_40), [46](#_ENREF_46), [49](#_ENREF_49), [57](#_ENREF_57), [62](#_ENREF_62), [64-67](#_ENREF_64), [69-71](#_ENREF_69), [73](#_ENREF_73)] | **21 (66%)**  [[23](#_ENREF_23), [24](#_ENREF_24), [26](#_ENREF_26), [28](#_ENREF_28), [32](#_ENREF_32), [35](#_ENREF_35), [40-44](#_ENREF_40), [46](#_ENREF_46), [49](#_ENREF_49), [64-67](#_ENREF_64), [69-71](#_ENREF_69), [73](#_ENREF_73)] |
| **Opportunity – Social Support** | **33**  [[3](#_ENREF_3), [8](#_ENREF_8), [11](#_ENREF_11), [12](#_ENREF_12), [14](#_ENREF_14), [16](#_ENREF_16), [18](#_ENREF_18), [22](#_ENREF_22), [24-29](#_ENREF_24), [32](#_ENREF_32), [34](#_ENREF_34), [38](#_ENREF_38), [39](#_ENREF_39), [45](#_ENREF_45), [47](#_ENREF_47), [48](#_ENREF_48), [53-56](#_ENREF_53), [59-62](#_ENREF_59), [68](#_ENREF_68), [71](#_ENREF_71), [73](#_ENREF_73), [77](#_ENREF_77)] | **18 (55%)**  [[24-29](#_ENREF_24), [32](#_ENREF_32), [34](#_ENREF_34), [38](#_ENREF_38), [39](#_ENREF_39), [45](#_ENREF_45), [47](#_ENREF_47), [48](#_ENREF_48), [53](#_ENREF_53), [54](#_ENREF_54), [68](#_ENREF_68), [71](#_ENREF_71), [73](#_ENREF_73)] |
| **CONTEXT** | | |
| **NORTH AMERICA** | | |
| **Opportunity** | **29**  [[6](#_ENREF_6), [10](#_ENREF_10), [13](#_ENREF_13), [14](#_ENREF_14), [17](#_ENREF_17), [20](#_ENREF_20), [23](#_ENREF_23), [24](#_ENREF_24), [26](#_ENREF_26), [28](#_ENREF_28), [32](#_ENREF_32), [39](#_ENREF_39), [40](#_ENREF_40), [42](#_ENREF_42), [48](#_ENREF_48), [49](#_ENREF_49), [52](#_ENREF_52), [54-56](#_ENREF_54), [59](#_ENREF_59), [62](#_ENREF_62), [63](#_ENREF_63), [66](#_ENREF_66), [67](#_ENREF_67), [69](#_ENREF_69), [71](#_ENREF_71), [73](#_ENREF_73), [74](#_ENREF_74)] | **18 (62%)**  [[23](#_ENREF_23), [24](#_ENREF_24), [26](#_ENREF_26), [28](#_ENREF_28), [32](#_ENREF_32), [39](#_ENREF_39), [40](#_ENREF_40), [42](#_ENREF_42), [48](#_ENREF_48), [49](#_ENREF_49), [52](#_ENREF_52), [54](#_ENREF_54), [63](#_ENREF_63), [66](#_ENREF_66), [67](#_ENREF_67), [69](#_ENREF_69), [71](#_ENREF_71), [73](#_ENREF_73)] |
| **Opportunity – Access** | **15**  [[13](#_ENREF_13), [14](#_ENREF_14), [20](#_ENREF_20), [23](#_ENREF_23), [24](#_ENREF_24), [26](#_ENREF_26), [42](#_ENREF_42), [48](#_ENREF_48), [49](#_ENREF_49), [52](#_ENREF_52), [59](#_ENREF_59), [63](#_ENREF_63), [66](#_ENREF_66), [67](#_ENREF_67), [69](#_ENREF_69)] | **11 (73%)**  [[23](#_ENREF_23), [24](#_ENREF_24), [26](#_ENREF_26), [42](#_ENREF_42), [48](#_ENREF_48), [49](#_ENREF_49), [52](#_ENREF_52), [63](#_ENREF_63), [66](#_ENREF_66), [67](#_ENREF_67), [69](#_ENREF_69)] |
| **Opportunity – Changing physical and/or social environment** | **18**  [[6](#_ENREF_6), [10](#_ENREF_10), [17](#_ENREF_17), [20](#_ENREF_20), [23](#_ENREF_23), [24](#_ENREF_24), [26](#_ENREF_26), [28](#_ENREF_28), [32](#_ENREF_32), [40](#_ENREF_40), [42](#_ENREF_42), [49](#_ENREF_49), [62](#_ENREF_62), [66](#_ENREF_66), [67](#_ENREF_67), [69](#_ENREF_69), [71](#_ENREF_71), [73](#_ENREF_73)] | **13 (72%)**  [[23](#_ENREF_23), [24](#_ENREF_24), [26](#_ENREF_26), [28](#_ENREF_28), [32](#_ENREF_32), [40](#_ENREF_40), [42](#_ENREF_42), [49](#_ENREF_49), [66](#_ENREF_66), [67](#_ENREF_67), [69](#_ENREF_69), [71](#_ENREF_71), [73](#_ENREF_73)] |
| **Opportunity – Social Support** | **14**  [[14](#_ENREF_14), [24](#_ENREF_24), [26](#_ENREF_26), [28](#_ENREF_28), [32](#_ENREF_32), [39](#_ENREF_39), [48](#_ENREF_48), [54-56](#_ENREF_54), [59](#_ENREF_59), [62](#_ENREF_62), [71](#_ENREF_71), [73](#_ENREF_73)] | **9 (64%)**  [[24](#_ENREF_24), [26](#_ENREF_26), [28](#_ENREF_28), [32](#_ENREF_32), [39](#_ENREF_39), [48](#_ENREF_48), [54](#_ENREF_54), [71](#_ENREF_71), [73](#_ENREF_73)] |
| **ASIA** | | |
| **Opportunity – Social Support** | **5**  [[18](#_ENREF_18), [25](#_ENREF_25), [27](#_ENREF_27), [34](#_ENREF_34), [38](#_ENREF_38)] | **4 (80%)**  [[25](#_ENREF_25), [27](#_ENREF_27), [34](#_ENREF_34), [38](#_ENREF_38)] |
| **CLINICAL SETTING** | | |
| **Opportunity – Access** | **7**  [[37](#_ENREF_37), [48-50](#_ENREF_48), [58](#_ENREF_58), [59](#_ENREF_59), [61](#_ENREF_61)] | **4 (57%)**  [[37](#_ENREF_37), [48-50](#_ENREF_48)] |
| **COMMUNITY BASED CARE** | | |
| **Opportunity** | **23**  [[10](#_ENREF_10), [12-14](#_ENREF_12), [20](#_ENREF_20), [31](#_ENREF_31), [36](#_ENREF_36), [41](#_ENREF_41), [42](#_ENREF_42), [44](#_ENREF_44), [46](#_ENREF_46), [49](#_ENREF_49), [51](#_ENREF_51), [52](#_ENREF_52), [55](#_ENREF_55), [62](#_ENREF_62), [63](#_ENREF_63), [65](#_ENREF_65), [66](#_ENREF_66), [69](#_ENREF_69), [71](#_ENREF_71), [73](#_ENREF_73), [76](#_ENREF_76)] | **15 (65%)**  [[31](#_ENREF_31), [36](#_ENREF_36), [41](#_ENREF_41), [42](#_ENREF_42), [44](#_ENREF_44), [46](#_ENREF_46), [49](#_ENREF_49), [51](#_ENREF_51), [52](#_ENREF_52), [63](#_ENREF_63), [65](#_ENREF_65), [66](#_ENREF_66), [69](#_ENREF_69), [71](#_ENREF_71), [73](#_ENREF_73)] |
| **Opportunity – Access** | **13**  [[13](#_ENREF_13), [14](#_ENREF_14), [20](#_ENREF_20), [31](#_ENREF_31), [36](#_ENREF_36), [42](#_ENREF_42), [49](#_ENREF_49), [51](#_ENREF_51), [52](#_ENREF_52), [63](#_ENREF_63), [65](#_ENREF_65), [66](#_ENREF_66)] | **10 (77%)**  [[31](#_ENREF_31), [36](#_ENREF_36), [42](#_ENREF_42), [49](#_ENREF_49), [51](#_ENREF_51), [52](#_ENREF_52), [63](#_ENREF_63), [65](#_ENREF_65), [66](#_ENREF_66)] |
| **WORKPLACE** | | |
| **Opportunity – Access** | **11**  [[2](#_ENREF_2), [5](#_ENREF_5), [8](#_ENREF_8), [15](#_ENREF_15), [19](#_ENREF_19), [23](#_ENREF_23), [24](#_ENREF_24), [26](#_ENREF_26), [57](#_ENREF_57), [64](#_ENREF_64), [69](#_ENREF_69)] | **5 (45%)**  [[23](#_ENREF_23), [24](#_ENREF_24), [26](#_ENREF_26), [64](#_ENREF_64), [69](#_ENREF_69)] |
| **Opportunity – Social Support** | **7**  [[8](#_ENREF_8), [24](#_ENREF_24), [26](#_ENREF_26), [28](#_ENREF_28), [32](#_ENREF_32), [62](#_ENREF_62), [71](#_ENREF_71)] | **5 (71%)**  [[24](#_ENREF_24), [26](#_ENREF_26), [28](#_ENREF_28), [32](#_ENREF_32), [71](#_ENREF_71)] |
| **SCHOOLS** |  |  |
| **Opportunity** | **10**  [[6](#_ENREF_6), [35](#_ENREF_35), [41](#_ENREF_41), [43](#_ENREF_43), [44](#_ENREF_44), [62](#_ENREF_62), [66](#_ENREF_66), [67](#_ENREF_67), [69](#_ENREF_69), [70](#_ENREF_70)] | **8 (80%)**  [[35](#_ENREF_35), [41](#_ENREF_41), [43](#_ENREF_43), [44](#_ENREF_44), [66](#_ENREF_66), [67](#_ENREF_67), [69](#_ENREF_69), [70](#_ENREF_70)] |
| **Opportunity – Changing physical and/or social environment** | **10**  [[6](#_ENREF_6), [35](#_ENREF_35), [41](#_ENREF_41), [43](#_ENREF_43), [44](#_ENREF_44), [62](#_ENREF_62), [66](#_ENREF_66), [67](#_ENREF_67), [69](#_ENREF_69), [70](#_ENREF_70)] | **8 (80%)**  [[35](#_ENREF_35), [41](#_ENREF_41), [43](#_ENREF_43), [44](#_ENREF_44), [66](#_ENREF_66), [67](#_ENREF_67), [69](#_ENREF_69), [70](#_ENREF_70)] |
| **TARGET POPULATION** | | |
| **PRIMARY PREVENTION** | | |
| **Opportunity** | **51**  [[1-3](#_ENREF_1), [5-10](#_ENREF_5), [12](#_ENREF_12), [15](#_ENREF_15), [17](#_ENREF_17), [19](#_ENREF_19), [20](#_ENREF_20), [23-26](#_ENREF_23), [28](#_ENREF_28), [32](#_ENREF_32), [35](#_ENREF_35), [36](#_ENREF_36), [40-49](#_ENREF_40), [52](#_ENREF_52), [53](#_ENREF_53), [55-57](#_ENREF_55), [59](#_ENREF_59), [60](#_ENREF_60), [62-67](#_ENREF_62), [69-71](#_ENREF_69), [73](#_ENREF_73), [76](#_ENREF_76), [77](#_ENREF_77)] | **29 (57%)**  [[23-26](#_ENREF_23), [28](#_ENREF_28), [32](#_ENREF_32), [35](#_ENREF_35), [36](#_ENREF_36), [40-49](#_ENREF_40), [52](#_ENREF_52), [53](#_ENREF_53), [63-67](#_ENREF_63), [69-71](#_ENREF_69), [73](#_ENREF_73)] |
| **Opportunity – Access** | **24**  [[2](#_ENREF_2), [5](#_ENREF_5), [8](#_ENREF_8), [15](#_ENREF_15), [19](#_ENREF_19), [20](#_ENREF_20), [23-26](#_ENREF_23), [36](#_ENREF_36), [42](#_ENREF_42), [48](#_ENREF_48), [49](#_ENREF_49), [52](#_ENREF_52), [57](#_ENREF_57), [59](#_ENREF_59), [63-67](#_ENREF_63), [69](#_ENREF_69), [70](#_ENREF_70)] | **16 (67%)**  [[23-26](#_ENREF_23), [36](#_ENREF_36), [42](#_ENREF_42), [48](#_ENREF_48), [49](#_ENREF_49), [52](#_ENREF_52), [63-67](#_ENREF_63), [69](#_ENREF_69), [70](#_ENREF_70)] |
| **Opportunity – Changing physical and/or social environment** | **32**  [[2](#_ENREF_2), [6](#_ENREF_6), [8-10](#_ENREF_8), [15](#_ENREF_15), [17](#_ENREF_17), [19](#_ENREF_19), [20](#_ENREF_20), [23](#_ENREF_23), [24](#_ENREF_24), [26](#_ENREF_26), [28](#_ENREF_28), [32](#_ENREF_32), [35](#_ENREF_35), [40-44](#_ENREF_40), [46](#_ENREF_46), [49](#_ENREF_49), [57](#_ENREF_57), [62](#_ENREF_62), [64-67](#_ENREF_64), [69-71](#_ENREF_69), [73](#_ENREF_73)] | **21 (67%)**  [[23](#_ENREF_23), [24](#_ENREF_24), [26](#_ENREF_26), [28](#_ENREF_28), [32](#_ENREF_32), [35](#_ENREF_35), [40-44](#_ENREF_40), [46](#_ENREF_46), [49](#_ENREF_49), [64-67](#_ENREF_64), [69-71](#_ENREF_69), [73](#_ENREF_73)] |
| **Opportunity – Social Support** | **20**  [[3](#_ENREF_3), [8](#_ENREF_8), [12](#_ENREF_12), [24-26](#_ENREF_24), [28](#_ENREF_28), [32](#_ENREF_32), [45](#_ENREF_45), [47](#_ENREF_47), [48](#_ENREF_48), [53](#_ENREF_53), [55](#_ENREF_55), [56](#_ENREF_56), [59](#_ENREF_59), [60](#_ENREF_60), [62](#_ENREF_62), [71](#_ENREF_71), [73](#_ENREF_73), [77](#_ENREF_77)] | **11 (55%)**  [[24-26](#_ENREF_24), [28](#_ENREF_28), [32](#_ENREF_32), [45](#_ENREF_45), [47](#_ENREF_47), [48](#_ENREF_48), [53](#_ENREF_53), [71](#_ENREF_71), [73](#_ENREF_73)] |
| **SECONDARY PREVENTION** | | |
| **Opportunity** | **30**  [[11-14](#_ENREF_11), [16](#_ENREF_16), [18](#_ENREF_18), [20](#_ENREF_20), [21](#_ENREF_21), [25](#_ENREF_25), [27](#_ENREF_27), [30-34](#_ENREF_30), [37-39](#_ENREF_37), [41](#_ENREF_41), [50](#_ENREF_50), [51](#_ENREF_51), [53](#_ENREF_53), [54](#_ENREF_54), [58](#_ENREF_58), [61](#_ENREF_61), [68](#_ENREF_68), [72](#_ENREF_72), [74](#_ENREF_74), [75](#_ENREF_75), [77](#_ENREF_77)] | **17 (57%)**  [[25](#_ENREF_25), [27](#_ENREF_27), [30-34](#_ENREF_30), [37-39](#_ENREF_37), [41](#_ENREF_41), [50](#_ENREF_50), [51](#_ENREF_51), [53](#_ENREF_53), [54](#_ENREF_54), [68](#_ENREF_68), [75](#_ENREF_75)] |
| **Opportunity – Access** | **12**  [[11](#_ENREF_11), [13](#_ENREF_13), [14](#_ENREF_14), [20](#_ENREF_20), [25](#_ENREF_25), [30](#_ENREF_30), [31](#_ENREF_31), [37](#_ENREF_37), [50](#_ENREF_50), [51](#_ENREF_51), [58](#_ENREF_58), [61](#_ENREF_61)] | **6 (50%)**  [[25](#_ENREF_25), [30](#_ENREF_30), [31](#_ENREF_31), [37](#_ENREF_37), [50](#_ENREF_50), [51](#_ENREF_51)] |
| **Opportunity – Social Support** | **15**  [[11](#_ENREF_11), [12](#_ENREF_12), [14](#_ENREF_14), [16](#_ENREF_16), [18](#_ENREF_18), [25](#_ENREF_25), [27](#_ENREF_27), [32](#_ENREF_32), [34](#_ENREF_34), [38](#_ENREF_38), [39](#_ENREF_39), [53](#_ENREF_53), [54](#_ENREF_54), [61](#_ENREF_61), [68](#_ENREF_68), [77](#_ENREF_77)] | **9 (60%)**  [[25](#_ENREF_25), [27](#_ENREF_27), [32](#_ENREF_32), [34](#_ENREF_34), [38](#_ENREF_38), [39](#_ENREF_39), [53](#_ENREF_53), [54](#_ENREF_54), [68](#_ENREF_68)] |
| **TYPE OF MODIFIABLE RISK BEHAVIOUR INTERVENTION ADDRESSED^b^** | | |
| **ALCOHOL** | | |
| **Opportunity** | **29**  [[1](#_ENREF_1), [2](#_ENREF_2), [5-7](#_ENREF_5), [9](#_ENREF_9), [12](#_ENREF_12), [14](#_ENREF_14), [21](#_ENREF_21), [23-25](#_ENREF_23), [28-30](#_ENREF_28), [32](#_ENREF_32), [34](#_ENREF_34), [35](#_ENREF_35), [39](#_ENREF_39), [40](#_ENREF_40), [47](#_ENREF_47), [48](#_ENREF_48), [51](#_ENREF_51), [55](#_ENREF_55), [59](#_ENREF_59), [67](#_ENREF_67), [72](#_ENREF_72), [75](#_ENREF_75), [76](#_ENREF_76)] | **16 (55%)**  [[23-25](#_ENREF_23), [28-30](#_ENREF_28), [32](#_ENREF_32), [34](#_ENREF_34), [35](#_ENREF_35), [39](#_ENREF_39), [40](#_ENREF_40), [47](#_ENREF_47), [48](#_ENREF_48), [51](#_ENREF_51), [67](#_ENREF_67), [75](#_ENREF_75)] |
| **Opportunity – Access** | **11**  [[2](#_ENREF_2), [5](#_ENREF_5), [14](#_ENREF_14), [23-25](#_ENREF_23), [30](#_ENREF_30), [48](#_ENREF_48), [51](#_ENREF_51), [59](#_ENREF_59), [67](#_ENREF_67)] | **7 (64%)**  [[23-25](#_ENREF_23), [30](#_ENREF_30), [48](#_ENREF_48), [51](#_ENREF_51), [67](#_ENREF_67)] |
| **Opportunity – Changing physical and/or social environment** | **10**  [[2](#_ENREF_2), [6](#_ENREF_6), [9](#_ENREF_9), [23](#_ENREF_23), [24](#_ENREF_24), [28](#_ENREF_28), [32](#_ENREF_32), [35](#_ENREF_35), [40](#_ENREF_40), [67](#_ENREF_67)] | **7 (70%)**  [[23](#_ENREF_23), [24](#_ENREF_24), [28](#_ENREF_28), [32](#_ENREF_32), [35](#_ENREF_35), [40](#_ENREF_40), [67](#_ENREF_67)] |
| **Opportunity – Social Support** | **13**  [[12](#_ENREF_12), [14](#_ENREF_14), [24](#_ENREF_24), [25](#_ENREF_25), [28](#_ENREF_28), [29](#_ENREF_29), [32](#_ENREF_32), [34](#_ENREF_34), [39](#_ENREF_39), [47](#_ENREF_47), [48](#_ENREF_48), [55](#_ENREF_55), [59](#_ENREF_59)] | **9 (69%)**  [[24](#_ENREF_24), [25](#_ENREF_25), [28](#_ENREF_28), [29](#_ENREF_29), [32](#_ENREF_32), [34](#_ENREF_34), [39](#_ENREF_39), [47](#_ENREF_47), [48](#_ENREF_48)] |
| **DIET** | | |
| **Opportunity – Access** | **32**  [[2](#_ENREF_2), [5](#_ENREF_5), [8](#_ENREF_8), [11](#_ENREF_11), [13-15](#_ENREF_13), [19](#_ENREF_19), [20](#_ENREF_20), [23-26](#_ENREF_23), [31](#_ENREF_31), [36](#_ENREF_36), [37](#_ENREF_37), [42](#_ENREF_42), [48-50](#_ENREF_48), [52](#_ENREF_52), [57-59](#_ENREF_57), [61](#_ENREF_61), [63-67](#_ENREF_63), [69](#_ENREF_69), [70](#_ENREF_70)] | **19 (59%)**  [[23-26](#_ENREF_23), [31](#_ENREF_31), [36](#_ENREF_36), [37](#_ENREF_37), [42](#_ENREF_42), [48-50](#_ENREF_48), [52](#_ENREF_52), [63-67](#_ENREF_63), [69](#_ENREF_69), [70](#_ENREF_70)] |
| **Opportunity – Changing physical and/or social environment** | **32**  [[2](#_ENREF_2), [6](#_ENREF_6), [8-10](#_ENREF_8), [15](#_ENREF_15), [17](#_ENREF_17), [19](#_ENREF_19), [20](#_ENREF_20), [23](#_ENREF_23), [24](#_ENREF_24), [26](#_ENREF_26), [28](#_ENREF_28), [32](#_ENREF_32), [35](#_ENREF_35), [40-44](#_ENREF_40), [46](#_ENREF_46), [49](#_ENREF_49), [57](#_ENREF_57), [62](#_ENREF_62), [64-67](#_ENREF_64), [69-71](#_ENREF_69), [73](#_ENREF_73)] | **21 (66%)**  [[23](#_ENREF_23), [24](#_ENREF_24), [26](#_ENREF_26), [28](#_ENREF_28), [32](#_ENREF_32), [35](#_ENREF_35), [40-44](#_ENREF_40), [46](#_ENREF_46), [49](#_ENREF_49), [64-67](#_ENREF_64), [69-71](#_ENREF_69), [73](#_ENREF_73)] |
| **Opportunity – Social Support** | **32**  [[3](#_ENREF_3), [8](#_ENREF_8), [11](#_ENREF_11), [12](#_ENREF_12), [14](#_ENREF_14), [16](#_ENREF_16), [18](#_ENREF_18), [22](#_ENREF_22), [24-29](#_ENREF_24), [32](#_ENREF_32), [34](#_ENREF_34), [38](#_ENREF_38), [45](#_ENREF_45), [47](#_ENREF_47), [48](#_ENREF_48), [53-56](#_ENREF_53), [59-62](#_ENREF_59), [68](#_ENREF_68), [71](#_ENREF_71), [73](#_ENREF_73), [77](#_ENREF_77)] | **17 (53%)**  [[24-29](#_ENREF_24), [32](#_ENREF_32), [34](#_ENREF_34), [38](#_ENREF_38), [45](#_ENREF_45), [47](#_ENREF_47), [48](#_ENREF_48), [53](#_ENREF_53), [54](#_ENREF_54), [68](#_ENREF_68), [71](#_ENREF_71), [73](#_ENREF_73)] |
| **PHYSICAL ACTIVITY** | | |
| **Opportunity** | **76**  [[1-74](#_ENREF_1), [76](#_ENREF_76), [77](#_ENREF_77)] | **42 (55%)**  [[23-54](#_ENREF_23), [63-71](#_ENREF_63), [73](#_ENREF_73)] |
| **Opportunity – Access** | **34**  [[2](#_ENREF_2), [5](#_ENREF_5), [8](#_ENREF_8), [11](#_ENREF_11), [13-15](#_ENREF_13), [19](#_ENREF_19), [20](#_ENREF_20), [23-26](#_ENREF_23), [30](#_ENREF_30), [31](#_ENREF_31), [36](#_ENREF_36), [37](#_ENREF_37), [42](#_ENREF_42), [48-52](#_ENREF_48), [57-59](#_ENREF_57), [61](#_ENREF_61), [63-67](#_ENREF_63), [69](#_ENREF_69), [70](#_ENREF_70)] | **21 (62%)**  [[23-26](#_ENREF_23), [30](#_ENREF_30), [31](#_ENREF_31), [36](#_ENREF_36), [37](#_ENREF_37), [42](#_ENREF_42), [48-52](#_ENREF_48), [63-67](#_ENREF_63), [69](#_ENREF_69), [70](#_ENREF_70)] |
| **Opportunity – Changing physical and/or social environment** | **32**  [[2](#_ENREF_2), [6](#_ENREF_6), [8-10](#_ENREF_8), [15](#_ENREF_15), [17](#_ENREF_17), [19](#_ENREF_19), [20](#_ENREF_20), [23](#_ENREF_23), [24](#_ENREF_24), [26](#_ENREF_26), [28](#_ENREF_28), [32](#_ENREF_32), [35](#_ENREF_35), [40-44](#_ENREF_40), [46](#_ENREF_46), [49](#_ENREF_49), [57](#_ENREF_57), [62](#_ENREF_62), [64-67](#_ENREF_64), [69-71](#_ENREF_69), [73](#_ENREF_73)] | **21 (66%)**  [[23](#_ENREF_23), [24](#_ENREF_24), [26](#_ENREF_26), [28](#_ENREF_28), [32](#_ENREF_32), [35](#_ENREF_35), [40-44](#_ENREF_40), [46](#_ENREF_46), [49](#_ENREF_49), [64-67](#_ENREF_64), [69-71](#_ENREF_69), [73](#_ENREF_73)] |
| **Opportunity – Social Support** | **33**  [[3](#_ENREF_3), [8](#_ENREF_8), [11](#_ENREF_11), [12](#_ENREF_12), [14](#_ENREF_14), [16](#_ENREF_16), [18](#_ENREF_18), [22](#_ENREF_22), [24-29](#_ENREF_24), [32](#_ENREF_32), [34](#_ENREF_34), [38](#_ENREF_38), [39](#_ENREF_39), [45](#_ENREF_45), [47](#_ENREF_47), [48](#_ENREF_48), [53-56](#_ENREF_53), [59-62](#_ENREF_59), [68](#_ENREF_68), [71](#_ENREF_71), [73](#_ENREF_73), [77](#_ENREF_77)] | **18 (55%)**  [[24-29](#_ENREF_24), [32](#_ENREF_32), [34](#_ENREF_34), [38](#_ENREF_38), [39](#_ENREF_39), [45](#_ENREF_45), [47](#_ENREF_47), [48](#_ENREF_48), [53](#_ENREF_53), [54](#_ENREF_54), [68](#_ENREF_68), [71](#_ENREF_71), [73](#_ENREF_73)] |
| **STRESS** | | |
| **Opportunity** | **25**  [[5](#_ENREF_5), [8](#_ENREF_8), [9](#_ENREF_9), [13-15](#_ENREF_13), [23](#_ENREF_23), [24](#_ENREF_24), [26](#_ENREF_26), [28](#_ENREF_28), [31](#_ENREF_31), [32](#_ENREF_32), [38](#_ENREF_38), [41](#_ENREF_41), [42](#_ENREF_42), [44](#_ENREF_44), [47](#_ENREF_47), [50](#_ENREF_50), [54](#_ENREF_54), [55](#_ENREF_55), [63](#_ENREF_63), [64](#_ENREF_64), [72](#_ENREF_72), [74](#_ENREF_74), [75](#_ENREF_75)] | **16 (64%)**  [[23](#_ENREF_23), [24](#_ENREF_24), [26](#_ENREF_26), [28](#_ENREF_28), [31](#_ENREF_31), [32](#_ENREF_32), [38](#_ENREF_38), [41](#_ENREF_41), [42](#_ENREF_42), [44](#_ENREF_44), [47](#_ENREF_47), [50](#_ENREF_50), [54](#_ENREF_54), [63](#_ENREF_63), [64](#_ENREF_64), [75](#_ENREF_75)] |
| **Opportunity – Access** | **13**  [[5](#_ENREF_5), [8](#_ENREF_8), [13-15](#_ENREF_13), [23](#_ENREF_23), [24](#_ENREF_24), [26](#_ENREF_26), [31](#_ENREF_31), [42](#_ENREF_42), [50](#_ENREF_50), [63](#_ENREF_63), [64](#_ENREF_64)] | **8 (62%)**  [[23](#_ENREF_23), [24](#_ENREF_24), [26](#_ENREF_26), [31](#_ENREF_31), [42](#_ENREF_42), [50](#_ENREF_50), [63](#_ENREF_63), [64](#_ENREF_64)] |
| **Opportunity – Changing physical and/or social environment** | **12**  [[8](#_ENREF_8), [9](#_ENREF_9), [15](#_ENREF_15), [23](#_ENREF_23), [24](#_ENREF_24), [26](#_ENREF_26), [28](#_ENREF_28), [32](#_ENREF_32), [41](#_ENREF_41), [42](#_ENREF_42), [44](#_ENREF_44), [64](#_ENREF_64)] | **9 (75%)**  [[23](#_ENREF_23), [24](#_ENREF_24), [26](#_ENREF_26), [28](#_ENREF_28), [32](#_ENREF_32), [41](#_ENREF_41), [42](#_ENREF_42), [44](#_ENREF_44), [64](#_ENREF_64)] |
| **Opportunity – Social Support** | **10**  [[8](#_ENREF_8), [14](#_ENREF_14), [24](#_ENREF_24), [26](#_ENREF_26), [28](#_ENREF_28), [32](#_ENREF_32), [38](#_ENREF_38), [47](#_ENREF_47), [54](#_ENREF_54), [55](#_ENREF_55)] | **7 (70%)**  [[24](#_ENREF_24), [26](#_ENREF_26), [28](#_ENREF_28), [32](#_ENREF_32), [38](#_ENREF_38), [47](#_ENREF_47), [54](#_ENREF_54)] |
| **NUMBER OF MODIFIABLE RISK BEHAVIOURS ADDRESSED** | | |
| **3 BEHAVIOURS** | | |
| **Opportunity** | **38**  [[3](#_ENREF_3), [4](#_ENREF_4), [10](#_ENREF_10), [11](#_ENREF_11), [16-20](#_ENREF_16), [22](#_ENREF_22), [27](#_ENREF_27), [30](#_ENREF_30), [33](#_ENREF_33), [36](#_ENREF_36), [37](#_ENREF_37), [39](#_ENREF_39), [43](#_ENREF_43), [45](#_ENREF_45), [46](#_ENREF_46), [49](#_ENREF_49), [51-53](#_ENREF_51), [56-58](#_ENREF_56), [60-62](#_ENREF_60), [65](#_ENREF_65), [66](#_ENREF_66), [68-71](#_ENREF_68), [73](#_ENREF_73), [75](#_ENREF_75), [77](#_ENREF_77)] | **21 (55%)**  [[27](#_ENREF_27), [30](#_ENREF_30), [33](#_ENREF_33), [36](#_ENREF_36), [37](#_ENREF_37), [39](#_ENREF_39), [43](#_ENREF_43), [45](#_ENREF_45), [46](#_ENREF_46), [49](#_ENREF_49), [51-53](#_ENREF_51), [65](#_ENREF_65), [66](#_ENREF_66), [68-71](#_ENREF_68), [73](#_ENREF_73), [75](#_ENREF_75)] |
| **Opportunity – Access** | **16**  [[11](#_ENREF_11), [19](#_ENREF_19), [20](#_ENREF_20), [30](#_ENREF_30), [36](#_ENREF_36), [37](#_ENREF_37), [49](#_ENREF_49), [51](#_ENREF_51), [52](#_ENREF_52), [57](#_ENREF_57), [58](#_ENREF_58), [61](#_ENREF_61), [65](#_ENREF_65), [66](#_ENREF_66), [69](#_ENREF_69), [70](#_ENREF_70)] | **10 (63%)**  [[30](#_ENREF_30), [36](#_ENREF_36), [37](#_ENREF_37), [49](#_ENREF_49), [51](#_ENREF_51), [52](#_ENREF_52), [65](#_ENREF_65), [66](#_ENREF_66), [69](#_ENREF_69), [70](#_ENREF_70)] |
| **Opportunity – Changing physical and/or social environment** | **15**  [[10](#_ENREF_10), [17](#_ENREF_17), [19](#_ENREF_19), [20](#_ENREF_20), [43](#_ENREF_43), [46](#_ENREF_46), [49](#_ENREF_49), [57](#_ENREF_57), [62](#_ENREF_62), [65](#_ENREF_65), [66](#_ENREF_66), [69-71](#_ENREF_69), [73](#_ENREF_73)] | **9 (60%)**  [[43](#_ENREF_43), [46](#_ENREF_46), [49](#_ENREF_49), [65](#_ENREF_65), [66](#_ENREF_66), [69-71](#_ENREF_69), [73](#_ENREF_73)] |
| **4 BEHAVIOURS** | | |
| **Opportunity** | **30**  [[1](#_ENREF_1), [2](#_ENREF_2), [6-8](#_ENREF_6), [12](#_ENREF_12), [13](#_ENREF_13), [15](#_ENREF_15), [21](#_ENREF_21), [25](#_ENREF_25), [26](#_ENREF_26), [29](#_ENREF_29), [31](#_ENREF_31), [34](#_ENREF_34), [35](#_ENREF_35), [38](#_ENREF_38), [40-42](#_ENREF_40), [44](#_ENREF_44), [48](#_ENREF_48), [50](#_ENREF_50), [54](#_ENREF_54), [59](#_ENREF_59), [63](#_ENREF_63), [64](#_ENREF_64), [67](#_ENREF_67), [72](#_ENREF_72), [74](#_ENREF_74), [76](#_ENREF_76)] | **17 (57%)**  [[25](#_ENREF_25), [26](#_ENREF_26), [29](#_ENREF_29), [31](#_ENREF_31), [34](#_ENREF_34), [35](#_ENREF_35), [38](#_ENREF_38), [40-42](#_ENREF_40), [44](#_ENREF_44), [48](#_ENREF_48), [50](#_ENREF_50), [54](#_ENREF_54), [63](#_ENREF_63), [64](#_ENREF_64), [67](#_ENREF_67)] |
| **Opportunity – Access** | **14**  [[2](#_ENREF_2), [8](#_ENREF_8), [13](#_ENREF_13), [15](#_ENREF_15), [25](#_ENREF_25), [26](#_ENREF_26), [31](#_ENREF_31), [42](#_ENREF_42), [48](#_ENREF_48), [50](#_ENREF_50), [59](#_ENREF_59), [63](#_ENREF_63), [64](#_ENREF_64), [67](#_ENREF_67)] | **9 (64%)**  [[25](#_ENREF_25), [26](#_ENREF_26), [31](#_ENREF_31), [42](#_ENREF_42), [48](#_ENREF_48), [50](#_ENREF_50), [63](#_ENREF_63), [64](#_ENREF_64), [67](#_ENREF_67)] |
| **Opportunity – Changing and physical social environment** | **12**  [[2](#_ENREF_2), [6](#_ENREF_6), [8](#_ENREF_8), [15](#_ENREF_15), [26](#_ENREF_26), [35](#_ENREF_35), [40-42](#_ENREF_40), [44](#_ENREF_44), [64](#_ENREF_64), [67](#_ENREF_67)] | **8 (67%)**  [[26](#_ENREF_26), [35](#_ENREF_35), [40-42](#_ENREF_40), [44](#_ENREF_44), [64](#_ENREF_64), [67](#_ENREF_67)] |
| **Opportunity – Social Support** | **10**  [[8](#_ENREF_8), [12](#_ENREF_12), [25](#_ENREF_25), [26](#_ENREF_26), [29](#_ENREF_29), [34](#_ENREF_34), [38](#_ENREF_38), [48](#_ENREF_48), [54](#_ENREF_54), [59](#_ENREF_59)] | **7 (70%)**  [[25](#_ENREF_25), [26](#_ENREF_26), [29](#_ENREF_29), [34](#_ENREF_34), [38](#_ENREF_38), [48](#_ENREF_48), [54](#_ENREF_54)] |
| **5 BEHAVIOURS** | | |
| **Opportunity – Social Support** | **5**  [[14](#_ENREF_14), [24](#_ENREF_24), [32](#_ENREF_32), [47](#_ENREF_47), [55](#_ENREF_55)] | **3 (60%)**  [[24](#_ENREF_24), [32](#_ENREF_32), [47](#_ENREF_47)] |

## ^a^ The mechanisms within these intervention may not be exclusively for addressing smoking behaviour. It could be part of the overall intervention or for other risk behaviours within that intervention.

## ^b^ This category examines interventions that include the specific risk behaviour (i.e. alcohol) as one of the targeted behaviours.

**Table 6:** *All Interventions That Reported Using Capability as one of the Mechanisms.*

| **Mechanism^a^** | **Total number of interventions using this C-M-O** | **Number of interventions using this C-M-O that report improvement in smoking cessation outcome.** |
| --- | --- | --- |
| **Capability** | **132**  [[1-19](#_ENREF_1), [21-133](#_ENREF_21)] | **70 (53%)**  [[23-54](#_ENREF_23), [63-71](#_ENREF_63), [73](#_ENREF_73), [75](#_ENREF_75), [106-132](#_ENREF_106)] |
| **Capability – Beliefs about the intervention** | **7**  [[22](#_ENREF_22), [34](#_ENREF_34), [42](#_ENREF_42), [51](#_ENREF_51), [73](#_ENREF_73), [81](#_ENREF_81), [82](#_ENREF_82)] | **4 (57%)**  [[34](#_ENREF_34), [42](#_ENREF_42), [51](#_ENREF_51), [73](#_ENREF_73)] |
| **Capability – Empowerment** | **22**  [[3](#_ENREF_3), [10](#_ENREF_10), [16](#_ENREF_16), [23](#_ENREF_23), [24](#_ENREF_24), [27](#_ENREF_27), [29](#_ENREF_29), [32](#_ENREF_32), [34](#_ENREF_34), [35](#_ENREF_35), [45](#_ENREF_45), [47](#_ENREF_47), [72](#_ENREF_72), [82](#_ENREF_82), [89](#_ENREF_89), [91](#_ENREF_91), [93](#_ENREF_93), [94](#_ENREF_94), [96](#_ENREF_96), [99](#_ENREF_99), [103](#_ENREF_103), [107](#_ENREF_107)] | **10 (45%)**  [[23](#_ENREF_23), [24](#_ENREF_24), [27](#_ENREF_27), [29](#_ENREF_29), [32](#_ENREF_32), [34](#_ENREF_34), [35](#_ENREF_35), [45](#_ENREF_45), [47](#_ENREF_47), [107](#_ENREF_107)] |
| **CONTEXT** | | |
| **NORTH AMERICA** | | |
| **Capability - Empowerment** | **6**  [[10](#_ENREF_10), [23](#_ENREF_23), [24](#_ENREF_24), [32](#_ENREF_32), [96](#_ENREF_96), [99](#_ENREF_99)] | **3 (50%)**  [[23](#_ENREF_23), [24](#_ENREF_24), [32](#_ENREF_32)] |
| **EUROPE** | | |
| **Capability** | **56**  [[1-8](#_ENREF_1), [11](#_ENREF_11), [16](#_ENREF_16), [22](#_ENREF_22), [29](#_ENREF_29), [31](#_ENREF_31), [33](#_ENREF_33), [35-37](#_ENREF_35), [44-46](#_ENREF_44), [50](#_ENREF_50), [53](#_ENREF_53), [57](#_ENREF_57), [58](#_ENREF_58), [60](#_ENREF_60), [61](#_ENREF_61), [64](#_ENREF_64), [68](#_ENREF_68), [72](#_ENREF_72), [75](#_ENREF_75), [77](#_ENREF_77), [80-83](#_ENREF_80), [85](#_ENREF_85), [87](#_ENREF_87), [89](#_ENREF_89), [91-94](#_ENREF_91), [101-103](#_ENREF_101), [107](#_ENREF_107), [112](#_ENREF_112), [118](#_ENREF_118), [120](#_ENREF_120), [121](#_ENREF_121), [123](#_ENREF_123), [126-128](#_ENREF_126), [132](#_ENREF_132), [133](#_ENREF_133)] | **24 (43%)**  [[29](#_ENREF_29), [31](#_ENREF_31), [33](#_ENREF_33), [35-37](#_ENREF_35), [44-46](#_ENREF_44), [50](#_ENREF_50), [53](#_ENREF_53), [64](#_ENREF_64), [68](#_ENREF_68), [75](#_ENREF_75), [107](#_ENREF_107), [112](#_ENREF_112), [118](#_ENREF_118), [120](#_ENREF_120), [121](#_ENREF_121), [123](#_ENREF_123), [126-128](#_ENREF_126), [132](#_ENREF_132)] |
| **Capability - Beliefs about interventions** | **3**  [[22](#_ENREF_22), [81](#_ENREF_81), [82](#_ENREF_82)] | **0 (0%)** |
| **Capability - Capacity to plan** | **36**  [[1](#_ENREF_1), [3](#_ENREF_3), [4](#_ENREF_4), [6](#_ENREF_6), [16](#_ENREF_16), [22](#_ENREF_22), [29](#_ENREF_29), [36](#_ENREF_36), [37](#_ENREF_37), [44](#_ENREF_44), [45](#_ENREF_45), [50](#_ENREF_50), [53](#_ENREF_53), [57](#_ENREF_57), [60](#_ENREF_60), [61](#_ENREF_61), [68](#_ENREF_68), [72](#_ENREF_72), [75](#_ENREF_75), [77](#_ENREF_77), [80](#_ENREF_80), [82](#_ENREF_82), [83](#_ENREF_83), [89](#_ENREF_89), [92](#_ENREF_92), [94](#_ENREF_94), [101-103](#_ENREF_101), [112](#_ENREF_112), [118](#_ENREF_118), [120](#_ENREF_120), [123](#_ENREF_123), [126](#_ENREF_126), [128](#_ENREF_128), [132](#_ENREF_132), [133](#_ENREF_133)] | **16 (44%)**  [[29](#_ENREF_29), [36](#_ENREF_36), [37](#_ENREF_37), [44](#_ENREF_44), [45](#_ENREF_45), [50](#_ENREF_50), [53](#_ENREF_53), [68](#_ENREF_68), [75](#_ENREF_75), [112](#_ENREF_112), [118](#_ENREF_118), [120](#_ENREF_120), [123](#_ENREF_123), [126](#_ENREF_126), [128](#_ENREF_128), [132](#_ENREF_132)] |
| **Capability - Empowerment** | **13**  [[3](#_ENREF_3), [16](#_ENREF_16), [29](#_ENREF_29), [35](#_ENREF_35), [45](#_ENREF_45), [72](#_ENREF_72), [82](#_ENREF_82), [89](#_ENREF_89), [91](#_ENREF_91), [93](#_ENREF_93), [94](#_ENREF_94), [103](#_ENREF_103), [107](#_ENREF_107)] | **4 (31%)**  [[29](#_ENREF_29), [45](#_ENREF_45), [107](#_ENREF_107), [134](#_ENREF_134)] |
| **Capability - Enhance knowledge and skills of individual** | **55**  [[1-8](#_ENREF_1), [11](#_ENREF_11), [16](#_ENREF_16), [29](#_ENREF_29), [31](#_ENREF_31), [33](#_ENREF_33), [35-37](#_ENREF_35), [44-46](#_ENREF_44), [50](#_ENREF_50), [53](#_ENREF_53), [57](#_ENREF_57), [58](#_ENREF_58), [60](#_ENREF_60), [61](#_ENREF_61), [64](#_ENREF_64), [68](#_ENREF_68), [72](#_ENREF_72), [75](#_ENREF_75), [77](#_ENREF_77), [80-83](#_ENREF_80), [85](#_ENREF_85), [87](#_ENREF_87), [89](#_ENREF_89), [91-94](#_ENREF_91), [101-103](#_ENREF_101), [107](#_ENREF_107), [112](#_ENREF_112), [118](#_ENREF_118), [120](#_ENREF_120), [121](#_ENREF_121), [123](#_ENREF_123), [126-128](#_ENREF_126), [132](#_ENREF_132), [133](#_ENREF_133)] | **24 (44%)**  [[29](#_ENREF_29), [31](#_ENREF_31), [33](#_ENREF_33), [35-37](#_ENREF_35), [44-46](#_ENREF_44), [50](#_ENREF_50), [53](#_ENREF_53), [64](#_ENREF_64), [68](#_ENREF_68), [75](#_ENREF_75), [107](#_ENREF_107), [112](#_ENREF_112), [118](#_ENREF_118), [120](#_ENREF_120), [121](#_ENREF_121), [123](#_ENREF_123), [126-128](#_ENREF_126), [132](#_ENREF_132)] |
| **ASIA** | | |
| **Capability** | **18**  [[9](#_ENREF_9), [15](#_ENREF_15), [18](#_ENREF_18), [21](#_ENREF_21), [25](#_ENREF_25), [27](#_ENREF_27), [34](#_ENREF_34), [38](#_ENREF_38), [41](#_ENREF_41), [51](#_ENREF_51), [65](#_ENREF_65), [70](#_ENREF_70), [76](#_ENREF_76), [106](#_ENREF_106), [108](#_ENREF_108), [122](#_ENREF_122), [129](#_ENREF_129), [130](#_ENREF_130)] | **13 (72%)**  [[25](#_ENREF_25), [27](#_ENREF_27), [34](#_ENREF_34), [38](#_ENREF_38), [41](#_ENREF_41), [51](#_ENREF_51), [65](#_ENREF_65), [70](#_ENREF_70), [106](#_ENREF_106), [108](#_ENREF_108), [122](#_ENREF_122), [129](#_ENREF_129), [130](#_ENREF_130)] |
| **Capability - Capacity to plan** | **14**  [[15](#_ENREF_15), [18](#_ENREF_18), [21](#_ENREF_21), [25](#_ENREF_25), [34](#_ENREF_34), [38](#_ENREF_38), [41](#_ENREF_41), [51](#_ENREF_51), [65](#_ENREF_65), [106](#_ENREF_106), [108](#_ENREF_108), [122](#_ENREF_122), [129](#_ENREF_129), [130](#_ENREF_130)] | **11 (79%)**  [[25](#_ENREF_25), [34](#_ENREF_34), [38](#_ENREF_38), [41](#_ENREF_41), [51](#_ENREF_51), [65](#_ENREF_65), [106](#_ENREF_106), [108](#_ENREF_108), [122](#_ENREF_122), [129](#_ENREF_129), [130](#_ENREF_130)] |
| **Capability - Enhance knowledge and skills of individual** | **17**  [[9](#_ENREF_9), [15](#_ENREF_15), [18](#_ENREF_18), [21](#_ENREF_21), [25](#_ENREF_25), [27](#_ENREF_27), [34](#_ENREF_34), [38](#_ENREF_38), [41](#_ENREF_41), [51](#_ENREF_51), [65](#_ENREF_65), [70](#_ENREF_70), [106](#_ENREF_106), [108](#_ENREF_108), [122](#_ENREF_122), [129](#_ENREF_129), [130](#_ENREF_130)] | **13 (76%)**  [[25](#_ENREF_25), [27](#_ENREF_27), [34](#_ENREF_34), [38](#_ENREF_38), [41](#_ENREF_41), [51](#_ENREF_51), [65](#_ENREF_65), [70](#_ENREF_70), [106](#_ENREF_106), [108](#_ENREF_108), [122](#_ENREF_122), [129](#_ENREF_129), [130](#_ENREF_130)] |
| **AUSTRALASIA** | | |
| **Capability** | **9**  [[12](#_ENREF_12), [30](#_ENREF_30), [47](#_ENREF_47), [78](#_ENREF_78), [79](#_ENREF_79), [88](#_ENREF_88), [97](#_ENREF_97), [117](#_ENREF_117), [125](#_ENREF_125)] | **4 (44%)**  [[30](#_ENREF_30), [117](#_ENREF_117), [125](#_ENREF_125), [135](#_ENREF_135)] |
| **Capability - Capacity to plan** | **7**  [[12](#_ENREF_12), [47](#_ENREF_47), [78](#_ENREF_78), [79](#_ENREF_79), [97](#_ENREF_97), [117](#_ENREF_117), [125](#_ENREF_125)] | **3 (43%)**  [[47](#_ENREF_47), [117](#_ENREF_117), [125](#_ENREF_125)] |
| **CLINICAL SETTING** | | |
| **Capability** | **58**  [[3](#_ENREF_3), [4](#_ENREF_4), [7](#_ENREF_7), [18](#_ENREF_18), [22](#_ENREF_22), [27](#_ENREF_27), [29](#_ENREF_29), [33](#_ENREF_33), [34](#_ENREF_34), [37](#_ENREF_37), [40](#_ENREF_40), [41](#_ENREF_41), [44](#_ENREF_44), [48-50](#_ENREF_48), [53-56](#_ENREF_53), [58](#_ENREF_58), [59](#_ENREF_59), [61](#_ENREF_61), [68](#_ENREF_68), [72-75](#_ENREF_72), [77](#_ENREF_77), [79](#_ENREF_79), [81](#_ENREF_81), [84-86](#_ENREF_84), [89-92](#_ENREF_89), [94](#_ENREF_94), [99-102](#_ENREF_99), [105](#_ENREF_105), [107-109](#_ENREF_107), [111](#_ENREF_111), [113](#_ENREF_113), [115](#_ENREF_115), [117](#_ENREF_117), [120](#_ENREF_120), [122-126](#_ENREF_122), [130](#_ENREF_130)] | **30 (52%)**  [[27](#_ENREF_27), [29](#_ENREF_29), [33](#_ENREF_33), [34](#_ENREF_34), [37](#_ENREF_37), [40](#_ENREF_40), [41](#_ENREF_41), [44](#_ENREF_44), [48-50](#_ENREF_48), [53](#_ENREF_53), [54](#_ENREF_54), [68](#_ENREF_68), [73](#_ENREF_73), [75](#_ENREF_75), [107-109](#_ENREF_107), [111](#_ENREF_111), [113](#_ENREF_113), [115](#_ENREF_115), [117](#_ENREF_117), [120](#_ENREF_120), [122-126](#_ENREF_122), [130](#_ENREF_130)] |
| **Capability – Beliefs about intervention** | **4**  [[22](#_ENREF_22), [34](#_ENREF_34), [73](#_ENREF_73), [81](#_ENREF_81)] | **2 (50%)**  [[34](#_ENREF_34), [73](#_ENREF_73)] |
| **Capability – Capacity to Plan** | **42**  [[3](#_ENREF_3), [4](#_ENREF_4), [18](#_ENREF_18), [22](#_ENREF_22), [29](#_ENREF_29), [34](#_ENREF_34), [37](#_ENREF_37), [41](#_ENREF_41), [44](#_ENREF_44), [48](#_ENREF_48), [50](#_ENREF_50), [53-56](#_ENREF_53), [59](#_ENREF_59), [68](#_ENREF_68), [72](#_ENREF_72), [73](#_ENREF_73), [75](#_ENREF_75), [77](#_ENREF_77), [79](#_ENREF_79), [84](#_ENREF_84), [86](#_ENREF_86), [89](#_ENREF_89), [90](#_ENREF_90), [92](#_ENREF_92), [94](#_ENREF_94), [99-102](#_ENREF_99), [108](#_ENREF_108), [117](#_ENREF_117), [120](#_ENREF_120), [122-126](#_ENREF_122), [130](#_ENREF_130)] | **21 (50%)**  [[29](#_ENREF_29), [34](#_ENREF_34), [37](#_ENREF_37), [41](#_ENREF_41), [44](#_ENREF_44), [48](#_ENREF_48), [50](#_ENREF_50), [53](#_ENREF_53), [54](#_ENREF_54), [68](#_ENREF_68), [73](#_ENREF_73), [75](#_ENREF_75), [108](#_ENREF_108), [117](#_ENREF_117), [120](#_ENREF_120), [122-126](#_ENREF_122), [130](#_ENREF_130)] |
| **Capability - Empowerment** | **10**  [[3](#_ENREF_3), [27](#_ENREF_27), [29](#_ENREF_29), [34](#_ENREF_34), [72](#_ENREF_72), [89](#_ENREF_89), [91](#_ENREF_91), [94](#_ENREF_94), [99](#_ENREF_99), [107](#_ENREF_107)] | **4 (40%)**  [[27](#_ENREF_27), [29](#_ENREF_29), [34](#_ENREF_34), [107](#_ENREF_107)] |
| **COMMUNITY BASED CARE** | | |
| **Capability** | **28**  [[10](#_ENREF_10), [12-14](#_ENREF_12), [31](#_ENREF_31), [36](#_ENREF_36), [41](#_ENREF_41), [42](#_ENREF_42), [44](#_ENREF_44), [46](#_ENREF_46), [49](#_ENREF_49), [51](#_ENREF_51), [52](#_ENREF_52), [55](#_ENREF_55), [62](#_ENREF_62), [63](#_ENREF_63), [65](#_ENREF_65), [66](#_ENREF_66), [69](#_ENREF_69), [71](#_ENREF_71), [73](#_ENREF_73), [76](#_ENREF_76), [83](#_ENREF_83), [93](#_ENREF_93), [100](#_ENREF_100), [114](#_ENREF_114), [115](#_ENREF_115), [130](#_ENREF_130)] | **18 (64%)**  [[31](#_ENREF_31), [36](#_ENREF_36), [41](#_ENREF_41), [42](#_ENREF_42), [44](#_ENREF_44), [46](#_ENREF_46), [49](#_ENREF_49), [51](#_ENREF_51), [52](#_ENREF_52), [63](#_ENREF_63), [65](#_ENREF_65), [66](#_ENREF_66), [69](#_ENREF_69), [71](#_ENREF_71), [73](#_ENREF_73), [114](#_ENREF_114), [115](#_ENREF_115), [130](#_ENREF_130)] |
| **Capability - Capacity to plan** | **17**  [[12-14](#_ENREF_12), [36](#_ENREF_36), [41](#_ENREF_41), [42](#_ENREF_42), [44](#_ENREF_44), [51](#_ENREF_51), [52](#_ENREF_52), [55](#_ENREF_55), [63](#_ENREF_63), [65](#_ENREF_65), [71](#_ENREF_71), [73](#_ENREF_73), [83](#_ENREF_83), [100](#_ENREF_100), [130](#_ENREF_130)] | **11 (65%)**  [[36](#_ENREF_36), [41](#_ENREF_41), [42](#_ENREF_42), [44](#_ENREF_44), [51](#_ENREF_51), [52](#_ENREF_52), [63](#_ENREF_63), [65](#_ENREF_65), [71](#_ENREF_71), [73](#_ENREF_73), [130](#_ENREF_130)] |
| **Capability - Enhance knowledge and skills of individual** | **24**  [[12-14](#_ENREF_12), [31](#_ENREF_31), [36](#_ENREF_36), [41](#_ENREF_41), [42](#_ENREF_42), [44](#_ENREF_44), [46](#_ENREF_46), [49](#_ENREF_49), [51](#_ENREF_51), [52](#_ENREF_52), [55](#_ENREF_55), [62](#_ENREF_62), [63](#_ENREF_63), [65](#_ENREF_65), [66](#_ENREF_66), [71](#_ENREF_71), [73](#_ENREF_73), [83](#_ENREF_83), [93](#_ENREF_93), [114](#_ENREF_114), [115](#_ENREF_115), [130](#_ENREF_130)] | **17 (71%)**  [[31](#_ENREF_31), [36](#_ENREF_36), [41](#_ENREF_41), [42](#_ENREF_42), [44](#_ENREF_44), [46](#_ENREF_46), [49](#_ENREF_49), [51](#_ENREF_51), [52](#_ENREF_52), [63](#_ENREF_63), [65](#_ENREF_65), [66](#_ENREF_66), [71](#_ENREF_71), [73](#_ENREF_73), [114](#_ENREF_114), [115](#_ENREF_115), [130](#_ENREF_130)] |
| **WORKPLACE** | | |
| **Capability - Capacity to plan** | **14**  [[15](#_ENREF_15), [23](#_ENREF_23), [24](#_ENREF_24), [26](#_ENREF_26), [28](#_ENREF_28), [32](#_ENREF_32), [57](#_ENREF_57), [71](#_ENREF_71), [96](#_ENREF_96), [98](#_ENREF_98), [110](#_ENREF_110), [116](#_ENREF_116), [119](#_ENREF_119), [126](#_ENREF_126)] | **10 (71%)**  [[23](#_ENREF_23), [24](#_ENREF_24), [26](#_ENREF_26), [28](#_ENREF_28), [32](#_ENREF_32), [71](#_ENREF_71), [110](#_ENREF_110), [116](#_ENREF_116), [119](#_ENREF_119), [126](#_ENREF_126)] |
| **SCHOOLS** | | |
| **Capability** | **15**  [[6](#_ENREF_6), [35](#_ENREF_35), [41](#_ENREF_41), [43](#_ENREF_43), [44](#_ENREF_44), [61](#_ENREF_61), [62](#_ENREF_62), [66](#_ENREF_66), [67](#_ENREF_67), [69](#_ENREF_69), [70](#_ENREF_70), [82](#_ENREF_82), [87](#_ENREF_87), [114](#_ENREF_114), [132](#_ENREF_132)] | **10 (67%)**  [[35](#_ENREF_35), [41](#_ENREF_41), [43](#_ENREF_43), [44](#_ENREF_44), [66](#_ENREF_66), [67](#_ENREF_67), [69](#_ENREF_69), [70](#_ENREF_70), [114](#_ENREF_114), [132](#_ENREF_132)] |
| **Capability – Capacity to Plan** | **7**  [[6](#_ENREF_6), [41](#_ENREF_41), [44](#_ENREF_44), [61](#_ENREF_61), [67](#_ENREF_67), [82](#_ENREF_82), [132](#_ENREF_132)] | **4 (57%)**  [[41](#_ENREF_41), [44](#_ENREF_44), [67](#_ENREF_67), [132](#_ENREF_132)] |
| **Capability – Enhance knowledge and skills of individual** | **15**  [[6](#_ENREF_6), [35](#_ENREF_35), [41](#_ENREF_41), [43](#_ENREF_43), [44](#_ENREF_44), [62](#_ENREF_62), [66](#_ENREF_66), [67](#_ENREF_67), [69](#_ENREF_69), [70](#_ENREF_70), [82](#_ENREF_82), [87](#_ENREF_87), [114](#_ENREF_114), [132](#_ENREF_132), [133](#_ENREF_133)] | **10 (67%)**  [[35](#_ENREF_35), [41](#_ENREF_41), [43](#_ENREF_43), [44](#_ENREF_44), [66](#_ENREF_66), [67](#_ENREF_67), [69](#_ENREF_69), [70](#_ENREF_70), [114](#_ENREF_114), [132](#_ENREF_132)] |
| **TARGET POPULATION** | | |
| **SECONDARY PREVENTION** | | |
| **Capability – Capacity to Plan** | **36**  [[12-14](#_ENREF_12), [16](#_ENREF_16), [18](#_ENREF_18), [21](#_ENREF_21), [25](#_ENREF_25), [32](#_ENREF_32), [34](#_ENREF_34), [37-39](#_ENREF_37), [41](#_ENREF_41), [50](#_ENREF_50), [51](#_ENREF_51), [53](#_ENREF_53), [54](#_ENREF_54), [68](#_ENREF_68), [72](#_ENREF_72), [75](#_ENREF_75), [77](#_ENREF_77), [78](#_ENREF_78), [80](#_ENREF_80), [83](#_ENREF_83), [89](#_ENREF_89), [90](#_ENREF_90), [92](#_ENREF_92), [99](#_ENREF_99), [101](#_ENREF_101), [102](#_ENREF_102), [108](#_ENREF_108), [122](#_ENREF_122), [123](#_ENREF_123), [125](#_ENREF_125), [129](#_ENREF_129), [130](#_ENREF_130)] | **19 (53%)**  [[25](#_ENREF_25), [32](#_ENREF_32), [34](#_ENREF_34), [37-39](#_ENREF_37), [41](#_ENREF_41), [50](#_ENREF_50), [51](#_ENREF_51), [53](#_ENREF_53), [54](#_ENREF_54), [68](#_ENREF_68), [75](#_ENREF_75), [108](#_ENREF_108), [122](#_ENREF_122), [123](#_ENREF_123), [125](#_ENREF_125), [129](#_ENREF_129), [130](#_ENREF_130)] |
| **Capability – Empowerment** | **8**  [[16](#_ENREF_16), [27](#_ENREF_27), [32](#_ENREF_32), [34](#_ENREF_34), [72](#_ENREF_72), [89](#_ENREF_89), [91](#_ENREF_91), [99](#_ENREF_99)] | **3 (38%)**  [[27](#_ENREF_27), [32](#_ENREF_32), [34](#_ENREF_34)] |
| **PROFESSION** | | |
| **MULTI-DISCIPLINARY TEAM** | | |
| **Capability – Capacity to Plan** | **30**  [[3](#_ENREF_3), [4](#_ENREF_4), [16](#_ENREF_16), [32](#_ENREF_32), [38](#_ENREF_38), [39](#_ENREF_39), [44](#_ENREF_44), [48](#_ENREF_48), [51-57](#_ENREF_51), [59](#_ENREF_59), [68](#_ENREF_68), [71](#_ENREF_71), [73](#_ENREF_73), [83](#_ENREF_83), [84](#_ENREF_84), [92](#_ENREF_92), [99](#_ENREF_99), [108](#_ENREF_108), [119](#_ENREF_119), [122](#_ENREF_122), [124](#_ENREF_124), [126](#_ENREF_126), [129](#_ENREF_129), [130](#_ENREF_130)] | **19 (63%)**  [[32](#_ENREF_32), [38](#_ENREF_38), [39](#_ENREF_39), [44](#_ENREF_44), [48](#_ENREF_48), [51-54](#_ENREF_51), [68](#_ENREF_68), [71](#_ENREF_71), [73](#_ENREF_73), [108](#_ENREF_108), [119](#_ENREF_119), [122](#_ENREF_122), [124](#_ENREF_124), [126](#_ENREF_126), [129](#_ENREF_129), [130](#_ENREF_130)] |
| **Capability – Enhance knowledge and skills of individual** | **41**  [[3](#_ENREF_3), [4](#_ENREF_4), [16](#_ENREF_16), [17](#_ENREF_17), [19](#_ENREF_19), [31](#_ENREF_31), [32](#_ENREF_32), [38-40](#_ENREF_38), [44](#_ENREF_44), [48](#_ENREF_48), [51-57](#_ENREF_51), [59](#_ENREF_59), [61](#_ENREF_61), [64](#_ENREF_64), [68](#_ENREF_68), [71](#_ENREF_71), [73](#_ENREF_73), [81](#_ENREF_81), [83](#_ENREF_83), [84](#_ENREF_84), [88](#_ENREF_88), [92](#_ENREF_92), [107-109](#_ENREF_107), [119](#_ENREF_119), [122](#_ENREF_122), [124](#_ENREF_124), [126](#_ENREF_126), [127](#_ENREF_127), [129](#_ENREF_129), [130](#_ENREF_130)] | **26 (63%)**  [[31](#_ENREF_31), [32](#_ENREF_32), [38-40](#_ENREF_38), [44](#_ENREF_44), [48](#_ENREF_48), [51-54](#_ENREF_51), [64](#_ENREF_64), [68](#_ENREF_68), [71](#_ENREF_71), [73](#_ENREF_73), [107-109](#_ENREF_107), [115](#_ENREF_115), [119](#_ENREF_119), [122](#_ENREF_122), [124](#_ENREF_124), [126](#_ENREF_126), [127](#_ENREF_127), [129](#_ENREF_129), [130](#_ENREF_130)] |
| **TYPE OF MODIFIABLE RISK BEHAVIOUR INTERVENTION ADDRESSED^b^** | | |
| **ALCOHOL** | | |
| **Capability - Beliefs about interventions** | **4**  [[34](#_ENREF_34), [51](#_ENREF_51), [81](#_ENREF_81), [82](#_ENREF_82)] | **2 (50%)**  [[34](#_ENREF_34), [51](#_ENREF_51)] |
| **Capability - Empowerment** | **12**  [[23](#_ENREF_23), [24](#_ENREF_24), [29](#_ENREF_29), [32](#_ENREF_32), [34](#_ENREF_34), [35](#_ENREF_35), [47](#_ENREF_47), [72](#_ENREF_72), [82](#_ENREF_82), [91](#_ENREF_91), [93](#_ENREF_93), [103](#_ENREF_103)] | **7 (58%)**  [[23](#_ENREF_23), [24](#_ENREF_24), [29](#_ENREF_29), [32](#_ENREF_32), [34](#_ENREF_34), [35](#_ENREF_35), [47](#_ENREF_47)] |
| **DIET** | | |
| **Capability - Beliefs about interventions** | **6**  [[22](#_ENREF_22), [34](#_ENREF_34), [42](#_ENREF_42), [73](#_ENREF_73), [81](#_ENREF_81), [82](#_ENREF_82)] | **3 (50%)**  [[34](#_ENREF_34), [42](#_ENREF_42), [73](#_ENREF_73)] |
| **PHYSICAL ACTIVITY** | | |
| **Capability - Beliefs about interventions** | **7**  [[22](#_ENREF_22), [34](#_ENREF_34), [42](#_ENREF_42), [51](#_ENREF_51), [73](#_ENREF_73), [81](#_ENREF_81), [82](#_ENREF_82)] | **4 (57%)**  [[34](#_ENREF_34), [42](#_ENREF_42), [51](#_ENREF_51), [73](#_ENREF_73)] |
| **Capability – Empowerment** | **21**  [[3](#_ENREF_3), [10](#_ENREF_10), [16](#_ENREF_16), [23](#_ENREF_23), [24](#_ENREF_24), [27](#_ENREF_27), [29](#_ENREF_29), [32](#_ENREF_32), [34](#_ENREF_34), [35](#_ENREF_35), [45](#_ENREF_45), [47](#_ENREF_47), [72](#_ENREF_72), [82](#_ENREF_82), [89](#_ENREF_89), [91](#_ENREF_91), [93](#_ENREF_93), [94](#_ENREF_94), [96](#_ENREF_96), [99](#_ENREF_99), [107](#_ENREF_107)] | **10 (48%)**  [[23](#_ENREF_23), [24](#_ENREF_24), [27](#_ENREF_27), [29](#_ENREF_29), [32](#_ENREF_32), [34](#_ENREF_34), [35](#_ENREF_35), [45](#_ENREF_45), [47](#_ENREF_47), [107](#_ENREF_107)] |
| **STRESS** | | |
| **Capability - Capacity to plan** | **27**  [[13-15](#_ENREF_13), [23](#_ENREF_23), [24](#_ENREF_24), [26](#_ENREF_26), [28](#_ENREF_28), [32](#_ENREF_32), [38](#_ENREF_38), [41](#_ENREF_41), [42](#_ENREF_42), [44](#_ENREF_44), [47](#_ENREF_47), [50](#_ENREF_50), [54](#_ENREF_54), [55](#_ENREF_55), [63](#_ENREF_63), [72](#_ENREF_72), [75](#_ENREF_75), [78](#_ENREF_78), [95](#_ENREF_95), [96](#_ENREF_96), [98](#_ENREF_98), [101](#_ENREF_101), [116](#_ENREF_116), [119](#_ENREF_119), [123](#_ENREF_123)] | **17 (63%)**  [[23](#_ENREF_23), [24](#_ENREF_24), [26](#_ENREF_26), [28](#_ENREF_28), [32](#_ENREF_32), [38](#_ENREF_38), [41](#_ENREF_41), [42](#_ENREF_42), [44](#_ENREF_44), [47](#_ENREF_47), [50](#_ENREF_50), [54](#_ENREF_54), [63](#_ENREF_63), [75](#_ENREF_75), [116](#_ENREF_116), [119](#_ENREF_119), [123](#_ENREF_123)] |
| **Capability - Empowerment** | **6**  [[23](#_ENREF_23), [24](#_ENREF_24), [32](#_ENREF_32), [47](#_ENREF_47), [72](#_ENREF_72), [96](#_ENREF_96)] | **4 (67%)**  [[23](#_ENREF_23), [24](#_ENREF_24), [32](#_ENREF_32), [47](#_ENREF_47)] |
| **NUMBER OF MODIFIABLE RISK BEHAVIOURS ADDRESSED** | | |
| **3 BEHAVIOURS** | | |
| **Capability - Capacity to plan** | **42**  [[3](#_ENREF_3), [4](#_ENREF_4), [16](#_ENREF_16), [18](#_ENREF_18), [22](#_ENREF_22), [36](#_ENREF_36), [37](#_ENREF_37), [39](#_ENREF_39), [45](#_ENREF_45), [51-53](#_ENREF_51), [56](#_ENREF_56), [57](#_ENREF_57), [60](#_ENREF_60), [61](#_ENREF_61), [65](#_ENREF_65), [68](#_ENREF_68), [71](#_ENREF_71), [73](#_ENREF_73), [75](#_ENREF_75), [77](#_ENREF_77), [79](#_ENREF_79), [80](#_ENREF_80), [83](#_ENREF_83), [89](#_ENREF_89), [90](#_ENREF_90), [92](#_ENREF_92), [94](#_ENREF_94), [95](#_ENREF_95), [98-100](#_ENREF_98), [103](#_ENREF_103), [106](#_ENREF_106), [110](#_ENREF_110), [118](#_ENREF_118), [120](#_ENREF_120), [122-124](#_ENREF_122), [126](#_ENREF_126)] | **20 (48%)**  [[36](#_ENREF_36), [37](#_ENREF_37), [39](#_ENREF_39), [45](#_ENREF_45), [51-53](#_ENREF_51), [65](#_ENREF_65), [68](#_ENREF_68), [71](#_ENREF_71), [73](#_ENREF_73), [75](#_ENREF_75), [106](#_ENREF_106), [110](#_ENREF_110), [118](#_ENREF_118), [120](#_ENREF_120), [122-124](#_ENREF_122), [126](#_ENREF_126)] |
| **Capability - Empowerment** | **11**  [[3](#_ENREF_3), [10](#_ENREF_10), [16](#_ENREF_16), [27](#_ENREF_27), [45](#_ENREF_45), [89](#_ENREF_89), [91](#_ENREF_91), [94](#_ENREF_94), [99](#_ENREF_99), [103](#_ENREF_103), [107](#_ENREF_107)] | **3 (27%)**  [[27](#_ENREF_27), [45](#_ENREF_45), [107](#_ENREF_107)] |
| **4 BEHAVIOURS** | | |
| **Capability – Beliefs about intervention** | **4**  [[34](#_ENREF_34), [42](#_ENREF_42), [81](#_ENREF_81), [82](#_ENREF_82)] | **2 (50%)**  [[34](#_ENREF_34), [42](#_ENREF_42)] |
| **5 BEHAVIOURS** | | |
| **Capability** | **13**  [[5](#_ENREF_5), [9](#_ENREF_9), [14](#_ENREF_14), [23](#_ENREF_23), [24](#_ENREF_24), [32](#_ENREF_32), [47](#_ENREF_47), [55](#_ENREF_55), [78](#_ENREF_78), [85](#_ENREF_85), [96](#_ENREF_96), [116](#_ENREF_116), [119](#_ENREF_119)] | **6 (46%)**  [[23](#_ENREF_23), [24](#_ENREF_24), [32](#_ENREF_32), [47](#_ENREF_47), [116](#_ENREF_116), [119](#_ENREF_119)] |
| **Capability – Capacity to Plan** | **10**  [[14](#_ENREF_14), [23](#_ENREF_23), [24](#_ENREF_24), [32](#_ENREF_32), [47](#_ENREF_47), [55](#_ENREF_55), [78](#_ENREF_78), [96](#_ENREF_96), [116](#_ENREF_116), [119](#_ENREF_119)] | **6 (60%)**  [[23](#_ENREF_23), [24](#_ENREF_24), [32](#_ENREF_32), [47](#_ENREF_47), [116](#_ENREF_116), [119](#_ENREF_119)] |
| **Capability – Empowerment** | **5**  [[23](#_ENREF_23), [24](#_ENREF_24), [32](#_ENREF_32), [47](#_ENREF_47), [96](#_ENREF_96)] | **4 (80%)**  [[23](#_ENREF_23), [24](#_ENREF_24), [32](#_ENREF_32), [47](#_ENREF_47)] |
| **Capability - Enhance knowledge** | **12**  [[5](#_ENREF_5), [9](#_ENREF_9), [14](#_ENREF_14), [23](#_ENREF_23), [32](#_ENREF_32), [47](#_ENREF_47), [55](#_ENREF_55), [78](#_ENREF_78), [85](#_ENREF_85), [96](#_ENREF_96), [116](#_ENREF_116), [119](#_ENREF_119)] | **5 (42%)**  [[23](#_ENREF_23), [32](#_ENREF_32), [47](#_ENREF_47), [116](#_ENREF_116), [119](#_ENREF_119)] |

## ^a^ The mechanisms within these intervention may not be exclusively for addressing smoking behaviour. It could be part of the overall intervention or for other risk behaviours within that intervention.

## ^b^ This category examines interventions that include the specific risk behaviour (i.e. alcohol) as one of the targeted behaviours.

**Table 7:** *All Interventions That Reported Using Motivation as One of the Mechanisms.*

| **Mechanism^a^** | **Total number of interventions using this C-M-O** | **Number of interventions using this C-M-O that report improvement in smoking cessation outcome.** |
| --- | --- | --- |
| **Motivation** | **83**  [[1-3](#_ENREF_1), [6-9](#_ENREF_6), [13](#_ENREF_13), [15](#_ENREF_15), [17](#_ENREF_17), [18](#_ENREF_18), [20](#_ENREF_20), [22-26](#_ENREF_22), [28](#_ENREF_28), [29](#_ENREF_29), [31](#_ENREF_31), [33](#_ENREF_33), [35](#_ENREF_35), [38](#_ENREF_38), [42](#_ENREF_42), [44](#_ENREF_44), [47](#_ENREF_47), [48](#_ENREF_48), [50-54](#_ENREF_50), [57-63](#_ENREF_57), [70-73](#_ENREF_70), [75](#_ENREF_75), [77](#_ENREF_77), [79](#_ENREF_79), [81-84](#_ENREF_81), [86](#_ENREF_86), [88-90](#_ENREF_88), [92](#_ENREF_92), [94-96](#_ENREF_94), [98-101](#_ENREF_98), [104](#_ENREF_104), [105](#_ENREF_105), [111](#_ENREF_111), [112](#_ENREF_112), [114](#_ENREF_114), [116](#_ENREF_116), [118-122](#_ENREF_118), [126](#_ENREF_126), [128](#_ENREF_128), [131](#_ENREF_131), [136-141](#_ENREF_136)] | **39 (47%)**  [[23-26](#_ENREF_23), [28](#_ENREF_28), [29](#_ENREF_29), [31-33](#_ENREF_31), [35](#_ENREF_35), [38](#_ENREF_38), [42](#_ENREF_42), [44](#_ENREF_44), [47](#_ENREF_47), [48](#_ENREF_48), [50-54](#_ENREF_50), [63](#_ENREF_63), [70](#_ENREF_70), [71](#_ENREF_71), [73](#_ENREF_73), [75](#_ENREF_75), [111](#_ENREF_111), [112](#_ENREF_112), [114](#_ENREF_114), [116](#_ENREF_116), [118-122](#_ENREF_118), [126](#_ENREF_126), [128](#_ENREF_128), [131](#_ENREF_131), [140](#_ENREF_140), [141](#_ENREF_141)] |
| **CONTEXT** | | |
| **CLINICAL SETTING** | | |
| **Motivation** | **38**  [[3](#_ENREF_3), [7](#_ENREF_7), [18](#_ENREF_18), [22](#_ENREF_22), [29](#_ENREF_29), [33](#_ENREF_33), [44](#_ENREF_44), [48](#_ENREF_48), [50](#_ENREF_50), [53](#_ENREF_53), [54](#_ENREF_54), [58](#_ENREF_58), [59](#_ENREF_59), [72](#_ENREF_72), [73](#_ENREF_73), [75](#_ENREF_75), [77](#_ENREF_77), [79](#_ENREF_79), [81](#_ENREF_81), [84](#_ENREF_84), [86](#_ENREF_86), [89](#_ENREF_89), [90](#_ENREF_90), [92](#_ENREF_92), [94](#_ENREF_94), [99-101](#_ENREF_99), [105](#_ENREF_105), [111](#_ENREF_111), [120](#_ENREF_120), [122](#_ENREF_122), [126](#_ENREF_126), [136](#_ENREF_136), [137](#_ENREF_137), [139-141](#_ENREF_139)] | **15 (39%)**  [[29](#_ENREF_29), [33](#_ENREF_33), [44](#_ENREF_44), [48](#_ENREF_48), [50](#_ENREF_50), [53](#_ENREF_53), [54](#_ENREF_54), [73](#_ENREF_73), [75](#_ENREF_75), [111](#_ENREF_111), [120](#_ENREF_120), [122](#_ENREF_122), [126](#_ENREF_126), [140](#_ENREF_140), [141](#_ENREF_141)] |
| **COMMUNITY BASED CARE** | | |
| **Motivation** | **14**  [[13](#_ENREF_13), [20](#_ENREF_20), [31](#_ENREF_31), [42](#_ENREF_42), [44](#_ENREF_44), [51](#_ENREF_51), [52](#_ENREF_52), [62](#_ENREF_62), [63](#_ENREF_63), [71](#_ENREF_71), [73](#_ENREF_73), [83](#_ENREF_83), [100](#_ENREF_100), [114](#_ENREF_114)] | **9 (64%)**  [[31](#_ENREF_31), [42](#_ENREF_42), [44](#_ENREF_44), [51](#_ENREF_51), [52](#_ENREF_52), [63](#_ENREF_63), [71](#_ENREF_71), [73](#_ENREF_73), [114](#_ENREF_114)] |
| **SCHOOL** | | |
| **Motivation** | **8**  [[6](#_ENREF_6), [35](#_ENREF_35), [44](#_ENREF_44), [61](#_ENREF_61), [62](#_ENREF_62), [70](#_ENREF_70), [82](#_ENREF_82), [114](#_ENREF_114)] | **4 (50%)**  [[35](#_ENREF_35), [44](#_ENREF_44), [70](#_ENREF_70), [114](#_ENREF_114)] |
| **TYPE OF MODIFIABLE RISK BEHAVIOUR INTERVENTION ADDRESSED^b^** | | |
| **STRESS** | | |
| **Motivation** | **27**  [[8](#_ENREF_8), [9](#_ENREF_9), [13](#_ENREF_13), [15](#_ENREF_15), [23](#_ENREF_23), [24](#_ENREF_24), [26](#_ENREF_26), [28](#_ENREF_28), [31](#_ENREF_31), [32](#_ENREF_32), [38](#_ENREF_38), [42](#_ENREF_42), [44](#_ENREF_44), [47](#_ENREF_47), [50](#_ENREF_50), [54](#_ENREF_54), [63](#_ENREF_63), [72](#_ENREF_72), [75](#_ENREF_75), [95](#_ENREF_95), [96](#_ENREF_96), [98](#_ENREF_98), [101](#_ENREF_101), [104](#_ENREF_104), [116](#_ENREF_116), [119](#_ENREF_119), [131](#_ENREF_131)] | **17 (63%)**  [[23](#_ENREF_23), [24](#_ENREF_24), [26](#_ENREF_26), [28](#_ENREF_28), [31](#_ENREF_31), [32](#_ENREF_32), [38](#_ENREF_38), [42](#_ENREF_42), [44](#_ENREF_44), [47](#_ENREF_47), [50](#_ENREF_50), [54](#_ENREF_54), [63](#_ENREF_63), [75](#_ENREF_75), [116](#_ENREF_116), [119](#_ENREF_119), [131](#_ENREF_131)] |
| **NUMBER OF MODIFIABLE RISK BEHAVIOURS ADDRESSED** | | |
| **3 BEHAVIOURS** | | |
| **Motivation** | **38**  [[3](#_ENREF_3), [17](#_ENREF_17), [18](#_ENREF_18), [20](#_ENREF_20), [22](#_ENREF_22), [33](#_ENREF_33), [51-53](#_ENREF_51), [57](#_ENREF_57), [58](#_ENREF_58), [60-62](#_ENREF_60), [70](#_ENREF_70), [71](#_ENREF_71), [73](#_ENREF_73), [75](#_ENREF_75), [77](#_ENREF_77), [79](#_ENREF_79), [83](#_ENREF_83), [89](#_ENREF_89), [90](#_ENREF_90), [92](#_ENREF_92), [94](#_ENREF_94), [95](#_ENREF_95), [98-100](#_ENREF_98), [114](#_ENREF_114), [118](#_ENREF_118), [120-122](#_ENREF_120), [126](#_ENREF_126), [136](#_ENREF_136), [139](#_ENREF_139), [140](#_ENREF_140)] | **15 (39%)**  [[33](#_ENREF_33), [51-53](#_ENREF_51), [70](#_ENREF_70), [71](#_ENREF_71), [73](#_ENREF_73), [75](#_ENREF_75), [114](#_ENREF_114), [118](#_ENREF_118), [120-122](#_ENREF_120), [126](#_ENREF_126), [140](#_ENREF_140)] |

## ^a^ The mechanisms within these intervention may not be exclusively for addressing smoking behaviour. It could be part of the overall intervention or for other risk behaviours within that intervention.

## ^b^ This category examines interventions that include the specific risk behaviour (i.e. alcohol) as one of the targeted behaviours.

**References**

1. Salisbury C, O'Cathain A, Thomas C, Edwards L, Gaunt D, Dixon P, et al. Telehealth for patients at high risk of cardiovascular disease: pragmatic randomised controlled trial. BMJ. 2016;353:i2647.

2. Wierenga D, Engbers LH, Van Empelen P, De Moes KJ, Wittink H, Grundemann R, et al. The implementation of multiple lifestyle interventions in two organizations: a process evaluation. J Occup Environ Med. 2014;56(11):1195-206.

3. Speyer H, Nørgaard HCB, Birk M, Karlsen M, Jakobsen AS, Pedersen K, et al. The CHANGE trial: no superiority of lifestyle coaching plus care coordination plus treatment as usual compared to treatment as usual alone in reducing risk of cardiovascular disease in adults with schizophrenia spectrum disorders and abdominal obesity. World Psychiatry. 2016;15(2):155.

4. Griffin SJ, Simmons RK, Prevost AT, Williams KM, Hardeman W, Sutton S, et al. Multiple behaviour change intervention and outcomes in recently diagnosed type 2 diabetes: the ADDITION-Plus randomised controlled trial. Diabetologia. 2014;57(7):1308-19.

5. Abbas SZ, Pollard TM, Wynn P, Learmonth A, Joyce K, Bambra C. The effectiveness of using the workplace to identify and address modifiable health risk factors in deprived populations. Occup Environ Med. 2015;72(9):664-9.

6. Lana A, Faya-Ornia G, Lopez ML. Impact of a web-based intervention supplemented with text messages to improve cancer prevention behaviors among adolescents: results from a randomized controlled trial. Prev Med. 2014;59:54-9.

7. Vrdoljak D, Markovic BB, Puljak L, Lalic DI, Kranjcevic K, Vucak J. Lifestyle intervention in general practice for physical activity, smoking, alcohol consumption and diet in elderly: a randomized controlled trial. Arch Gerontol Geriatr. 2014;58(1):160-9.

8. Blake H, Zhou D, Batt ME. Five-year workplace wellness intervention in the NHS. Perspect Public Health. 2013;133(5):262-71.

9. Huang JJ, Lin HS, Yen M, Kan WM, Lee BO, Chen CH. Effects of a workplace multiple cardiovascular disease risks reduction program. Asian Nurs Res (Korean Soc Nurs Sci). 2013;7(2):74-82.

10. Fahs PS, Pribulick M, Williams IC, James GD, Rovnyak V, Seibold-Simpson SM. Promoting heart health in rural women. J Rural Health. 2013;29(3):248-57.

11. Lindsay S, Smith S, Bellaby P, Baker R. The health impact of an online heart disease support group: a comparison of moderated versus unmoderated support. Health Educ Res. 2009;24(4):646-54.

12. McNamara KP, O'Reilly SL, George J, Peterson GM, Jackson SL, Duncan G, et al. Intervention fidelity for a complex behaviour change intervention in community pharmacy addressing cardiovascular disease risk. Health Educ Res. 2015;30(6):897-909.

13. Toobert DJ, Strycker LA, Barrera M, Jr., Osuna D, King DK, Glasgow RE. Outcomes from a multiple risk factor diabetes self-management trial for Latinas: inverted exclamation markViva Bien! Ann Behav Med. 2011;41(3):310-23.

14. Han HR, Kim J, Kim KB, Jeong S, Levine D, Li C, et al. Implementation and success of nurse telephone counseling in linguistically isolated Korean American patients with high blood pressure. Patient Educ Couns. 2010;80(1):130-4.

15. Moy F, Sallam AA, Wong M. The results of a worksite health promotion programme in Kuala Lumpur, Malaysia. Health Promot Int. 2006;21(4):301-10.

16. Bradshaw T, Lovell K, Bee P, Campbell M. The development and evaluation of a complex health education intervention for adults with a diagnosis of schizophrenia. J Psychiatr Ment Health Nurs. 2010;17(6):473-86.

17. Milani RV, Lavie CJ. Impact of worksite wellness intervention on cardiac risk factors and one-year health care costs. Am J Cardiol. 2009;104(10):1389-92.

18. Jiang X, Sit JW, Wong TK. A nurse‐led cardiac rehabilitation programme improves health behaviours and cardiac physiological risk parameters: evidence from Chengdu, China. Journal of clinical nursing. 2007;16(10):1886-97.

19. Bhiri S, Maatoug J, Zammit N, Msakni Z, Harrabi I, Amimi S, et al. A 3-Year Workplace-Based Intervention Program to Control Noncommunicable Disease Risk Factors in Sousse, Tunisia. J Occup Environ Med. 2015;57(7):e72-7.

20. Kottke TE, Thomas RJ, Lopez-Jimenez F, Brekke LN, Brekke MJ, Aase LA, et al. CardioVision 2020: program acceptance and progress after 4 years. Am J Prev Med. 2006;30(2):137-43.

21. Wan LH, Zhang XP, Mo MM, Xiong XN, Ou CL, You LM, et al. Effectiveness of Goal-Setting Telephone Follow-Up on Health Behaviors of Patients with Ischemic Stroke: A Randomized Controlled Trial. J Stroke Cerebrovasc Dis. 2016;25(9):2259-70.

22. Kanera IM, Bolman CA, Willems RA, Mesters I, Lechner L. Lifestyle-related effects of the web-based Kanker Nazorg Wijzer (Cancer Aftercare Guide) intervention for cancer survivors: a randomized controlled trial. J Cancer Surviv. 2016;10(5):883-97.

23. Byrne DW, Goetzel RZ, McGown PW, Holmes MC, Beckowski MS, Tabrizi MJ, et al. Seven-year trends in employee health habits from a comprehensive workplace health promotion program at Vanderbilt University. J Occup Environ Med. 2011;53(12):1372-81.

24. Henke RM, Goetzel RZ, McHugh J, Isaac F. Recent experience in health promotion at Johnson & Johnson: lower health spending, strong return on investment. Health Aff (Millwood). 2011;30(3):490-9.

25. Zhou B, Chen K, Yu Y, Wang H, Zhang S, Zheng W. Individualized Health Intervention: Behavioral Change and Quality of Life in an Older Rural Chinese Population. Educational Gerontology. 2010;36(10-11):919-39.

26. Long DA, Sheehan P. A case study of population health improvement at a Midwest regional hospital employer. Popul Health Manag. 2010;13(3):163-73.

27. Shalaeva EV, Saner H, Janabaev BB, Shalaeva AV. Tenfold risk increase of major cardiovascular events after high limb amputation with non-compliance for secondary prevention measures. Eur J Prev Cardiol. 2017;24(7):708-16.

28. Kuehl KS, Elliot DL, MacKinnon DP, O'Rourke HP, DeFrancesco C, Miocevic M, et al. The SHIELD (Safety & Health Improvement: Enhancing Law Enforcement Departments) Study: Mixed Methods Longitudinal Findings. J Occup Environ Med. 2016;58(5):492-8.

29. Kadda O, Manginas A, Stavridis G, Balanos D, Kotiou M, Panagiotakos DB. Gender Analysis in the Outcomes of a Lifestyle Intervention Among Patients Who Had an Open Heart Surgery. Angiology. 2016;67(1):66-74.

30. Jelinek MV, Santamaria JD, Best JD, Thompson DR, Tonkin AM, Vale MJ. Reversing social disadvantage in secondary prevention of coronary heart disease. Int J Cardiol. 2014;171(3):346-50.

31. Gibson I, Flaherty G, Cormican S, Jones J, Kerins C, Walsh AM, et al. Translating guidelines to practice: findings from a multidisciplinary preventive cardiology programme in the west of Ireland. Eur J Prev Cardiol. 2014;21(3):366-76.

32. Loeppke R, Edington D, Bender J, Reynolds A. The association of technology in a workplace wellness program with health risk factor reduction. J Occup Environ Med. 2013;55(3):259-64.

33. Monteagudo M, Rodriguez-Blanco T, Llagostera M, Valero C, Bayona X, Granollers S, et al. Effect of health professional education on outcomes of chronic obstructive pulmonary disease in primary care: a non-randomized clinical trial. Respirology. 2013;18(4):718-27.

34. Xavier D, Gupta R, Kamath D, Sigamani A, Devereaux PJ, George N, et al. Community health worker-based intervention for adherence to drugs and lifestyle change after acute coronary syndrome: a multicentre, open, randomised controlled trial. The Lancet Diabetes & Endocrinology. 2016;4(3):244-53.

35. Busch V, De Leeuw RJ, Schrijvers AJ. Results of a multibehavioral health-promoting school pilot intervention in a Dutch secondary school. J Adolesc Health. 2013;52(4):400-6.

36. Richardson G, van Woerden HC, Morgan L, Edwards R, Harries M, Hancock E, et al. Healthy hearts--a community-based primary prevention programme to reduce coronary heart disease. BMC Cardiovasc Disord. 2008;8:18.

37. Smith S, Yeomans D, Bushe CJ, Eriksson C, Harrison T, Holmes R, et al. A well-being programme in severe mental illness. Reducing risk for physical ill-health: a post-programme service evaluation at 2 years. Eur Psychiatry. 2007;22(7):413-8.

38. Park AH, Lee SJ, Oh SJ. The effects of a smoking cessation programme on health-promoting lifestyles and smoking cessation in smokers who had undergone percutaneous coronary intervention. Int J Nurs Pract. 2015;21(2):107-17.

39. Kim JY, Wineinger NE, Steinhubl SR. The Influence of Wireless Self-Monitoring Program on the Relationship Between Patient Activation and Health Behaviors, Medication Adherence, and Blood Pressure Levels in Hypertensive Patients: A Substudy of a Randomized Controlled Trial. J Med Internet Res. 2016;18(6):e116.

40. Fernald DH, Dickinson LM, Froshaug DB, Balasubramanian BA, Holtrop JS, Krist AH, et al. Improving multiple health risk behaviors in primary care: lessons from the Prescription for Health Common Measures, Better Outcomes (COMBO) study. J Am Board Fam Med. 2012;25(5):701-11.

41. Kelishadi R, Sarrafzadegan N, Sadri GH, Pashmi R, Mohammadifard N, Tavasoli AA, et al. Short-term results of a community-based program on promoting healthy lifestyle for prevention and control of chronic diseases in a developing country setting: Isfahan Healthy Heart Program. Asia Pac J Public Health. 2011;23(4):518-33.

42. Clouse ML, Mannino D, Curd PR. Investigation of the correlates and effectiveness of a prison-based wellness program. J Correct Health Care. 2012;18(3):184-97.

43. Dendana E, Ghammem R, Sahli J, Maatoug J, Fredj SB, Harrabi I, et al. Clustering of chronic diseases risk factors among adolescents: a quasi-experimental study in Sousse, Tunisia. Int J Adolesc Med Health. 2017.

44. Puska P, Nissinen A, Tuomilehto J, Salonen JT, Koskela K, McAlister A, et al. The community-based strategy to prevent coronary heart disease: conclusions from the ten years of the North Karelia project. Annu Rev Public Health. 1985;6(1):147-93.

45. Gomez-Pardo E, Fernandez-Alvira JM, Vilanova M, Haro D, Martinez R, Carvajal I, et al. A Comprehensive Lifestyle Peer Group-Based Intervention on Cardiovascular Risk Factors: The Randomized Controlled Fifty-Fifty Program. J Am Coll Cardiol. 2016;67(5):476-85.

46. Wendel-Vos GC, Dutman AE, Verschuren WM, Ronckers ET, Ament A, van Assema P, et al. Lifestyle factors of a five-year community-intervention program: the Hartslag Limburg intervention. Am J Prev Med. 2009;37(1):50-6.

47. Baker A, Richmond R, Castle D, Kulkarni J, Kay-Lambkin F, Sakrouge R, et al. Coronary heart disease risk reduction intervention among overweight smokers with a psychotic disorder: pilot trial. Aust N Z J Psychiatry. 2009;43(2):129-35.

48. Holtrop JS, Dosh SA, Torres T, Thum YM. The community health educator referral liaison (CHERL): a primary care practice role for promoting healthy behaviors. Am J Prev Med. 2008;35(5 Suppl):S365-72.

49. Plescia M, Herrick H, Chavis L. Improving health behaviors in an African American community: the Charlotte Racial and Ethnic Approaches to Community Health project. American Journal of Public Health. 2008;98(9):1678-84.

50. Jolly K, Taylor R, Lip G, Greenfield S, Raftery J, Mant J, et al. The Birmingham Rehabilitation Uptake Maximisation Study (BRUM). Home-based compared with hospital-based cardiac rehabilitation in a multi-ethnic population: cost-effectiveness and patient adherence. Health Technology Assessment (Winchester, England). 2007;11(35):1-118.

51. Wang Y, Xie B, Tao Y, Ma Y, Zhang K. Impact of Community-Based Integrated Traditional Chinese and Western Medicine Metabolic Syndrome Intervention Technology in Rural Residents in Southern Jiangsu, China. Med Sci Monit. 2015;21:2163-9.

52. Becker DM, Yanek LR, Johnson WR, Jr., Garrett D, Moy TF, Reynolds SS, et al. Impact of a community-based multiple risk factor intervention on cardiovascular risk in black families with a history of premature coronary disease. Circulation. 2005;111(10):1298-304.

53. Ofori SN, Kotseva K. Comparison of treatment outcomes in patients with and without diabetes mellitus attending a multidisciplinary cardiovascular prevention programme (a retrospective analysis of the EUROACTION trial). BMC Cardiovasc Disord. 2015;15:11.

54. Toobert DJ, Glasgow RE, Strycker LA, Barrera M, Ritzwoller DP, Weidner G. Long-term effects of the Mediterranean lifestyle program: a randomized clinical trial for postmenopausal women with type 2 diabetes. International Journal of Behavioral Nutrition and Physical Activity. 2007;4(1):1.

55. Johnston D, Tough S, Siever J. The Community Perinatal Care Study: Home Visiting and Nursing Support for Pregnant Women. Zero to Three (J). 2006;27(2):11-7.

56. Reid RD, McDonnell LA, Riley DL, Mark AE, Mosca L, Beaton L, et al. Effect of an intervention to improve the cardiovascular health of family members of patients with coronary artery disease: a randomized trial. Canadian Medical Association Journal. 2013:cmaj. 130550.

57. Hjarnoe L, Leppin A. Health promotion in the Danish maritime setting: challenges and possibilities for changing lifestyle behavior and health among seafarers. BMC Public Health. 2013;13(1):1165.

58. Kubilius R, Jasiukevičienė L, Grižas V, Kubilienė L, Jakubsevičienė E, Vasiliauskas D. The impact of complex cardiac rehabilitation on manifestation of risk factors in patients with coronary heart disease. Medicina. 2012;48(3):24.

59. Grunfeld E, Manca D, Moineddin R, Thorpe KE, Hoch JS, Campbell-Scherer D, et al. Improving chronic disease prevention and screening in primary care: results of the BETTER pragmatic cluster randomized controlled trial. BMC family practice. 2013;14(1):175.

60. De Vries H, Kremers S, Smeets T, Brug J, Eijmael K. The effectiveness of tailored feedback and action plans in an intervention addressing multiple health behaviors. American Journal of Health Promotion. 2008;22(6):417-24.

61. Salminen M, Isoaho R, Vahlberg T, Ojanlatva A, Irjala K, Kivelä S-L. Effects of health advocacy, counseling, and activation among older coronary heart disease (CHD) patients. Aging clinical and experimental research. 2005;17(6):472-8.

62. Carleton RA, Lasater TM, Assaf AR, Feldman HA, McKinlay S. The Pawtucket Heart Health Program: community changes in cardiovascular risk factors and projected disease risk. American journal of public health. 1995;85(6):777-85.

63. Niederhauser VP, Maddock J, LeDoux F, Arnold M. Building strong and ready army families: A multirisk reduction health promotion pilot study. Military Medicine. 2005;170(3):227-33.

64. Siddiqui FR, Shahid A. Promoting healthy workplaces Health pledges initiative at North Kirklees Primary Care Trust, NHS, England. Journal of the Pakistan Medical Association. 2012;62:1028-32.

65. Sadeghi M, Aghdak P, Motamedi N, Tavassoli A, Kelishadi R, Sarrafzadegan N. Do intervention strategies of women healthy heart project (WHHP) impact on differently on working and housewives? ARYA atherosclerosis. 2011;6(4):129.

66. O’brien LM, Polacsek M, MacDonald PB, Ellis J, Berry S, Martin M. Impact of a school health coordinator intervention on health‐related school policies and student behavior. Journal of School Health. 2010;80(4):176-85.

67. Frank E, Elon L, Hertzberg V. A quantitative assessment of a 4-year intervention that improved patient counseling through improving medical student health. Medscape General Medicine. 2007;9(2):58.

68. Schumacher A, Peersen K, Sommervoll L, Seljeflot I, Arnesen H, Otterstad JE. Physical performance is associated with markers of vascular inflammation in patients with coronary heart disease. European Journal of Cardiovascular Prevention & Rehabilitation. 2006;13(3):356-62.

69. Friesen CA. Operation Wellness: a university/community collaboration to enhance adult wellness. Family and Consumer Sciences Research Journal. 2010;39(2):152-60.

70. Saraf DS, Gupta SK, Pandav CS, Nongkinrih B, Kapoor SK, Pradhan SK, et al. Effectiveness of a school based intervention for prevention of non-communicable diseases in middle school children of rural North India: a randomized controlled trial. Indian J Pediatr. 2015;82(4):354-62.

71. Lando HA, Pechacek TF, Pirie PL, Murray DM, Mittelmark MB, Lichtenstein E, et al. Changes in adult cigarette smoking in the Minnesota Heart Health Program. American Journal of Public Health. 1995;85(2):201-8.

72. Drevenhorn E, Kjellgren KI, Bengtson A. Outcomes following a programme for lifestyle changes with people with hypertension. J Clin Nurs. 2007;16(7B):144-51.

73. Hughes GH, Hymowitz N, Ockene JK, Simon N, Vogt TM. The multiple risk factor intervention trial (MRFIT): V. Intervention on smoking. Preventive medicine. 1981;10(4):476-500.

74. Kronish IM, Rieckmann N, Burg MM, Edmondson D, Schwartz JE, Davidson KW. The effect of enhanced depression care on adherence to risk-reducing behaviors after acute coronary syndromes: findings from the COPES trial. Am Heart J. 2012;164(4):524-9.

75. Laaksonen E, Vuoristo-Myllys S, Koski-Jannes A, Alho H. Combining medical treatment and CBT in treating alcohol-dependent patients: effects on life quality and general well-being. Alcohol Alcohol. 2013;48(6):687-93.

76. Nguyen QN, Pham ST, Nguyen VL, Weinehall L, Wall S, Bonita R, et al. Effectiveness of community-based comprehensive healthy lifestyle promotion on cardiovascular disease risk factors in a rural Vietnamese population: a quasi-experimental study. BMC cardiovascular disorders. 2012;12(1):56.

77. van Lieshout J, Huntink E, Koetsenruijter J, Wensing M. Tailored implementation of cardiovascular risk management in general practice: a cluster randomized trial. Implementation Science. 2015;11(1):115.

78. Almeida OP, Marsh K, Murray K, Hickey M, Sim M, Ford A, et al. Reducing depression during the menopausal transition with health coaching: Results from the healthy menopausal transition randomised controlled trial. Maturitas. 2016;92:41-8.

79. Baker AL, Richmond R, Kay-Lambkin FJ, Filia SL, Castle D, Williams JM, et al. Randomized Controlled Trial of a Healthy Lifestyle Intervention Among Smokers With Psychotic Disorders. Nicotine Tob Res. 2015;17(8):946-54.

80. Broekhuizen K, van Poppel MN, Koppes LL, Kindt I, Brug J, van Mechelen W. Can multiple lifestyle behaviours be improved in people with familial hypercholesterolemia? Results of a parallel randomised controlled trial. PLoS One. 2012;7(12):e50032.

81. Butler CC, Simpson SA, Hood K, Cohen D, Pickles T, Spanou C, et al. Training practitioners to deliver opportunistic multiple behaviour change counselling in primary care: a cluster randomised trial. Bmj. 2013;346:f1191.

82. Cameron D, Epton T, Norman P, Sheeran P, Harris PR, Webb TL, et al. A theory-based online health behaviour intervention for new university students (U@Uni:LifeGuide): results from a repeat randomized controlled trial. Trials. 2015;16:555.

83. Connolly S, Kotseva K, Jennings C, Atrey A, Jones J, Brown A, et al. Outcomes of an integrated community-based nurse-led cardiovascular disease prevention programme. Heart. 2017;103(11):840-7.

84. Cueto-Manzano A, Martinez-Ramirez H, Cortes-Sanabria L. Management of chronic kidney disease: primary health-care setting, self-care and multidisciplinary approach. Clinical nephrology. 2010;74:S99-104.

85. Echouffo-Tcheugui JB, Simmons RK, Prevost AT, Williams KM, Kinmonth AL, Wareham NJ, et al. Long-term effect of population screening for diabetes on cardiovascular morbidity, self-rated health, and health behavior. Ann Fam Med. 2015;13(2):149-57.

86. Emmons KM, McBride CM, Puleo E, Pollak KI, Clipp E, Kuntz K, et al. Project PREVENT: a randomized trial to reduce multiple behavioral risk factors for colon cancer. Cancer Epidemiology and Prevention Biomarkers. 2005;14(6):1453-9.

87. Hatzis CM, Papandreou C, Kafatos AG. School health education programs in Crete: evaluation of behavioural and health indices a decade after initiation. Prev Med. 2010;51(3-4):262-7.

88. Hawkes AL, Chambers SK, Pakenham KI, Patrao TA, Baade PD, Lynch BM, et al. Effects of a telephone-delivered multiple health behavior change intervention (CanChange) on health and behavioral outcomes in survivors of colorectal cancer: a randomized controlled trial. J Clin Oncol. 2013;31(18):2313-21.

89. Hjorth P, Medici CR, Juel A, Madsen NJ, Vandborg K, Munk-Jorgensen P. Improving quality of life and physical health in patients with schizophrenia: A 30-month program carried out in a real-life setting. Int J Soc Psychiatry. 2017;63(4):287-96.

90. Hyman DJ, Pavlik VN, Taylor WC, Goodrick GK, Moye L. Simultaneous vs sequential counseling for multiple behavior change. Archives of Internal Medicine. 2007;167(11):1152-8.

91. Ibfelt E, Rottmann N, Kjaer T, Hoybye MT, Ross L, Frederiksen K, et al. No change in health behavior, BMI or self-rated health after a psychosocial cancer rehabilitation: Results of a randomized trial. Acta Oncol. 2011;50(2):289-98.

92. IJzelenberg W, Hellemans IM, van Tulder MW, Heymans MW, Rauwerda JA, van Rossum AC, et al. The effect of a comprehensive lifestyle intervention on cardiovascular risk factors in pharmacologically treated patients with stable cardiovascular disease compared to usual care: a randomised controlled trial. BMC cardiovascular disorders. 2012;12(1):71.

93. Kloek GC, van Lenthe FJ, van Nierop PW, Koelen MA, Mackenbach JP. Impact evaluation of a Dutch community intervention to improve health-related behaviour in deprived neighbourhoods. Health Place. 2006;12(4):665-77.

94. Lakerveld J, Bot SD, Chinapaw MJ, van Tulder MW, Kostense PJ, Dekker JM, et al. Motivational interviewing and problem solving treatment to reduce type 2 diabetes and cardiovascular disease risk in real life: a randomized controlled trial. International Journal of Behavioral Nutrition and Physical Activity. 2013;10(1):47.

95. McClure JB, Catz SL, Ludman EJ, Richards J, Riggs K, Grothaus L. Feasibility and acceptability of a multiple risk factor intervention: The Step Up randomized pilot trial. BMC public health. 2011;11(1):167.

96. Merrill RM, Anderson A, Thygerson SM. Effectiveness of a worksite wellness program on health behaviors and personal health. J Occup Environ Med. 2011;53(9):1008-12.

97. Parekh S, King D, Boyle FM, Vandelanotte C. Randomized controlled trial of a computer-tailored multiple health behaviour intervention in general practice: 12-month follow-up results. International Journal of Behavioral Nutrition and Physical Activity. 2014;11(1):41.

98. Prochaska JO, Butterworth S, Redding CA, Burden V, Perrin N, Leo M, et al. Initial efficacy of MI, TTM tailoring and HRI's with multiple behaviors for employee health promotion. Prev Med. 2008;46(3):226-31.

99. Rosenberg D, Lin E, Peterson D, Ludman E, Von Korff M, Katon W. Integrated medical care management and behavioral risk factor reduction for multicondition patients: behavioral outcomes of the TEAMcare trial. Gen Hosp Psychiatry. 2014;36(2):129-34.

100. Shlay JC, Barber B, Mickiewicz T, Maravi M, Drisko J, Estacio R, et al. Peer Reviewed: Reducing Cardiovascular Disease Risk Using Patient Navigators, Denver, Colorado, 2007-2009. Preventing chronic disease. 2011;8(6).

101. Soderman E, Lisspers J, Sundin O. Impact of depressive mood on lifestyle changes in patients with coronary artery disease. J Rehabil Med. 2007;39(5):412-7.

102. Sol BG, van der Graaf Y, van Petersen R, Visseren FL. The effect of self-efficacy on cardiovascular lifestyle. Eur J Cardiovasc Nurs. 2011;10(3):180-6.

103. Van Dijk MR, Huijgen NA, Willemsen SP, Laven JS, Steegers EA, Steegers-Theunissen RP. Impact of an mHealth Platform for Pregnancy on Nutrition and Lifestyle of the Reproductive Population: A Survey. JMIR Mhealth Uhealth. 2016;4(2):e53.

104. Wister A, Loewen N, Kennedy-Symonds H, McGowan B, McCoy B, Singer J. One-year follow-up of a therapeutic lifestyle intervention targeting cardiovascular disease risk. CMAJ. 2007;177(8):859-65.

105. Woolf SH, Krist AH, Johnson RE, Wilson DB, Rothemich SF, Norman GJ, et al. A practice-sponsored Web site to help patients pursue healthy behaviors: an ACORN study. Ann Fam Med. 2006;4(2):148-52.

106. Arikan I, Metintas S, Kalyoncu C. Application of Healthy Heart program in the two semi-rural areas in Eskisehir. Anadolu Kardiyol Derg. 2011;11(6):485-91.

107. Bradley A, Marshall A, Stonehewer L, Reaper L, Parker K, Bevan-Smith E, et al. Pulmonary rehabilitation programme for patients undergoing curative lung cancer surgery. Eur J Cardiothorac Surg. 2013;44(4):e266-71.

108. Chander JSJU, Prakasam A, Kannan S, Kumar S, Tyagi MG. A study of pharmaceutical care impact on cardiovascular risk in diabetic and hypertensive patients. International Journal of Pharmaceutical Sciences and Research. 2013;4(8):3135.

109. Chaves G, Britez N, Munzinger J, Uhlmann L, Gonzalez G, Oviedo G, et al. Education to a Healthy Lifestyle Improves Symptoms and Cardiovascular Risk Factors - AsuRiesgo Study. Arq Bras Cardiol. 2015;104(5):347-55.

110. Chung M, Melnyk P, Blue D, Renaud D, Breton M-C. Worksite health promotion: the value of the Tune Up Your Heart program. Population health management. 2009;12(6):297-304.

111. Cox CL, McLaughlin RA, Rai SN, Steen BD, Hudson MM. Adolescent survivors: a secondary analysis of a clinical trial targeting behavior change. Pediatr Blood Cancer. 2005;45(2):144-54.

112. Dale H, Watson L, Adair P, Humphris G. Looked after young people: Reducing health inequalities through an evidence- and theory-informed intervention. Health Education Journal. 2016;75(7):811-22.

113. Eckman MH, Wise R, Leonard AC, Dixon E, Burrows C, Khan F, et al. Impact of health literacy on outcomes and effectiveness of an educational intervention in patients with chronic diseases. Patient Educ Couns. 2012;87(2):143-51.

114. Farquhar JW, Fortmann SP, Flora JA, Taylor CB, Haskell WL, Williams PT, et al. Effects of communitywide education on cardiovascular disease risk factors: the Stanford Five-City Project. Jama. 1990;264(3):359-65.

115. Gamble JM, Hoang H, Eurich DT, Jindal KK, Senior PA. Patient-level evaluation of community-based, multifactorial intervention to prevent diabetic nephropathy in northern alberta, Canada. J Prim Care Community Health. 2012;3(2):111-9.

116. Goetzel RZ, Tabrizi M, Henke RM, Benevent R, Brockbank CV, Stinson K, et al. Estimating the return on investment from a health risk management program offered to small Colorado-based employers. J Occup Environ Med. 2014;56(5):554-60.

117. Happell B, Stanton R, Platania-Phung C, McKenna B, Scott D. The cardiometabolic health nurse: physical health behaviour outcomes from a randomised controlled trial. Issues Ment Health Nurs. 2014;35(10):768-75.

118. Jacobs N, De Bourdeaudhuij I, Thijs H, Dendale P, Claes N. Effect of a cardiovascular prevention program on health behavior and BMI in highly educated adults: a randomized controlled trial. Patient Educ Couns. 2011;85(1):122-6.

119. Loeppke R, Nicholson S, Taitel M, Sweeney M, Haufle V, Kessler RC. The impact of an integrated population health enhancement and disease management program on employee health risk, health conditions, and productivity. Popul Health Manag. 2008;11(6):287-96.

120. López ML, Iglesias JM, del Valle MO, Comas Á, Fernández JM, De Vries H, et al. Impact of a primary care intervention on smoking, drinking, diet, weight, sun exposure, and work risk in families with cancer experience. Cancer Causes & Control. 2007;18(5):525-35.

121. Mastrangelo G, Marangi G, Bontadi D, Fadda E, Cegolon L, Bortolotto M, et al. A worksite intervention to reduce the cardiovascular risk: proposal of a study design easy to integrate within Italian organization of occupational health surveillance. BMC Public Health. 2015;15:12.

122. Naser A, Shahamfar J, Kumar GV, Daga M, Hadi HS, Saeed D. Cardiac risk factor changes through an intensive multifactorial life style modification program in CHD patients: results from a two year follow up. J Biol Sci. 2008;8(2):248-57.

123. Neves Â, Alves AJ, Ribeiro F, Gomes JL, Oliveira J. The effect of cardiac rehabilitation with relaxation therapy on psychological, hemodynamic, and hospital admission outcome variables. Journal of cardiopulmonary rehabilitation and prevention. 2009;29(5):304-9.

124. Pawar D, Mojtabai R, Goldman A, Batkis D, Malloy K, Cullen B. Assessment of Response to Providing Health-related Information in a Community Psychiatry Outpatient Setting. J Psychiatr Pract. 2016;22(4):344-7.

125. Pfaeffli Dale L, Whittaker R, Jiang Y, Stewart R, Rolleston A, Maddison R. Text Message and Internet Support for Coronary Heart Disease Self-Management: Results From the Text4Heart Randomized Controlled Trial. J Med Internet Res. 2015;17(10):e237.

126. Saltychev M, Laimi K, El-Metwally A, Oksanen T, Pentti J, Virtanen M, et al. Effectiveness of multidisciplinary early rehabilitation in reducing behaviour-related risk factors. J Rehabil Med. 2012;44(4):370-7.

127. Schilling J, Faisst K, Lee C-Y, Candinas B, Gutzwiller F. The Check Bus Project and its effectiveness on health promotion at work. Journal of occupational health. 2005;47(2):136-42.

128. Schulz DN, Kremers SP, Vandelanotte C, van Adrichem MJ, Schneider F, Candel MJ, et al. Effects of a web-based tailored multiple-lifestyle intervention for adults: a two-year randomized controlled trial comparing sequential and simultaneous delivery modes. J Med Internet Res. 2014;16(1):e26.

129. Shin SA, Kim H, Lee K, Lin V, Liu G, Shin E. Effects of diabetic case management on knowledge, self-management abilities, health behaviors, and health service utilization for diabetes in Korea. Yonsei Med J. 2015;56(1):244-52.

130. Tobari H, Arimoto T, Shimojo N, Yuhara K, Noda H, Yamagishi K, et al. Physician-pharmacist cooperation program for blood pressure control in patients with hypertension: a randomized-controlled trial. Am J Hypertens. 2010;23(10):1144-52.

131. White JC, Hartley S, Ozminkowski RJ. Association Between Corporate Wellness Program Participation and Changes in Health Risks. J Occup Environ Med. 2015;57(10):1119-26.

132. Wills J, Kelly M. What works to encourage student nurses to adopt healthier lifestyles? Findings from an intervention study. Nurse Educ Today. 2017;48:180-4.

133. Salminen M, Vahlberg T, Ojanlatva A, Kivelä S-L. Effects of a controlled family-based health education/counseling intervention. American journal of health behavior. 2005;29(5):395-406.

134. Busch V, De Leeuw JR, Zuithoff NP, Van Yperen TA, Schrijvers AJ. A Controlled Health Promoting School Study in the Netherlands: Effects After 1 and 2 Years of Intervention. Health Promot Pract. 2015;16(4):592-600.

135. Baker AL, Turner A, Kelly PJ, Spring B, Callister R, Collins CE, et al. 'Better Health Choices' by telephone: a feasibility trial of improving diet and physical activity in people diagnosed with psychotic disorders. Psychiatry Res. 2014;220(1-2):63-70.

136. Knekt P, Laaksonen MA, Raitasalo R, Haaramo P, Lindfors O. Changes in lifestyle for psychiatric patients three years after the start of short- and long-term psychodynamic psychotherapy and solution-focused therapy. Eur Psychiatry. 2010;25(1):1-7.

137. Knudsen MD, Hjartaker A, Olsen MK, Hoff G, de Lange T, Bernklev T, et al. Changes in health behavior 1 year after testing negative at a colorectal cancer screening: a randomized-controlled study. Eur J Cancer Prev. 2017.

138. Mai KS, Sandbaek A, Borch-Johnsen K, Lauritzen T. Are lifestyle changes achieved after participation in a screening programme for Type 2 diabetes? The ADDITION Study, Denmark. Diabet Med. 2007;24(10):1121-8.

139. Trovato GM, Pirri C, Martines GF, Tonzuso A, Trovato F, Catalano D. Lifestyle interventions, insulin resistance, and renal artery stiffness in essential hypertension. Clin Exp Hypertens. 2010;32(5):262-9.

140. Ruffin MTt, Nease DE, Jr., Sen A, Pace WD, Wang C, Acheson LS, et al. Effect of preventive messages tailored to family history on health behaviors: the Family Healthware Impact Trial. Ann Fam Med. 2011;9(1):3-11.

141. van den Wijngaart LS, Sieben A, van der Vlugt M, de Leeuw FE, Bredie SJ. A nurse-led multidisciplinary intervention to improve cardiovascular disease profile of patients. West J Nurs Res. 2015;37(6):705-23.
